# Comprehensive analysis of circRNA expression pattern and circRNA-miRNA-mRNA network in the pathogenesis of atherosclerosis in rabbits

**DOI:** 10.18632/aging.101541

**Published:** 2018-09-06

**Authors:** Feng Zhang, Ruyou Zhang, Xiaoyu Zhang, Yingnan Wu, Xiaoying Li, Shuang Zhang, Wenying Hou, Yu Ding, Jiawei Tian, Litao Sun, Xianchao Kong

**Affiliations:** 1 Department of Ultrasound, The 2nd Affiliated Hospital of Harbin Medical University, Harbin 150081, Heilongjiang, China; 2 Department of Neurosurgery, The 2nd Affiliated Hospital of Harbin Medical University, Harbin 150081, Heilongjiang, China; 3 Department of Ultrasound, Drug Rehabilitation Center of Heilongjiang Province, Harbin 150056, Heilongjiang, China; 4 College of Bioinformatics Science and Technology, Harbin Medical University, Harbin 150081, Heilongjiang, China; 5 Department of Gynecology and Obstetrics, The 2nd Affiliated Hospital of Harbin Medical University, Harbin 150081, Heilongjiang, China; * Equal contribution

**Keywords:** atherosclerosis, circular RNA, competing endogenous RNAs, ceRNA network, RNA-seq

## Abstract

Atherosclerosis is a chronic and multifactorial inflammatory disease and is closely associated with cardiovascular and cerebrovascular diseases. circRNAs can act as competing endogenous RNAs to mRNAs and function in various diseases. However, there is little known about the function of circRNAs in atherosclerosis. In this study, three rabbits in the case group were fed a high-fat diet to induce atherosclerosis and another three rabbits were fed a normal diet. To explore the biological functions of circRNAs in atherosclerosis, we analyzed the circRNA, miRNA and mRNA expression profiles using RNA-seq. Many miRNAs, mRNAs and circRNAs were identified as significantly changed in atherosclerosis. We next predicted miRNA-target interactions with the miRanda tool and constructed a differentially expressed circRNA-miRNA-mRNA triple network. A gene ontology enrichment analysis showed that genes in the network were involved in cell adhesion, cell activation and the immune response. Furthermore, we generated a dysregulated circRNA-related ceRNAs network and found seven circRNAs (*ocu-cirR-novel-18038*, *-18298*, *-15993*, *-17934*, *-17879*, *-18036* and *-14389*) were related to atherosclerosis. We found these circRNAs also functioned in cell adhesion, cell activation and the immune response. These results show that the crosstalk between circRNAs and their competing mRNAs might play crucial roles in the development of atherosclerosis.

## Introduction

Atherosclerosis (AS) is a chronic and multifactorial inflammatory disease with high morbidity and mortality worldwide [1]. Moreover, experimental and clinical data suggest that the combined effects of aging and inflammation lead to an increased incidence of atherosclerosis [2]. The main clinical manifestations of atherosclerosis are coronary heart disease, stroke and peripheral vascular disease. Atherosclerosis pathogenesis proceeds through a series of steps including endothelial damage, inflammatory reaction, metabolic disorder, cell proliferation, foam cell formation, and atherosclerotic plaque rupture [3]. Furthermore, it is estimated that approximately 50% of the risk for atherosclerosis is genetically determined [4]. Therefore, in-depth atherosclerosis research is of clinical and theoretical importance.

In recent years, large-scale genome and transcriptome studies have discovered a considerable number of non-coding RNAs (ncRNAs), including microRNAs (miRNAs) and long noncoding RNAs (lncRNAs). Moreover, experimental evidence revealed that ncRNAs, together with coding RNAs, were relevant to atherosclerosis [5–7]. Circular RNAs (circRNAs) are a novel type of endogenous noncoding RNAs that form covalently closed continuous loops without 3′- and 5′- ends [8,9]. Emerging evidence has implicated circRNAs in a wide range of biological processes, and their dysregulated expression is associated with complicated diseases including neurodegenerative disorders and cancers [10,11]. In recent years, researchers have proposed that circRNAs are involved in the development of atherosclerosis. Burd *et al*. discovered *cANRIL*, an antisense circRNA from the *INK4/ARF* locus, whose expression correlated with *INK4/ARF* transcription and atherosclerotic vascular disease risk [12]. Additionally, Chen *et al*. reported that the circRNA *hsa_circ_0003575* was significantly upregulated in oxLDL-induced human umbilical vein endothelial cells. Silencing this circRNA promoted proliferation and angiogenesis in human umbilical vein endothelial cells, which provided novel insights for the circRNA regulation of endothelial cell functions in atherosclerosis [13]. However, the functions of circRNAs and their roles in atherosclerosis are largely unknown.

Recent studies have reported that mRNAs and circRNAs can act as competing endogenous RNAs (ceRNAs), or miRNA sponges, to communicate with each other by competing for miRNA-binding through common miRNA response elements (MREs) [14,15]. For example, Li *et al*. found that *circHIPK3* was significantly downregulated in bladder cancer tissue. Overexpression of *circHIPK3* suppresses heparanase (*HPSE*) expression via sponging *miR-558,* resulting in inhibition of migration, invasion and angiogenesis of bladder cancer cells [16]. Importantly, Chen *et.al* reported that *circRNA_100290* functioned as a ceRNA to regulate the *CDK6* mRNA by competitively binding to the *miR-29* family. Overexpression of *circRNA_100290* upregulated *CDK6* expression by relieving the *miR-29*-family-mediated inhibition of *CDK6,* and this ultimately led to sustaining the cell cycle and proliferation of oral squamous cell carcinomas [17]. Furthermore, analysis of circRNA-related ceRNA networks has been performed in bladder carcinoma [18], osteosarcoma [19], lung cancer [20] and coronary artery disease [21]. However, there is little data on circRNA-associated ceRNA networks in atherosclerosis. To fully understand the impact of ceRNA crosstalk on atherosclerosis, it will be crucial to investigate the circRNA-miRNA-mRNA competitive regulatory networks.

In our study, we investigated the miRNA, circRNA and mRNA expression profiles and identified differentially expressed (DE) RNA species in the atherosclerotic rabbit model by using RNA-seq analysis. Subsequently, we predicted the interactions of DEmiRNA-DEmRNA and DEmiRNA-DEcircRNA with the miRanda tool. According to the ceRNA theory, a DEcircRNA-DEmiRNA-DEmRNA triple network was constructed and gene ontology (GO) enrichment analysis was performed on DEmRNAs to explore the potential regulatory functions of circRNA. Then, a dysregulated circRNA-related ceRNA network (DCCN) was constructed in consideration of the hypergeometric test of circRNAs and mRNAs sharing the same miRNAs. We next investigated the topological properties of the DCCN and discovered a key module. Our findings provide new evidence for understanding the molecular mechanisms of circRNAs in the pathogenesis of AS.

## RESULTS

### Successful established of atherosclerotic rabbit model

**Figure 1:**
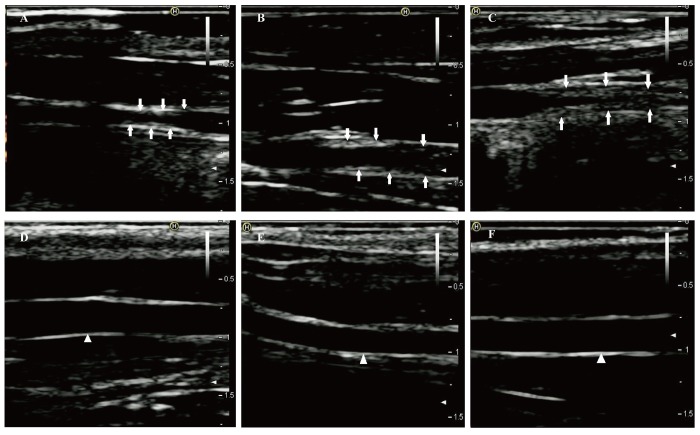
**Two-dimensional ultrasound of right common carotid arteries at eighth week.** (**A-C**) Two-dimensional ultrasound images show that the right common carotid arteries in the case group formed obvious atherosclerotic plaques indicated by the arrows. (**D-F**) Two-dimensional ultrasound images clearly show that the intimas of the right common carotid arteries in the control group remain smooth (white triangle).

**Figure 2:**
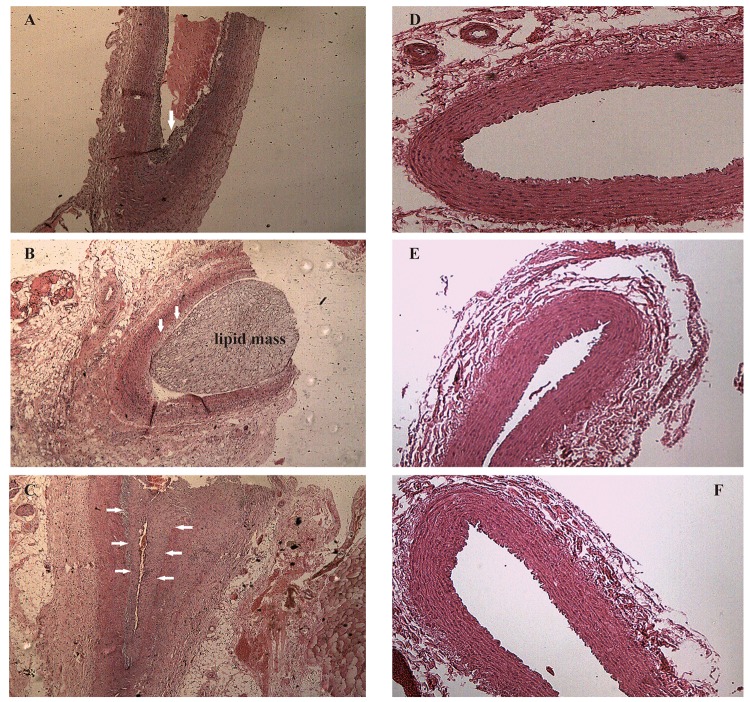
**Hematoxylin and eosin staining of right common carotid arteries.** (**A-C**) Hematoxylin and eosin stained vessels reveal plaques of different severities in the carotid arteries of the case group, ×40 (arrows). (**D-F**) Hematoxylin and eosin stained vessels show that the carotid arteries in the control group without obvious abnormalities, ×40.

After one week of endothelial injury and high-fat diet, atherosclerotic plaques appeared. The atherosclerotic plaques of the right common carotid arteries were easily detected by the 8th week on the 2D-ultrasound images (Figure 1A-C). However, the carotid arteries of rabbits in the control group had no significant changes and the intima remained smooth (Figure 1D-F). Besides, hematoxylin and eosin stained vessels adjacent to the RNA samples revealed plaques of different severities in the carotid arteries of the case group (Figure 2A-C), while there were no obvious abnormalities in the control group (Figure 2D-F).

### Identification of circRNAs in rabbit carotid arteries

**Figure 3:**
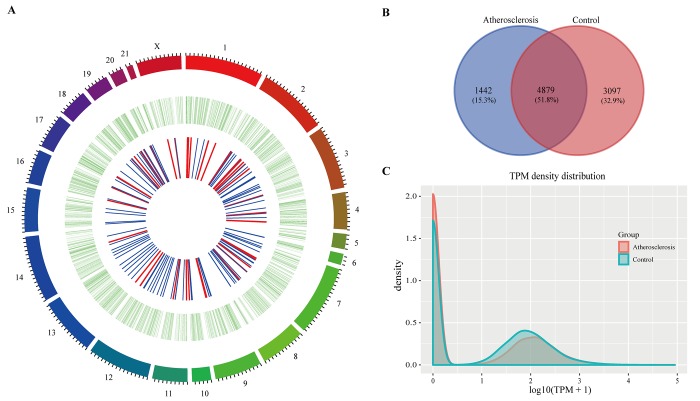
**The circRNAs in rabbit carotid arteries.** (**A**) Circos plot showing circRNAs on rabbit chromosome. The outmost layer of ring is chromosome map of the rabbit genome. The larger inner green ring represents all circRNAs detected by RNA-seq. The smaller inner ring indicates the differentially expressed circRNAs with fold change > 2 and p-Value < 0.05, the up and down regulation circRNAs have been marked in red and blue bars. (**B**) Among detected circRNAs, 4,879 common circRNAs and 1,442 specific circRNAs in AS group and 3,097 specific circRNAs in control group. (**C**) The TPM distributions of circRNAs.

**Figure 4:**
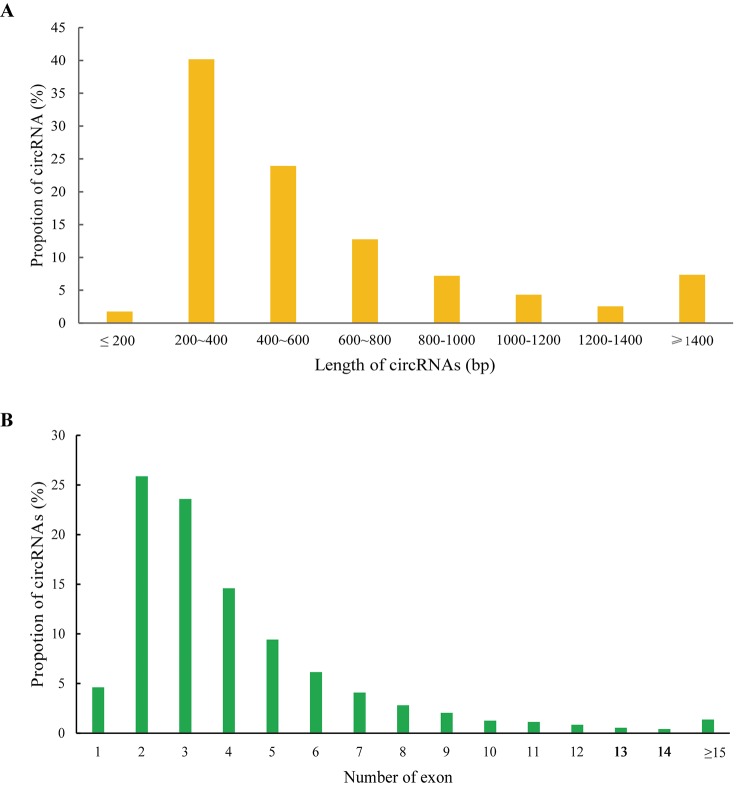
**Features of circRNAs.** Distribution of the sequence length (**A**) and exon number (**B**) of circRNAs.

In the present study, we used CIRCexplorer2 to systematically identify and annotate circRNAs. In total, we identified 9,418 circRNAs from 2 groups, including 9,336 exonic EcircRNAs (99.55%) and 82 intronic IcircRNAs (0.45%) with isoforms that were not previously reported. In addition, the circRNA transcripts were broadly distributed in all chromosomes (Figure 3A). Approximately 8.65%, 8.08% and 8.03% came from chr13, chr1 and chr2, respectively, whereas the percentages of circRNA from any other chromosome was less than 7%. As shown in Figure 3B, 6,321 and 7,976 circRNAs were detected in the AS group and control group, respectively, and 4,879 circRNAs were detected in both groups. We used transcripts per million reads (TPM) to estimate the expression level of the circRNA transcripts. Almost all circRNA transcripts were expressed at low levels (Figure 3C). Sequence length analysis revealed that circRNA transcripts were mostly 200-400 bp in length (Figure 4A). In addition, we found the number of exons in circRNA transcripts was mainly range from 2 to 6 (Figure 4B).

### Identification of differentially expressed RNAs in atherosclerosis

**Figure 5:**
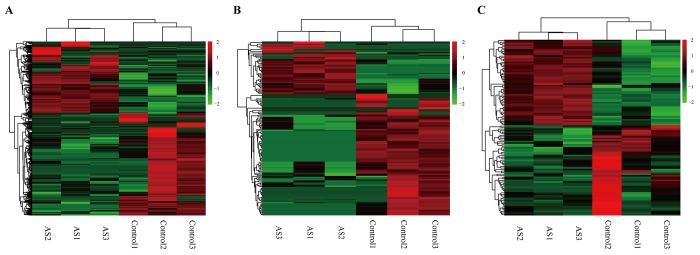
**Heatmap showing expression profiles of different RNAs.** (**A-C**) Hierarchical cluster analysis was used to assess the significantly different expressed mRNA, circRNA and miRNAs, respectively (FoldChange > 2 and PValue < 0.05). Red and green denoted high and low relative expression, respectively. Each RNA was represented by a single row of colored boxes and each sample was presently by a single column.

To investigate the possible biological functions of circRNAs in AS, we analyzed miRNA, circRNA and mRNA expression profile data from AS samples and normal controls. RNAs with a fold-change >2 and p-value ≤ 0.05 were identified as significantly differentially expressed. RNA-seq analysis identified 813 mRNAs aberrantly expressed in AS, including 333 upregulated and 480 downregulated mRNAs (Supplementary Table S1). Meanwhile, 48 and 111 circRNAs were upregulated and downregulated, respectively, in AS samples compared to in controls (Supplementary Table S2). In addition, we found 55 upregulated miRNAs and 58 downregulated miRNAs in AS samples compared to controls (Supplementary Table S3). The top 10 dysregulated mRNAs, circRNAs and miRNAs based on p-value were summarized in Table 1, 2 and 3, respectively. Hierarchical clustering showed that DEmiRNA, DEcircRNA and DEmRNA expression patterns among samples were distinguishable (Figure 5). The data suggested that the expressions of miRNAs, circRNAs and mRNAs in AS are different from those in control samples.

**Table 1:** Top 10 dysregulated mRNAs in atherosclerotic rabbit.

**Transcript**	**Gene_id**	**Gene_name**	**Status**	**P-value**
ENSOCUT00000006046	ENSOCUG00000006045	EZH1	DOWN	7.44E-15
ENSOCUT00000004441	ENSOCUG00000004434	CHD1	DOWN	9.88E-14
ENSOCUT00000012776	ENSOCUG00000012765	CEP192	DOWN	2.50E-13
ENSOCUT00000010246	ENSOCUG00000010247	ATP6V0D2	UP	1.25E-10
ENSOCUT00000008304	ENSOCUG00000008303	MMP12	UP	6.26E-10
ENSOCUT00000008305	ENSOCUG00000008307	LOC100356376	DOWN	8.38E-10
ENSOCUT00000003771	ENSOCUG00000003771	STAM	DOWN	3.36E-09
ENSOCUT00000026768	ENSOCUG00000028052	AMOTL2	UP	7.80E-09
ENSOCUT00000033370	ENSOCUG00000017747	CPNE8	UP	3.52E-08
ENSOCUT00000006543	ENSOCUG00000006539	-	UP	7.24E-08

**Table 2:** Top 10 dysregulated circRNAs in atherosclerotic rabbit.

**CircRNA_id**	**Best_hit_transcript**	**Gene_name**	**Status**	**P-value**
ocu-cirR-novel-15310	ENSOCUT00000009780	TEX14	DOWN	1.95E-06
ocu-cirR-novel-17881	ENSOCUT00000015004	COL3A1	UP	6.00E-06
ocu-cirR-novel-13067	ENSOCUT00000007175	SATB1	DOWN	1.03E-04
ocu-cirR-novel-16238	ENSOCUT00000009541	AFF3	DOWN	1.76E-04
ocu-cirR-novel-10713	ENSOCUT00000009288	ABCA1	UP	3.40E-04
ocu-cirR-novel-12689	ENSOCUT00000015441	MIER1	DOWN	4.38E-04
ocu-cirR-novel-11221	ENSOCUT00000021575	KIF2A	DOWN	4.53E-04
ocu-cirR-novel-16820	ENSOCUT00000008262	SEC24A	DOWN	5.70E-04
ocu-cirR-novel-10714	ENSOCUT00000009288	ABCA1	UP	8.14E-04
ocu-cirR-novel-13631	ENSOCUT00000008150	CENPC	DOWN	9.27E-04

**Table 3:** Top 10 dysregulated miRNAs in atherosclerotic rabbit.

**MiRNA**	**Status**	**logFC**	**P-value**
ocu-miR-12092-5p	UP	4.485	1.45E-07
ocu-miR-196b-5p	DOWN	-6.328	5.61E-06
ocu-miR-424-5p	UP	3.328	1.39E-05
ocu-miR-450a-5p	UP	3.240	3.91E-05
ocu-miR-542-3p	UP	3.283	4.72E-05
ocu-miR-204-5p	DOWN	-2.601	7.37E-05
ocu-miR-128b-3p	DOWN	-3.557	7.88E-05
ocu-miR-128a-3p	DOWN	-3.5567	9.74E-05
ocu-miR-21-5p	UP	2.234	1.04E-04
ocu-miR-34a-5p	UP	2.454	2.30E-04

### Construction of the DEcircRNA-DEmiRNA-DEmRNA triple network

**Figure 6:**
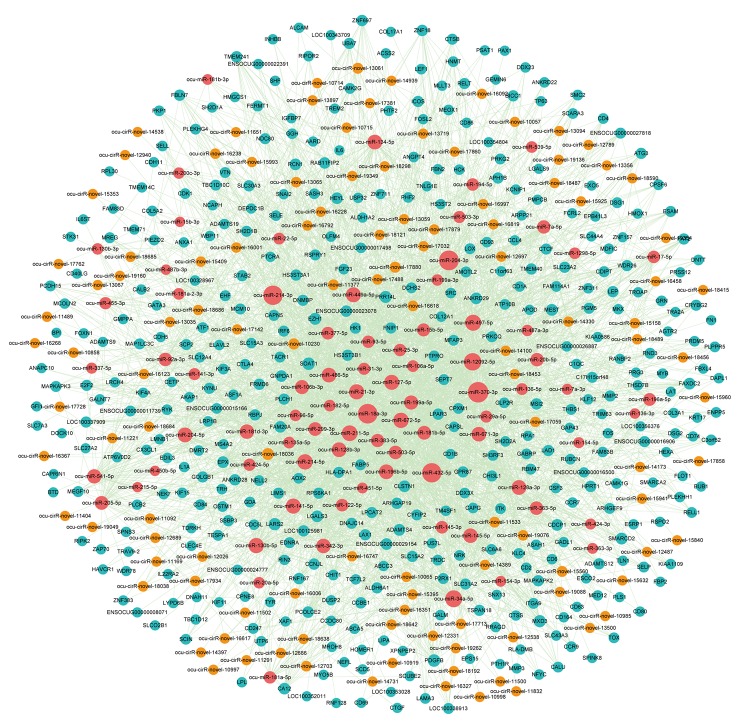
**The view of DEcircRNA-DEmiRNA-DEmRNA triple network.** The network includes 81 miRNAs, 115 circRNAs, 399 mRNAs and 3007 edges. The blue nodes represented mRNA, the red nodes represented miRNAs, and the orange nodes represented circRNAs.

Increasing evidence indicates that circRNAs can sequester relevant miRNAs through MREs to post-transcriptionally regulate gene expression. To investigate ceRNA regulation in atherosclerosis and identify AS-related circRNAs, we identified putative DEmiRNA-DEcircRNA and DEmiRNA-DEmRNA interactions using the miRanda software. In total, we obtained 345 DEmiRNA-DEcircRNA and 2,874 DEmiRNA-DEmRNA interaction pairs. We next extracted miRNAs that paired with both circRNAs and mRNAs to construct the DEcircRNA-DEmiRNA-DEmRNA triple network, which included 81 miRNAs, 115 circRNAs, 399 mRNAs and 3,007 interaction pairs (Figure 6). The degree distribution of the network nodes was investigated. We observed a power-law distribution with a slope of -1.238 and an R-squared value of 0.808. This suggested that the network displayed scale-free characteristics typical of a biological network rather than a random network (Supplementary Figure S1).

**Figure 7:**
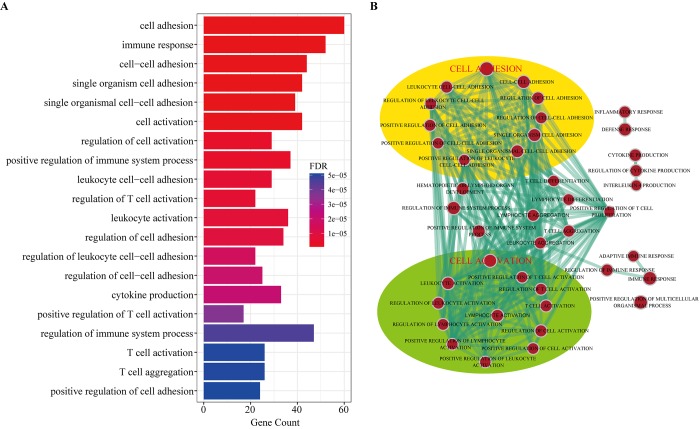
**Gene Ontology (GO) analysis of dysregulated mRNAs in the DEcircRNA-DEmiRNA-DEmRNA triple network.** (**A**) Top 20 enrichment terms of biological processes. (**B**) The enrichment map of GO annotation. Node size represents the number of mRNAs in specific GO term. The edge thickness represents the number of mRNAs shared by two GO term linked by the edge.

To investigate the potential functional implication of the circRNAs, we performed functional enrichment analysis for all genes in the DEcircRNA-DEmiRNA-DEmRNA triple network based on gene ontology biological process terms. These genes were enriched for 41 GO biological terms (GOTERM-BP-FAT) (FDR cutoff =0.01), and Figure 7A shows the top 20 terms. The top ten enriched GO-BP terms, according to the FDR values, included cell adhesion, immune response, cell-cell adhesion, single organism cell adhesion, single organismal cell-cell adhesion, cell activation, regulation of cell activation, positive regulation of immune system process, leukocyte cell-cell adhesion and regulation of T cell activation. We also visualized and clustered the enriched GO-BP terms using the EnrichmentMap plug-in in Cytoscape (Figure 7B). We found that the enriched GO-BP terms were mainly clustered into cell adhesion and cell activation categories. Furthermore, these enriched GO terms have been implicated in many physiological and pathophysiological activities of atherosclerosis. Therefore, the results indicated that circRNAs act as ceRNAs to regulate mRNAs by competing for shared miRNAs in the pathogenesis of atherosclerosis.

Based on the above results, we extracted the genes from the cell adhesion and cell activation categories and their corresponding miRNAs and circRNAs from the DEcircRNA-DEmiRNA-DEmRNA triple network to form sub-network, respectively. In the cell adhesion sub-network, 112 circRNAs and 60 mRNAs competed for 70 miRNAs (Supplementary Figure S2A). Meanwhile, 111 circRNAs and 42 mRNAs competed for 69 miRNAs in the cell activation sub-network (Supplementary Figure S2B). The sub-network indicated that one mRNA might compete with multiple circRNAs. In particular, many circRNAs were predicted to participate in 2 sub-networks at the same time. Therefore, it is reasonable to predict that these circRNAs might contribute to the molecular regulation of atherosclerosis.

### Construction of dysregulated circRNA-related ceRNA network for atherosclerosis

**Figure 8:**
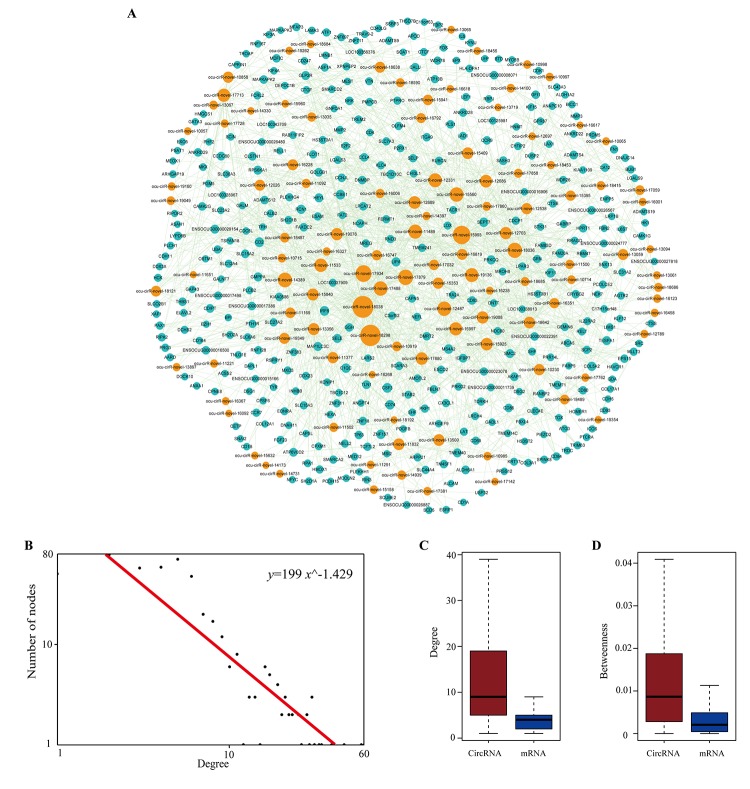
**The layout of dysregulated circRNA related ceRNA network (DCCN) and its structural characteristics.** (**A**) The view of DCCN. The DCCN was comprised of 1452 edges between 365 mRNAs and 112 circRNAs. The blue nodes represented mRNA, the red nodes represented miRNAs, and the orange nodes represented circRNAs. (**B**) Degree distribution of DCCN. (**C-D**) Boxplot of degree and betweenness centrality of mRNAs and circRNAs.

To identify miRNA-mediated DEcircRNA-DEmRNA interactions, a hypergeometric test was used to evaluate the significance of the number of shared miRNAs between circRNA and mRNA pairs. We defined circRNA-mRNA competing pairs if the circRNA and mRNA shared the same miRNA and had a p-value <0.05. The results of the dysregulated circRNA-related ceRNA network (DCCN) was comprised of 1,452 edges between 365 mRNAs and 112 circRNAs (Supplementary Table S4, Figure 8A). The degree of distribution of the nodes in the DCCN approximately followed the power-law distribution with a slope of -1.429 and an R-squared value of 0.807 (Figure 8B). The average degree of the circRNAs was 12.96 and was significantly higher than the average degree of mRNAs (3.98) (p = 2.2e-12, Figure 8C). This indicates that circRNAs have more interactions with other nodes than mRNAs in the DCCN. In addition, we found that circRNA nodes had more betweenness centrality compared to mRNA nodes (p=2.2e-12, Figure 8D), which revealed that circRNAs have more communication functions in the DCCN. These results suggested that, although the circRNAs are non-coding RNA, they exhibit more specific topological properties than mRNAs in the DCCN. These results also revealed that a part of circRNA nodes linked many mRNA nodes in the DCCN and acted as hubs. In the network, the maximum degree node was *ocu-cirR-novel-18038* (degree=60), which is located in the *FN1* gene and competes with many atherosclerosis-related mRNAs.

### Topological analysis of the dysregulated circRNA-related ceRNA network for atherosclerosis

We considered three topological properties of the ceRNA network: the node degree, betweenness centrality (BC) and closeness centrality (CC). In general, a higher degree indicated that the node was a hub that participated in more ceRNA interactions. A higher BC value implied that the node acted as a bridge connecting different network modules. A higher CC value suggested that the node tended to be at the center of the network. Then, we ranked the topological features of all nodes and found that seven circRNAs (*ocu-cirR-novel-18038*, *ocu-cirR-novel-18298*, *ocu-cirR-novel-15993*, *ocu-cirR-novel-17934*, *ocu-cirR-novel-17879*, *ocu-cirR-novel-18036* and *ocu-cirR-novel-14389*) were common nodes that were present in the top 10 of each index described above (Table 4).

**Table 4:** The top seven circRNAs with largest degree, betweenness and closeness in DCCN.

**CircRNA**	**Status**	**Degree**	**Betweenness**	**Closeness**
ocu-cirR-novel-18038	Down	60	0.126	0.345
ocu-cirR-novel-18298	UP	58	0.108	0.345
ocu-cirR-novel-15993	UP	46	0.089	0.340
ocu-cirR-novel-17934	DOWN	39	0.054	0.332
ocu-cirR-novel-17879	UP	34	0.041	0.321
ocu-cirR-novel-18036	UP	33	0.058	0.332
ocu-cirR-novel-14389	UP	31	0.070	0.321

**Figure 9:**
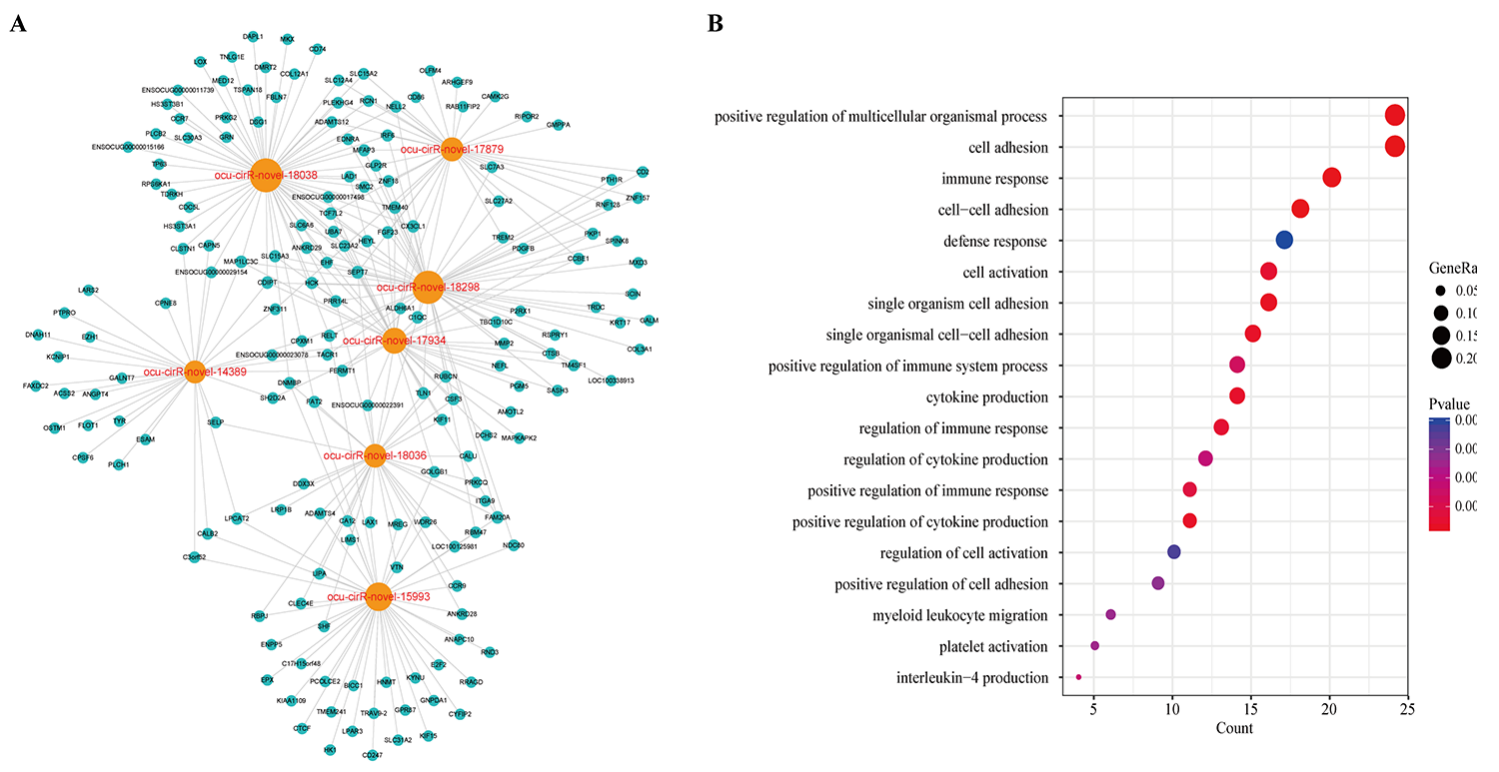
**The key module of DCCN.** (**A**) The ceRNA module network of 7 circRNAs with largest degree, betweenness and closeness in DCCN. The blue nodes represented mRNA and the orange nodes represented circRNAs. (**B**) The gene ontology (GO) enrichment analysis of the module. The enriched GO terms ranked by gene count.

By mapping these circRNAs into a ceRNA network, we found that these circRNAs and their neighbors formed a key module (Figure 9A). Surprisingly, the module contained 176 dysregulated genes and some of them were atherosclerosis-related genes. To explore the potential functional implications of the seven circRNAs, we performed functional enrichment analysis of gene ontology (GO) for the mRNAs in the module (Figure 9B). Interestingly, these genes were also enriched to the immune response, cell adhesion and cell activation terms. Furthermore, we found that these genes were annotated to the platelet activation category. Previous studies have suggested that platelet activation plays a critical role in atherosclerosis. The circulating activated platelets and platelet-leukocyte aggregates promote the formation of atherosclerosis [22]. Platelets may contribute to atherogenesis through the delivery of proinflammatory factors to leukocytes and endothelial cells [23]. Therefore, these seven circRNAs have potential use as diagnostic factors and new potential therapeutic targets of atherosclerosis. However, further specific studies are required to confirm the potential values of these circRNAs.

## DISCUSSION

Atherosclerosis is a chronic inflammatory disease with abundant immune cells involved in the lesions and is the dominant cause of cardiovascular disease [24]. During recent years, great efforts have been made to discover new therapeutic targets and biomarkers of atherosclerosis, but the attention was limited to protein-coding genes, miRNAs and lncRNAs [5–7]. More recently, circRNAs have received attention as new diagnostic markers for diseases including cancers [25]. circRNA expression profiles and functions in AS remain to be determined. circRNAs, miRNAs and mRNAs participate in large-scale ceRNA crosstalk through MREs. This crosstalk has exciting implications for post-transcriptional gene regulation during multiple physiological and pathophysiological processes.

In the present study, we investigated the circRNA, miRNA and mRNA expression profiles of three atherosclerotic rabbits and three controls using RNA-seq. Intriguingly, the expressed circRNAs were widely derived from all chromosomes, including the X chromosome. We found that 159 circRNAs were significantly differentially expressed, including 48 which were upregulated and 111 which were downregulated. At the same time, 813 differentially expressed mRNAs were identified, consisting of 333 upregulated and 480 downregulated mRNAs. In addition, we found 55 upregulated and 58 downregulated miRNAs in atherosclerosis samples. Hierarchical clustering based on the expression value of DERNA demonstrated that atherosclerosis samples clustered together. We also showed that the differentially expressed circRNA transcripts were widely distributed on each chromosome.

Notably, many of the misexpressed genes were well-known atherosclerosis-related genes, including *SELE*, which is highly expressed in atherosclerosis patients [26]. Cholesteryl ester transfer protein (*CETP*) has been shown to be potentially atherogenic. Inhibition of *CETP* activity reduces aortic lesions and inhibits the progression of atherosclerosis in rabbits [27,28]. Insulin-like growth factor I (*IGFI*) appears to be involved in the pathogenesis of atherosclerosis and cardiovascular disease because it stimulates proliferation of vascular smooth muscle cells [29]. Furthermore, increased expression of *MMP12* is associated with elevated expression of *MMP3* and participates in the progression of atherosclerosis in transgenic rabbits [30]. Our findings also suggested that *MMP3* and *MMP12* were highly expressed in the atherosclerotic rabbits. Fibronectin, encoded by the *FN1* gene, is released from platelets and functions in cell attachment, adhesion and spreading, migration, proliferation, differentiation, blood clotting, phagocytosis and atherosclerotic lesions [31–33]. *Hsa-miR-21* is significantly upregulated in human atherosclerotic arteries [34], and we found that *ocu-miR-21-5p* and *ocu-miR-21-3p* were also highly expressed in atherosclerotic rabbits. Wei et al. [35] discovered that downregulation of *miR-145* promotes lesion formation.

To fully understand the impact of the circRNA-related ceRNA crosstalk on atherosclerosis, we used miRNA-mRNA and miRNA-circRNA interaction data predicted by the miRanda tool to construct a DEcircRNA-DEmiRNA-DEmRNA triple network. We performed GO enrichment analysis for the genes in this network and found that the enriched terms were relevant to atherosclerosis. Clustering of the GO terms showed that these genes were mainly enriched in the cell adhesion and cell activation categories. Further, immune response is also an important biological process in atherosclerosis. Atherosclerosis is an inflammatory disease that results in the formation of plaques in large and mid-sized arteries. Both innate- and adaptive-immune responses contribute to the development of atherosclerotic lesions by influencing lipoprotein deposition and oxidation in the arterial wall [36]. The pathological process of atherosclerosis involves the recruitment, adhesion and activation of multiple cells. When the cholesterol-containing low-density lipoproteins accumulate in the intima and activate the endothelium, the atherosclerotic process is initiated [37]. Several types of leukocyte adhesion molecules expressed by activated endothelial cells cause blood cells that normally roll across the vascular surface to adhere to the site of activation [38]. In response to such stimuli, the vascular cell adhesion molecule-1 (VCAM-1) is typically upregulated. Monocytes and lymphocytes carrying counter-receptors for VCAM-1 preferentially adhere to these sites [39]. Activated endothelial cells also produce macrophage colony-stimulating factor, which stimulates monocytes in the intima to differentiate into macrophages. This process is necessary for the development of atherosclerosis [40]. In the intima, the scavenger receptors of macrophages are upregulated which causes uptake of oxLDL. Subsequent accumulation of cholesterol ultimately converts these macrophages into foam cells, which are characteristic of atherosclerotic lesions [41]. T cells are recruited to the forming lesions through a mechanism similar to that of monocytes. However, the activation of T cells is dependent on the recognition of cognate antigens and simultaneous ligation of costimulatory receptors. The activated T cells contribute to local inflammation and growth of the plaque [37]. We thus deduced that these circRNAs might be correlated with atherosclerosis by regulating gene expression.

We next obtained the DEcircRNA-DEmRNA interaction pairs using the hypergeometric test based on the shared miRNAs. Finally, we generated a dysregulated circRNA-related ceRNA network (DCCN), which contained 365 mRNAs, 112 circRNAs and 1,452 edges. Seven circRNAs with high degrees, high betweenness values and high closeness values were found in the DCCN. GO enrichment analysis for the neighbor genes of these circRNAs showed that those mRNAs, which are the ceRNA counterparts of circRNAs, are involved in cell adhesion, immune response, cell activation and platelet activation. These biological processes are associated with the development of atherosclerosis. Therefore, it is reasonable to hypothesize that circRNAs act as ceRNAs to regulate cell adhesion and immune response during the pathogenesis and progression of atherosclerosis.

In conclusion, this is the first report to systematically analyze the dysregulated circRNA-related ceRNA network in atherosclerosis. We characterized a profile of dysregulated circRNAs that might be prospective clinical markers associated with the development of atherosclerosis. However, based on these results, future work is needed to uncover the underlying molecular mechanisms of circRNAs in atherosclerosis.

## MATERIALS AND METHODS

### Animals and tissue preparation

All rabbits (New Zealand white male rabbits) used in our study were obtained from the model animal center of the 2nd Affiliated Hospital of Harbin Medical University. Fan *et al*. reported the feasibility and validity of rabbit models to study human atherosclerosis [42]. We obeyed the principles of laboratory animal care and made efforts to minimize suffering. All procedures were conducted on the basis of the guidelines established by the National Institutes of Health. The Medical Ethics Committee on Animal Research of the 2nd Affiliated Hospital of Harbin Medical University approved of our study protocol (Ethics No. KY2016-090).

Six New Zealand white male rabbits (2.5-3.0 kg) were randomly divided into two groups. The rabbits in the AS group (case group, n=3) were fed a high-fat diet [10% lard (Shandong Shiyuantianjiaji Factory), 1% cholesterol (Shanghai Lanji technology), and 3% yolk powder (Shandong Shiyuantianjiaji Factory)]. The rabbits in the control group (n=3) were fed with a standard diet. The high-fat diet in the AS group was initiated immediately after balloon injury and continued for 8 weeks. Endothelial injury caused by a 2 F Fogarty balloon catheter (Boston Scientific, Temecula, California) was performed to accelerate the formation of atherosclerotic plaque. Rabbits were anaesthetized by intramuscularly injected xylazine (5 mg/kg), ketamine (35 mg/kg) and acepromazine (0.75 mg/kg). Anesthesia was maintained with isoflurane inhalation through a mask during the procedure. As previously reported [43], the balloon-induced endothelial injury of the right common carotid artery was carried out by a balloon catheter introduced through right external carotid artery cut-down. The balloon was gently inflated with 0.3 ml of saline and then retracted. This procedure was performed three times in each rabbit, and then the balloon catheter was removed. The incision was closed with sutures, and the rabbits were allowed to recover.

The progression of atherosclerotic plaques was monitored by 2D-ultrasound weekly. After several weeks, the atherosclerotic plaques of the right common carotid arteries were obvious. Following euthanasia, the rabbits were killed with overdose of pentobarbital sodium. Then, we intercepted 1 cm of the right common carotid artery of each rabbit in the case group with ultrasound guidance where the atherosclerotic plaques were most obvious. Similarly, we intercepted 1cm of the right common carotid artery of each rabbit in the control group. These blood vessels were immediately transferred to liquid nitrogen for preservation. The right common carotid artery adjacent to the incision of each rabbit was swiftly removed. Each specimen was fixed with 4% paraformaldehyde fixative and embedded in paraffin for subsequent hematoxylin and eosin (H&E) staining. Serial cross-sections with a thickness of 3 μm were stained with H&E and observed by light microscopy (Olympus, BX41, Tokyo, Japan).

### Total RNA isolation, library construction and sequencing

Total RNA was isolated from the right common carotid arteries from six samples, including three atherosclerotic sample and three control samples, using TRIzol reagent (Life Technologies, Beijing, China) according to the manufacturer’s recommended protocol. The RNA purity was verified with a NanoPhotometer® spectrophotometer (IMPLEN, CA). The RNA integrity was assessed using the RNA Nano 6000 Assay Kit for the Bioanalyzer 2100 system (Agilent Technologies, CA, USA).

Approximately 8 μg of RNA per sample was used in the protocol to deplete ribosomal RNA according to the instructions in the Epicentre Ribo -zero™ rRNA Removal Kit (Epicentre, USA). The rRNA-depleted RNAs were used as input material for preparing the libraries following the manufacturer’s recommendations in the NEBNext® Ultra™ Directional RNA Library Prep Kit for Illumina (NEB, USA). The products were purified (AMPure XP system), and the library quality was assessed on the Agilent Bioanalyzer 2100 system. Sample clustering was performed on a cBot Cluster Generation System using the TruSeq PE Cluster Kit v3-cBot-HS (Illumina, San Diego, USA) according to the manufacturer’s instructions. Paired-end sequencing with 125-bp reads was performed on the Illumina HiSeq 2500 platform.

A total of 3 μg of RNA per sample was used as input material for the small RNA library. Sequencing libraries were generated using the NEBNextR Multiplex Small RNA Library Prep Set for IlluminaR (NEB, USA) following the manufacturer’s recommendations. Barcodes were added to map the sequences to each sample. The library quality was assessed on the Agilent Bioanalyzer 2100 system using DNA High Sensitivity Chips. Clustering of the barcoded samples was performed on a cBot Cluster Generation System using the TruSeq SR Cluster Kit v3-cBot-HS (Illumina) according to the manufacturer’s instructions. After cluster generation, the libraries were sequenced on an Illumina HiSeq 2500 platform and 50-bp single-end reads were generated.

### RNA-seq data analysis

The quality of the raw sequencing reads was estimated by the FastQC program (http://www.bioinformatics.babraham.ac.uk/projects/fastqc/), which included measures of base quality distribution, average quality of reads and adaptor check. Before alignment, we used Trimmomatic [44] to remove adaptor sequences, trim the 5’ and 3’ end low-quality sequences, and discard the reads containing the N base. This yielded high-quality clean reads for use in all of the downstream analyses.

The reference genome (OryCun2.0) and gene annotation files were downloaded from the Ensembl genome browser (ftp://ftp.ensembl.org/pub/release-92/fasta/oryctolagus_cuniculus/). The clean mRNA reads were aligned to the rabbit genome using the HISAT2 program [45], and the read counts for expressed transcripts were calculated with StringTie software [46]. To identify candidate circular RNAs, we mapped the circRNA-seq reads to the rabbit genome using the read alignment tool Tophat2 [47]. The CIRCexplorer2 [48] program with default parameters was used for transcript assembly, quantification and novel circRNA prediction. Meanwhile, miRDeep2 [49] with default parameters was used to compare the expression of known miRNAs against precursor and mature miRNA sequences in miRBase v22 [50]. Transcripts with read counts of less than 2 in all six samples were removed. The differentially expressed RNAs (DEmRNA, DEmiRNA and DEcircRNA) (P-value ≤ 0.05 and fold change (FC) > 2) were then identified by the ‘edgeR’ R package with default parameters. Hierarchical clustering of the samples was performed to generate an overview of the expression profile characteristics based on the CPM values of differentially expressed RNAs with the ‘pheatmap’ R package.

### Identification of miRNA-target interactions

The miRNA-mRNA and miRNA-circRNA interactions in our study were predicted with miRanda (version 3.3a) [51], which predicted microRNA targets based on sequence complementarity, conserved target sites and free energy of formation. The input 3’UTR sequences of mRNA from the OryCun2.0 assembly were retrieved from the Ensembl database. To improve the reliability of miRNA target prediction, we used a maximum binding free energy of -20 kcal/mol for the miRNA-target interaction predicted in miRanda.

### Identification of AS-related mRNA-circRNA interactions

We next extracted the miRNAs that were predicted to interact with both circRNAs and mRNAs. These miRNAs and their targets were used to construct a DEcircRNA-DEmiRNA-DEmRNA triple network. To identify competing circRNA-mRNA interactions, we used a hypergeometric test which enabled the evaluation of the significance of the shared miRNAs between each circRNA and mRNA. We considered a p-value < 0.05 as statistically significant. The p-value was calculated as follows:

$$p-value=1-\sum_{i=0}^{r-1}\frac{C_{t}^{i}C_{m-i}^{n-i}}{C_{m}^{n}}$$

where *m* is the number of miRNAs in the genome, and *r* represents the number of miRNAs shared between mRNAs and circRNAs. The number of miRNAs interacting with mRNAs and circRNAs is represented by *t* and *n*, respectively.

### Network visualization and topological analysis

We used Cytoscape software (version 3.4.0) [52] to construct and visualize the network in this study. Several topological properties such as the node degree, betweenness and closeness were analyzed using the built-in NetworkAnalyzer tool. The degree of a node is the number of edges that link to this node. Betweenness is a measure of the centrality of the node in a network, which is the number of shortest paths from each node to all others that pass through the node. The closeness of a node is the average length of the shortest path between the node and all other nodes in the network. The Wilcoxon rank-sum test was used to test for significant differences in topological properties. The statistical calculations were performed with R software version 3.3.1. A p-value of less than 0.05 was considered statistically significant.

### Functional enrichment analysis

Functional enrichment analysis at the GO level was performed using the DAVID bioinformatics resource (https://david.ncifcrf.gov/summary.jsp, version 6.8) [53,54]. In our work, GO terms for “Biological Process” (GOTERM-BP-FAT) with a threshold of FDR < 0.05 and p-value < 0.01 were considered significant functional categories using the whole rabbit genome as the background. Functional categories were visualized and clustered using EnrichmentMap [55], a plugin in Cytoscape.

## SUPPLEMENTARY MATERIAL

Supplementary Table S1

Supplementary Table S2

Supplementary Table S3

Supplementary Table S4

Supplementary Figure S1

Supplementary Figure S2
