# Supplementary material for: Comprehensive analysis of circRNA expression pattern and circRNA-miRNA-mRNA network in the pathogenesis of atherosclerosis in rabbits
Source: Aging (Albany NY). 2018 Sep 6;10(9):2266–83. doi: 10.18632/aging.101541 (PMC6188486; doi:10.18632/aging.101541)
Supplement: Supplementary Table S1 [file aging-10-101541-s001.docx]

**Supplementary Table S1.** **Differential expressed mRNA identified by edgeR package.**

| **Transcript** | **Gene_id** | **Gene_name** | **Status** | **logFC** | **Pvalue** |
| --- | --- | --- | --- | --- | --- |
| ENSOCUT00000006046 | ENSOCUG00000006045 | EZH1 | DOWN | -13.75 | 7.44E-15 |
| ENSOCUT00000004441 | ENSOCUG00000004434 | CHD1 | DOWN | -13.52 | 9.88E-14 |
| ENSOCUT00000012776 | ENSOCUG00000012765 | CEP192 | DOWN | -9.51 | 2.50E-13 |
| ENSOCUT00000010246 | ENSOCUG00000010247 | ATP6V0D2 | UP | 6.36 | 1.25E-10 |
| ENSOCUT00000008304 | ENSOCUG00000008303 | MMP12 | UP | 7.06 | 6.26E-10 |
| ENSOCUT00000008305 | ENSOCUG00000008307 | LOC100356376 | DOWN | -14.14 | 8.38E-10 |
| ENSOCUT00000003771 | ENSOCUG00000003771 | STAM | DOWN | -12.95 | 3.36E-09 |
| ENSOCUT00000026768 | ENSOCUG00000028052 | AMOTL2 | UP | 11.58 | 7.80E-09 |
| ENSOCUT00000033370 | ENSOCUG00000017747 | CPNE8 | UP | 11.36 | 3.52E-08 |
| ENSOCUT00000006543 | ENSOCUG00000006539 | ATP2B4 | UP | 5.18 | 7.24E-08 |
| ENSOCUT00000029330 | ENSOCUG00000023926 | ZNF711 | DOWN | -10.82 | 1.23E-07 |
| ENSOCUT00000002871 | ENSOCUG00000002871 | DNTT | DOWN | -11.08 | 1.69E-07 |
| ENSOCUT00000009603 | ENSOCUG00000009603 | FAM114A1 | DOWN | -5.18 | 1.86E-07 |
| ENSOCUT00000001658 | ENSOCUG00000026668 | SNRPC | UP | 7.37 | 4.82E-07 |
| ENSOCUT00000023777 | ENSOCUG00000025590 | LAT | DOWN | -8.19 | 5.14E-07 |
| ENSOCUT00000005597 | ENSOCUG00000005603 | TESPA1 | DOWN | -13.85 | 5.92E-07 |
| ENSOCUT00000012419 | ENSOCUG00000012400 | PTH1R | DOWN | -6.65 | 5.92E-07 |
| ENSOCUT00000003032 | ENSOCUG00000003031 | GGH | UP | 4.20 | 6.39E-07 |
| ENSOCUT00000001143 | ENSOCUG00000001144 | SLC6A6 | UP | 11.18 | 7.74E-07 |
| ENSOCUT00000014066 | ENSOCUG00000014070 | ZNF697 | UP | 8.13 | 8.02E-07 |
| ENSOCUT00000017085 | ENSOCUG00000009828 | ARPP21 | DOWN | -12.23 | 9.99E-07 |
| ENSOCUT00000006833 | ENSOCUG00000006833 | DNAH12 | DOWN | -9.70 | 1.18E-06 |
| ENSOCUT00000013120 | ENSOCUG00000013123 | KRT13 | DOWN | -11.41 | 2.30E-06 |
| ENSOCUT00000017420 | ENSOCUG00000017421 | KRT5 | DOWN | -8.62 | 2.42E-06 |
| ENSOCUT00000013953 | ENSOCUG00000013956 | CD1E | DOWN | -8.37 | 3.20E-06 |
| ENSOCUT00000007840 | ENSOCUG00000007839 | IL6ST | DOWN | -7.95 | 3.28E-06 |
| ENSOCUT00000025743 | ENSOCUG00000022069 | . | DOWN | -10.07 | 3.44E-06 |
| ENSOCUT00000007265 | ENSOCUG00000007265 | TREM2 | UP | 4.81 | 3.56E-06 |
| ENSOCUT00000008263 | ENSOCUG00000008263 | SYTL5 | UP | 10.10 | 3.71E-06 |
| ENSOCUT00000024369 | ENSOCUG00000024887 | CAPRIN1 | UP | 7.37 | 4.06E-06 |
| ENSOCUT00000003849 | ENSOCUG00000003850 | PTCRA | DOWN | -12.67 | 4.29E-06 |
| ENSOCUT00000005008 | ENSOCUG00000005012 | SH2D1A | DOWN | -6.17 | 5.19E-06 |
| ENSOCUT00000017826 | ENSOCUG00000017827 | KLRG1 | UP | 6.35 | 5.64E-06 |
| ENSOCUT00000022494 | ENSOCUG00000002898 | FBLN7 | UP | 4.30 | 5.79E-06 |
| ENSOCUT00000024819 | ENSOCUG00000012012 | PPIB | DOWN | -6.57 | 6.23E-06 |
| ENSOCUT00000031084 | ENSOCUG00000022166 | . | DOWN | -10.89 | 6.48E-06 |
| ENSOCUT00000031010 | ENSOCUG00000023508 | . | UP | 9.83 | 6.68E-06 |
| ENSOCUT00000023313 | ENSOCUG00000017198 | TUB | DOWN | -10.07 | 7.54E-06 |
| ENSOCUT00000032174 | ENSOCUG00000027626 | CD247 | DOWN | -6.31 | 9.68E-06 |
| ENSOCUT00000006739 | ENSOCUG00000008790 | KMT2E | DOWN | -11.93 | 1.04E-05 |
| ENSOCUT00000014250 | ENSOCUG00000014250 | TRDC | DOWN | -7.22 | 1.05E-05 |
| ENSOCUT00000026747 | ENSOCUG00000021580 | FGF23 | UP | 7.89 | 1.84E-05 |
| ENSOCUT00000033718 | ENSOCUG00000029154 | . | DOWN | -10.78 | 1.84E-05 |
| ENSOCUT00000020904 | ENSOCUG00000024415 | . | UP | 6.62 | 1.88E-05 |
| ENSOCUT00000016466 | ENSOCUG00000016469 | MLANA | UP | 4.07 | 2.13E-05 |
| ENSOCUT00000025546 | ENSOCUG00000022318 | . | DOWN | -6.73 | 2.42E-05 |
| ENSOCUT00000025808 | ENSOCUG00000002369 | ITK | DOWN | -10.20 | 2.57E-05 |
| ENSOCUT00000014251 | ENSOCUG00000014252 | MAPKAPK2 | DOWN | -5.59 | 2.84E-05 |
| ENSOCUT00000026659 | ENSOCUG00000027505 | SIT1 | DOWN | -5.69 | 3.09E-05 |
| ENSOCUT00000002371 | ENSOCUG00000002369 | ITK | DOWN | -5.42 | 3.12E-05 |
| ENSOCUT00000011632 | ENSOCUG00000011633 | MYB | DOWN | -13.24 | 3.16E-05 |
| ENSOCUT00000009863 | ENSOCUG00000009863 | PRKCQ | DOWN | -5.04 | 3.21E-05 |
| ENSOCUT00000004218 | ENSOCUG00000004218 | CD14 | UP | 3.45 | 3.44E-05 |
| ENSOCUT00000011410 | ENSOCUG00000011410 | CAPSL | DOWN | -7.53 | 3.95E-05 |
| ENSOCUT00000009212 | ENSOCUG00000009212 | CD1A | DOWN | -9.09 | 3.99E-05 |
| ENSOCUT00000033899 | ENSOCUG00000017563 | AKAP1 | DOWN | -7.52 | 4.08E-05 |
| ENSOCUT00000001249 | ENSOCUG00000001250 | EDNRA | UP | 3.55 | 4.54E-05 |
| ENSOCUT00000009700 | ENSOCUG00000009701 | CSF3 | UP | 7.00 | 4.63E-05 |
| ENSOCUT00000034162 | ENSOCUG00000001964 | LARS2 | DOWN | -11.19 | 4.79E-05 |
| ENSOCUT00000033804 | ENSOCUG00000010972 | LOC100328967 | UP | 4.37 | 5.12E-05 |
| ENSOCUT00000024741 | ENSOCUG00000025851 | CPM | UP | 3.64 | 5.51E-05 |
| ENSOCUT00000015004 | ENSOCUG00000014988 | COL3A1 | UP | 7.70 | 5.62E-05 |
| ENSOCUT00000022727 | ENSOCUG00000024139 | TRA2A | UP | 4.33 | 5.75E-05 |
| ENSOCUT00000007763 | ENSOCUG00000007765 | PSAT1 | DOWN | -4.06 | 5.79E-05 |
| ENSOCUT00000015222 | ENSOCUG00000015224 | PHF12 | DOWN | -9.97 | 6.28E-05 |
| ENSOCUT00000001490 | ENSOCUG00000001490 | IFNLR1 | DOWN | -9.31 | 6.28E-05 |
| ENSOCUT00000029428 | ENSOCUG00000001142 | SNX13 | DOWN | -11.78 | 6.63E-05 |
| ENSOCUT00000008471 | ENSOCUG00000008474 | CD2 | DOWN | -5.11 | 6.65E-05 |
| ENSOCUT00000014441 | ENSOCUG00000027815 | LOC100338913 | UP | 3.51 | 6.66E-05 |
| ENSOCUT00000031968 | ENSOCUG00000021336 | . | DOWN | -9.91 | 7.00E-05 |
| ENSOCUT00000000762 | ENSOCUG00000000763 | PLA2G7 | UP | 3.29 | 7.08E-05 |
| ENSOCUT00000015877 | ENSOCUG00000015885 | MCM9 | DOWN | -4.35 | 7.14E-05 |
| ENSOCUT00000011578 | ENSOCUG00000011573 | LOC100343709 | UP | 7.11 | 7.55E-05 |
| ENSOCUT00000006334 | ENSOCUG00000006337 | TRH | DOWN | -10.10 | 7.88E-05 |
| ENSOCUT00000006194 | ENSOCUG00000006194 | KYNU | UP | 9.80 | 8.14E-05 |
| ENSOCUT00000003063 | ENSOCUG00000003049 | COL17A1 | DOWN | -10.85 | 8.23E-05 |
| ENSOCUT00000000577 | ENSOCUG00000000577 | RSPRY1 | UP | 10.80 | 8.33E-05 |
| ENSOCUT00000006875 | ENSOCUG00000006872 | EMILIN1 | UP | 4.08 | 8.40E-05 |
| ENSOCUT00000011082 | ENSOCUG00000011085 | CTSS | UP | 3.14 | 8.96E-05 |
| ENSOCUT00000006500 | ENSOCUG00000006498 | DSG1 | DOWN | -5.27 | 8.97E-05 |
| ENSOCUT00000017494 | ENSOCUG00000017498 | ISOC1 | DOWN | -9.53 | 9.55E-05 |
| ENSOCUT00000015617 | ENSOCUG00000015622 | METTL7A | DOWN | -5.83 | 9.68E-05 |
| ENSOCUT00000006701 | ENSOCUG00000006697 | HK1 | DOWN | -10.98 | 0.0001 |
| ENSOCUT00000013781 | ENSOCUG00000013779 | FOXN1 | DOWN | -9.90 | 0.0001 |
| ENSOCUT00000021897 | ENSOCUG00000025898 | BICDL1 | DOWN | -9.17 | 0.0001 |
| ENSOCUT00000033172 | ENSOCUG00000017386 | RPL30 | DOWN | -8.75 | 0.0001 |
| ENSOCUT00000021409 | ENSOCUG00000023623 | . | DOWN | -9.85 | 0.0001 |
| ENSOCUT00000033940 | ENSOCUG00000017817 | AHSA2P | DOWN | -8.57 | 0.0001 |
| ENSOCUT00000027837 | ENSOCUG00000011937 | KIF3A | DOWN | -11.16 | 0.0001 |
| ENSOCUT00000016862 | ENSOCUG00000016853 | JAK2 | UP | 4.67 | 0.0001 |
| ENSOCUT00000009883 | ENSOCUG00000009882 | NCAPH | UP | 4.86 | 0.0001 |
| ENSOCUT00000027991 | ENSOCUG00000017438 | SELE | UP | 3.92 | 0.0001 |
| ENSOCUT00000010581 | ENSOCUG00000010583 | FBP2 | DOWN | -9.46 | 0.0001 |
| ENSOCUT00000022897 | ENSOCUG00000022666 | . | DOWN | -14.16 | 0.0001 |
| ENSOCUT00000010737 | ENSOCUG00000010732 | CYFIP1 | UP | 10.60 | 0.0001 |
| ENSOCUT00000010011 | ENSOCUG00000010009 | EPX | DOWN | -5.32 | 0.0001 |
| ENSOCUT00000008069 | ENSOCUG00000008071 | . | DOWN | -6.63 | 0.0001 |
| ENSOCUT00000000600 | ENSOCUG00000029569 | KRT17 | DOWN | -7.56 | 0.0002 |
| ENSOCUT00000002920 | ENSOCUG00000002924 | LGALS3 | UP | 3.76 | 0.0002 |
| ENSOCUT00000010227 | ENSOCUG00000010203 | TLN1 | UP | 7.08 | 0.0002 |
| ENSOCUT00000033318 | ENSOCUG00000017021 | SLC30A3 | DOWN | -10.43 | 0.0002 |
| ENSOCUT00000000579 | ENSOCUG00000000579 | TMEM156 | DOWN | -5.59 | 0.0002 |
| ENSOCUT00000015832 | ENSOCUG00000015833 | SAMSN1 | DOWN | -7.12 | 0.0002 |
| ENSOCUT00000014721 | ENSOCUG00000014730 | FZD2 | UP | 11.46 | 0.0002 |
| ENSOCUT00000001717 | ENSOCUG00000001717 | SKAP1 | DOWN | -4.95 | 0.0002 |
| ENSOCUT00000024824 | ENSOCUG00000015493 | ANXA1 | DOWN | -4.94 | 0.0002 |
| ENSOCUT00000028603 | ENSOCUG00000026988 | CHI3L1 | UP | 3.34 | 0.0002 |
| ENSOCUT00000011270 | ENSOCUG00000011270 | CD1B | DOWN | -7.72 | 0.0002 |
| ENSOCUT00000009312 | ENSOCUG00000009311 | CD84 | UP | 3.31 | 0.0002 |
| ENSOCUT00000006342 | ENSOCUG00000006343 | KLF9 | DOWN | -6.24 | 0.0002 |
| ENSOCUT00000001750 | ENSOCUG00000001751 | RND3 | DOWN | -3.56 | 0.0002 |
| ENSOCUT00000017020 | ENSOCUG00000017021 | SLC30A3 | DOWN | -10.95 | 0.0002 |
| ENSOCUT00000000787 | ENSOCUG00000000788 | GADL1 | DOWN | -4.98 | 0.0002 |
| ENSOCUT00000000534 | ENSOCUG00000000535 | ADTRP | DOWN | -8.60 | 0.0002 |
| ENSOCUT00000009680 | ENSOCUG00000009680 | HOMER1 | UP | 7.65 | 0.0002 |
| ENSOCUT00000010423 | ENSOCUG00000010425 | FABP5 | UP | 2.90 | 0.0002 |
| ENSOCUT00000025837 | ENSOCUG00000015897 | ASF1A | DOWN | -13.25 | 0.0003 |
| ENSOCUT00000009190 | ENSOCUG00000009195 | CLEC4E | UP | 3.42 | 0.0003 |
| ENSOCUT00000025155 | ENSOCUG00000025091 | AARD | DOWN | -7.12 | 0.0003 |
| ENSOCUT00000017819 | ENSOCUG00000017821 | GATA3 | DOWN | -4.31 | 0.0003 |
| ENSOCUT00000029125 | ENSOCUG00000008167 | CALU | UP | 3.25 | 0.0003 |
| ENSOCUT00000033180 | ENSOCUG00000002480 | LOC100343144 | UP | 6.33 | 0.0003 |
| ENSOCUT00000016946 | ENSOCUG00000016942 | ALDH1A2 | UP | 10.22 | 0.0003 |
| ENSOCUT00000016580 | ENSOCUG00000016580 | CTSB | UP | 3.02 | 0.0003 |
| ENSOCUT00000004395 | ENSOCUG00000004398 | DCSTAMP | UP | 6.53 | 0.0003 |
| ENSOCUT00000025407 | ENSOCUG00000001165 | RANBP2 | UP | 13.24 | 0.0003 |
| ENSOCUT00000006720 | ENSOCUG00000006719 | LPCAT2 | UP | 4.79 | 0.0003 |
| ENSOCUT00000003785 | ENSOCUG00000003786 | . | DOWN | -9.14 | 0.0003 |
| ENSOCUT00000025835 | ENSOCUG00000013211 | . | UP | 8.42 | 0.0003 |
| ENSOCUT00000009873 | ENSOCUG00000009864 | SNRNP200 | DOWN | -7.00 | 0.0003 |
| ENSOCUT00000029930 | ENSOCUG00000023896 | E2F2 | DOWN | -4.05 | 0.0003 |
| ENSOCUT00000031760 | ENSOCUG00000024777 | . | DOWN | -6.49 | 0.0003 |
| ENSOCUT00000012911 | ENSOCUG00000012911 | CD4 | DOWN | -4.23 | 0.0003 |
| ENSOCUT00000002829 | ENSOCUG00000002835 | CTGF | DOWN | -12.83 | 0.0003 |
| ENSOCUT00000013683 | ENSOCUG00000013680 | PHF1 | UP | 3.62 | 0.0003 |
| ENSOCUT00000023813 | ENSOCUG00000012578 | CAMK2G | UP | 10.04 | 0.0004 |
| ENSOCUT00000005932 | ENSOCUG00000005926 | USP4 | UP | 7.01 | 0.0004 |
| ENSOCUT00000023601 | ENSOCUG00000027471 | IL6 | UP | 5.86 | 0.0004 |
| ENSOCUT00000000159 | ENSOCUG00000000159 | RGMB | DOWN | -3.76 | 0.0004 |
| ENSOCUT00000004027 | ENSOCUG00000004027 | TRAF3IP3 | DOWN | -4.60 | 0.0004 |
| ENSOCUT00000028683 | ENSOCUG00000010144 | TTC22 | DOWN | -10.16 | 0.0004 |
| ENSOCUT00000007383 | ENSOCUG00000007383 | CCNT1 | DOWN | -6.39 | 0.0004 |
| ENSOCUT00000015494 | ENSOCUG00000015492 | GLP2R | UP | 4.25 | 0.0004 |
| ENSOCUT00000015227 | ENSOCUG00000029337 | MMP3 | UP | 6.28 | 0.0004 |
| ENSOCUT00000003921 | ENSOCUG00000003921 | GLIPR1 | UP | 2.95 | 0.0004 |
| ENSOCUT00000033999 | ENSOCUG00000029200 | . | DOWN | -8.23 | 0.0004 |
| ENSOCUT00000012450 | ENSOCUG00000012455 | MDFIC | DOWN | -4.11 | 0.0004 |
| ENSOCUT00000008247 | ENSOCUG00000008226 | FBN2 | UP | 3.55 | 0.0004 |
| ENSOCUT00000024894 | ENSOCUG00000026627 | . | DOWN | -3.61 | 0.0004 |
| ENSOCUT00000022944 | ENSOCUG00000027275 | TM4SF1 | UP | 11.05 | 0.0004 |
| ENSOCUT00000002178 | ENSOCUG00000002176 | PNN | UP | 9.94 | 0.0004 |
| ENSOCUT00000000074 | ENSOCUG00000000074 | HMGB2 | DOWN | -6.91 | 0.0004 |
| ENSOCUT00000022757 | ENSOCUG00000023764 | CCDC80 | UP | 3.58 | 0.0004 |
| ENSOCUT00000026187 | ENSOCUG00000027691 | KRT18 | DOWN | -10.18 | 0.0005 |
| ENSOCUT00000012033 | ENSOCUG00000012034 | RBM47 | UP | 3.71 | 0.0005 |
| ENSOCUT00000007482 | ENSOCUG00000007483 | TBC1D12 | UP | 5.94 | 0.0005 |
| ENSOCUT00000011422 | ENSOCUG00000011419 | NEK7 | UP | 4.84 | 0.0005 |
| ENSOCUT00000002665 | ENSOCUG00000002663 | GIGYF1 | DOWN | -4.41 | 0.0005 |
| ENSOCUT00000010094 | ENSOCUG00000010095 | ZNF18 | DOWN | -8.17 | 0.0006 |
| ENSOCUT00000005819 | ENSOCUG00000005819 | ZAP70 | DOWN | -4.14 | 0.0006 |
| ENSOCUT00000001553 | ENSOCUG00000001553 | LIPA | UP | 2.72 | 0.0006 |
| ENSOCUT00000014355 | ENSOCUG00000014338 | ABCA5 | UP | 6.02 | 0.0006 |
| ENSOCUT00000010968 | ENSOCUG00000010972 | LOC100328967 | DOWN | -9.73 | 0.0006 |
| ENSOCUT00000011844 | ENSOCUG00000011839 | SOX4 | DOWN | -9.98 | 0.0006 |
| ENSOCUT00000003796 | ENSOCUG00000003792 | THBS1 | UP | 3.39 | 0.0006 |
| ENSOCUT00000008208 | ENSOCUG00000008210 | CCR9 | DOWN | -8.79 | 0.0006 |
| ENSOCUT00000031413 | ENSOCUG00000005439 | CCNJL | DOWN | -4.25 | 0.0006 |
| ENSOCUT00000028795 | ENSOCUG00000022575 | VTN | UP | 6.01 | 0.0006 |
| ENSOCUT00000015008 | ENSOCUG00000015007 | SPNS3 | DOWN | -6.51 | 0.0006 |
| ENSOCUT00000015306 | ENSOCUG00000015300 | ACAP1 | DOWN | -3.92 | 0.0006 |
| ENSOCUT00000024258 | ENSOCUG00000021371 | . | DOWN | -6.23 | 0.0007 |
| ENSOCUT00000014678 | ENSOCUG00000014681 | RTRAF | UP | 3.18 | 0.0007 |
| ENSOCUT00000034047 | ENSOCUG00000029266 | LOC100352011 | DOWN | -8.78 | 0.0007 |
| ENSOCUT00000017271 | ENSOCUG00000017274 | CTLA4 | DOWN | -6.74 | 0.0007 |
| ENSOCUT00000033206 | ENSOCUG00000009212 | CD1A | DOWN | -10.63 | 0.0007 |
| ENSOCUT00000025240 | ENSOCUG00000025917 | . | DOWN | -6.18 | 0.0007 |
| ENSOCUT00000007824 | ENSOCUG00000007820 | ERAP1 | UP | 9.48 | 0.0008 |
| ENSOCUT00000003139 | ENSOCUG00000003140 | NFE2L3 | DOWN | -6.63 | 0.0008 |
| ENSOCUT00000002280 | ENSOCUG00000002281 | CYP2U1 | DOWN | -3.83 | 0.0008 |
| ENSOCUT00000024263 | ENSOCUG00000026103 | . | DOWN | -5.47 | 0.0008 |
| ENSOCUT00000006945 | ENSOCUG00000006945 | KRT8 | DOWN | -4.10 | 0.0009 |
| ENSOCUT00000004660 | ENSOCUG00000004662 | TCTEX1D1 | DOWN | -9.75 | 0.0009 |
| ENSOCUT00000009505 | ENSOCUG00000009508 | ADPRH | UP | 8.19 | 0.0009 |
| ENSOCUT00000012537 | ENSOCUG00000012534 | DSC1 | DOWN | -9.79 | 0.0009 |
| ENSOCUT00000013178 | ENSOCUG00000013176 | SMO | DOWN | -9.25 | 0.0010 |
| ENSOCUT00000033854 | ENSOCUG00000004451 | LIMS1 | DOWN | -3.51 | 0.0010 |
| ENSOCUT00000006456 | ENSOCUG00000006453 | NPR2 | DOWN | -9.44 | 0.0010 |
| ENSOCUT00000031843 | ENSOCUG00000021747 | WDR78 | DOWN | -3.78 | 0.0010 |
| ENSOCUT00000017942 | ENSOCUG00000024883 | LOC100354804 | UP | 4.02 | 0.0010 |
| ENSOCUT00000017017 | ENSOCUG00000017017 | CARNMT1 | DOWN | -4.53 | 0.0010 |
| ENSOCUT00000010705 | ENSOCUG00000010706 | FOSL2 | UP | 5.54 | 0.0010 |
| ENSOCUT00000013156 | ENSOCUG00000013160 | . | UP | 7.46 | 0.0011 |
| ENSOCUT00000031987 | ENSOCUG00000023316 | . | DOWN | -6.22 | 0.0011 |
| ENSOCUT00000015357 | ENSOCUG00000015320 | COL12A1 | UP | 3.39 | 0.0011 |
| ENSOCUT00000005697 | ENSOCUG00000005695 | PIGK | DOWN | -5.46 | 0.0011 |
| ENSOCUT00000016257 | ENSOCUG00000016254 | STAB2 | UP | 6.44 | 0.0012 |
| ENSOCUT00000005663 | ENSOCUG00000005666 | DHRS9 | UP | 4.28 | 0.0012 |
| ENSOCUT00000029021 | ENSOCUG00000025803 | LGALS9 | UP | 3.09 | 0.0012 |
| ENSOCUT00000008186 | ENSOCUG00000008191 | GABRP | DOWN | -6.16 | 0.0012 |
| ENSOCUT00000008213 | ENSOCUG00000008212 | DNTTIP2 | DOWN | -11.15 | 0.0012 |
| ENSOCUT00000029885 | ENSOCUG00000027812 | TUBA1A | UP | 7.43 | 0.0013 |
| ENSOCUT00000009383 | ENSOCUG00000009384 | CD8B | DOWN | -5.67 | 0.0013 |
| ENSOCUT00000010249 | ENSOCUG00000010238 | . | UP | 5.66 | 0.0013 |
| ENSOCUT00000007888 | ENSOCUG00000007892 | FBXL4 | UP | 5.55 | 0.0013 |
| ENSOCUT00000001104 | ENSOCUG00000001104 | RNF167 | UP | 4.55 | 0.0013 |
| ENSOCUT00000006060 | ENSOCUG00000006061 | THEMIS | DOWN | -5.02 | 0.0013 |
| ENSOCUT00000013134 | ENSOCUG00000013137 | CETP | UP | 4.61 | 0.0013 |
| ENSOCUT00000014002 | ENSOCUG00000013961 | DNAH11 | DOWN | -3.35 | 0.0013 |
| ENSOCUT00000001414 | ENSOCUG00000001414 | DDX23 | DOWN | -3.62 | 0.0014 |
| ENSOCUT00000025433 | ENSOCUG00000022557 | . | DOWN | -6.37 | 0.0014 |
| ENSOCUT00000016908 | ENSOCUG00000016906 | KCNQ5 | DOWN | -5.20 | 0.0014 |
| ENSOCUT00000028364 | ENSOCUG00000027167 | TRAV17 | DOWN | -6.41 | 0.0014 |
| ENSOCUT00000001754 | ENSOCUG00000001753 | ZFYVE16 | DOWN | -7.23 | 0.0014 |
| ENSOCUT00000033849 | ENSOCUG00000012136 | TBK1 | DOWN | -7.21 | 0.0015 |
| ENSOCUT00000003863 | ENSOCUG00000003863 | CD74 | DOWN | -10.96 | 0.0015 |
| ENSOCUT00000001483 | ENSOCUG00000001481 | RIPOR2 | DOWN | -6.47 | 0.0015 |
| ENSOCUT00000027573 | ENSOCUG00000025512 | SPINK8 | DOWN | -4.18 | 0.0016 |
| ENSOCUT00000029530 | ENSOCUG00000015654 | TRAK1 | DOWN | -10.86 | 0.0016 |
| ENSOCUT00000009701 | ENSOCUG00000009707 | PRELP | UP | 3.35 | 0.0016 |
| ENSOCUT00000007175 | ENSOCUG00000007174 | SATB1 | DOWN | -5.02 | 0.0016 |
| ENSOCUT00000006683 | ENSOCUG00000006684 | SRSF3 | UP | 3.64 | 0.0016 |
| ENSOCUT00000032998 | ENSOCUG00000029663 | KRT1 | DOWN | -9.32 | 0.0016 |
| ENSOCUT00000016318 | ENSOCUG00000016322 | IL12B | DOWN | -9.06 | 0.0016 |
| ENSOCUT00000026693 | ENSOCUG00000015616 | MOG | DOWN | -7.73 | 0.0016 |
| ENSOCUT00000022368 | ENSOCUG00000009241 | RAB11FIP2 | UP | 10.96 | 0.0017 |
| ENSOCUT00000023862 | ENSOCUG00000022391 | . | DOWN | -5.08 | 0.0017 |
| ENSOCUT00000015879 | ENSOCUG00000015884 | REG3G | DOWN | -8.14 | 0.0017 |
| ENSOCUT00000017683 | ENSOCUG00000017689 | KRT20 | DOWN | -8.85 | 0.0017 |
| ENSOCUT00000004925 | ENSOCUG00000004924 | NHLRC2 | DOWN | -4.87 | 0.0017 |
| ENSOCUT00000025179 | ENSOCUG00000021706 | . | DOWN | -6.67 | 0.0018 |
| ENSOCUT00000006973 | ENSOCUG00000006970 | SLC44A4 | UP | 3.44 | 0.0018 |
| ENSOCUT00000009341 | ENSOCUG00000009342 | THAP11 | DOWN | -8.52 | 0.0018 |
| ENSOCUT00000023334 | ENSOCUG00000025850 | IL2RG | DOWN | -10.78 | 0.0018 |
| ENSOCUT00000017182 | ENSOCUG00000017177 | WBP11 | UP | 5.02 | 0.0018 |
| ENSOCUT00000007336 | ENSOCUG00000007336 | TMX4 | UP | 8.65 | 0.0018 |
| ENSOCUT00000006348 | ENSOCUG00000006348 | PSMB10 | DOWN | -8.52 | 0.0018 |
| ENSOCUT00000025537 | ENSOCUG00000024484 | GGT6 | DOWN | -8.59 | 0.0018 |
| ENSOCUT00000033792 | ENSOCUG00000029541 | ZNF157 | DOWN | -7.50 | 0.0018 |
| ENSOCUT00000010433 | ENSOCUG00000010436 | LEF1 | DOWN | -4.07 | 0.0019 |
| ENSOCUT00000025285 | ENSOCUG00000024557 | . | DOWN | -9.05 | 0.0020 |
| ENSOCUT00000001591 | ENSOCUG00000001590 | KLC4 | UP | 7.47 | 0.0020 |
| ENSOCUT00000009193 | ENSOCUG00000009190 | HEXA | UP | 2.41 | 0.0021 |
| ENSOCUT00000021098 | ENSOCUG00000016696 | TTLL9 | UP | 8.44 | 0.0021 |
| ENSOCUT00000015956 | ENSOCUG00000015961 | PPP2CA | UP | 5.08 | 0.0021 |
| ENSOCUT00000006526 | ENSOCUG00000006526 | MPO | DOWN | -6.48 | 0.0021 |
| ENSOCUT00000004034 | ENSOCUG00000004035 | IRF6 | DOWN | -3.35 | 0.0022 |
| ENSOCUT00000022660 | ENSOCUG00000016406 | LOC100348076 | DOWN | -6.45 | 0.0022 |
| ENSOCUT00000011787 | ENSOCUG00000011780 | CYFIP2 | DOWN | -2.76 | 0.0022 |
| ENSOCUT00000003727 | ENSOCUG00000003724 | MEGF10 | UP | 10.66 | 0.0023 |
| ENSOCUT00000003297 | ENSOCUG00000003299 | DMRT2 | DOWN | -3.76 | 0.0023 |
| ENSOCUT00000011088 | ENSOCUG00000011093 | TRAV19 | DOWN | -4.90 | 0.0024 |
| ENSOCUT00000008560 | ENSOCUG00000008562 | BTD | DOWN | -3.67 | 0.0024 |
| ENSOCUT00000013826 | ENSOCUG00000013815 | SEMA6A | UP | 5.05 | 0.0024 |
| ENSOCUT00000025642 | ENSOCUG00000016980 | UTP6 | UP | 6.46 | 0.0024 |
| ENSOCUT00000016469 | ENSOCUG00000016465 | TP63 | DOWN | -5.19 | 0.0025 |
| ENSOCUT00000017336 | ENSOCUG00000017338 | EDIL3 | UP | 2.58 | 0.0025 |
| ENSOCUT00000003294 | ENSOCUG00000003294 | WDR26 | UP | 4.16 | 0.0025 |
| ENSOCUT00000013398 | ENSOCUG00000013367 | COL11A1 | UP | 3.17 | 0.0025 |
| ENSOCUT00000017669 | ENSOCUG00000017666 | TEK | UP | 10.57 | 0.0025 |
| ENSOCUT00000002557 | ENSOCUG00000002555 | SCP2 | UP | 9.52 | 0.0025 |
| ENSOCUT00000023527 | ENSOCUG00000022411 | GPR33 | DOWN | -8.28 | 0.0026 |
| ENSOCUT00000005262 | ENSOCUG00000005263 | TOX | DOWN | -3.35 | 0.0026 |
| ENSOCUT00000009864 | ENSOCUG00000009866 | ANKRD29 | UP | 3.35 | 0.0026 |
| ENSOCUT00000011795 | ENSOCUG00000011799 | FAM221A | DOWN | -3.10 | 0.0026 |
| ENSOCUT00000006187 | ENSOCUG00000006186 | CPXM1 | UP | 2.58 | 0.0026 |
| ENSOCUT00000024783 | ENSOCUG00000010887 | RPS6KA1 | UP | 3.07 | 0.0026 |
| ENSOCUT00000026926 | ENSOCUG00000020585 | HOXA9 | DOWN | -8.17 | 0.0027 |
| ENSOCUT00000013856 | ENSOCUG00000013839 | KIAA0100 | UP | 4.35 | 0.0028 |
| ENSOCUT00000000342 | ENSOCUG00000000342 | CEP126 | DOWN | -3.57 | 0.0028 |
| ENSOCUT00000025096 | ENSOCUG00000021120 | MUC15 | DOWN | -8.44 | 0.0028 |
| ENSOCUT00000016716 | ENSOCUG00000016712 | DNER | DOWN | -3.33 | 0.0029 |
| ENSOCUT00000009953 | ENSOCUG00000009952 | NDST3 | DOWN | -5.16 | 0.0029 |
| ENSOCUT00000000384 | ENSOCUG00000000386 | RCAN3 | UP | 4.41 | 0.0029 |
| ENSOCUT00000000574 | ENSOCUG00000000575 | HAVCR1 | UP | 2.72 | 0.0029 |
| ENSOCUT00000006324 | ENSOCUG00000006325 | GALM | UP | 2.45 | 0.0030 |
| ENSOCUT00000016368 | ENSOCUG00000016368 | TMEM14C | UP | 8.30 | 0.0030 |
| ENSOCUT00000010793 | ENSOCUG00000010796 | RS1 | UP | 4.71 | 0.0030 |
| ENSOCUT00000023209 | ENSOCUG00000026533 | CCL17 | DOWN | -5.02 | 0.0030 |
| ENSOCUT00000013076 | ENSOCUG00000013078 | FAXDC2 | UP | 3.65 | 0.0031 |
| ENSOCUT00000029094 | ENSOCUG00000025844 | HINT3 | UP | 5.17 | 0.0031 |
| ENSOCUT00000006805 | ENSOCUG00000006797 | MED12 | UP | 6.83 | 0.0031 |
| ENSOCUT00000009756 | ENSOCUG00000009751 | HCK | UP | 2.50 | 0.0032 |
| ENSOCUT00000001858 | ENSOCUG00000001858 | ICOS | DOWN | -3.77 | 0.0032 |
| ENSOCUT00000003822 | ENSOCUG00000003822 | FAM83B | DOWN | -6.16 | 0.0032 |
| ENSOCUT00000005172 | ENSOCUG00000005174 | DPT | DOWN | -5.36 | 0.0032 |
| ENSOCUT00000008146 | ENSOCUG00000008142 | TCF7L2 | DOWN | -10.29 | 0.0032 |
| ENSOCUT00000016004 | ENSOCUG00000016005 | EXO5 | DOWN | -4.93 | 0.0032 |
| ENSOCUT00000009326 | ENSOCUG00000009322 | MCOLN2 | UP | 2.75 | 0.0033 |
| ENSOCUT00000004741 | ENSOCUG00000004738 | STK31 | DOWN | -4.50 | 0.0033 |
| ENSOCUT00000033266 | ENSOCUG00000015651 | ATG3 | DOWN | -4.65 | 0.0033 |
| ENSOCUT00000022665 | ENSOCUG00000026966 | SASH3 | DOWN | -3.18 | 0.0034 |
| ENSOCUT00000009923 | ENSOCUG00000009929 | C14H3orf52 | DOWN | -5.54 | 0.0034 |
| ENSOCUT00000012880 | ENSOCUG00000012872 | . | DOWN | -6.57 | 0.0034 |
| ENSOCUT00000025052 | ENSOCUG00000026232 | . | DOWN | -7.88 | 0.0035 |
| ENSOCUT00000024322 | ENSOCUG00000024349 | LOC100337909 | UP | 2.99 | 0.0035 |
| ENSOCUT00000015721 | ENSOCUG00000015723 | SLC15A3 | UP | 2.30 | 0.0035 |
| ENSOCUT00000003057 | ENSOCUG00000003059 | CTBS | UP | 2.28 | 0.0036 |
| ENSOCUT00000013890 | ENSOCUG00000013891 | LOC108175363 | DOWN | -2.43 | 0.0036 |
| ENSOCUT00000033945 | ENSOCUG00000002595 | ANKRD33B | DOWN | -3.76 | 0.0037 |
| ENSOCUT00000033640 | ENSOCUG00000029253 | LOC100346726 | DOWN | -5.82 | 0.0037 |
| ENSOCUT00000006988 | ENSOCUG00000006986 | SCIN | DOWN | -3.15 | 0.0037 |
| ENSOCUT00000012111 | ENSOCUG00000012103 | FAT2 | DOWN | -3.90 | 0.0037 |
| ENSOCUT00000033793 | ENSOCUG00000029708 | . | DOWN | -7.86 | 0.0037 |
| ENSOCUT00000013287 | ENSOCUG00000013285 | KAT2A | UP | 10.25 | 0.0038 |
| ENSOCUT00000002256 | ENSOCUG00000002254 | DNMBP | UP | 4.14 | 0.0038 |
| ENSOCUT00000011360 | ENSOCUG00000011359 | ACAD10 | DOWN | -10.17 | 0.0038 |
| ENSOCUT00000032195 | ENSOCUG00000026467 | TRAV8-7 | DOWN | -6.04 | 0.0038 |
| ENSOCUT00000017287 | ENSOCUG00000017290 | . | DOWN | -7.79 | 0.0039 |
| ENSOCUT00000007275 | ENSOCUG00000007276 | MTPAP | UP | 8.45 | 0.0039 |
| ENSOCUT00000025664 | ENSOCUG00000012769 | TSC22D1 | DOWN | -10.15 | 0.0039 |
| ENSOCUT00000006354 | ENSOCUG00000006350 | SLC12A4 | UP | 5.82 | 0.0039 |
| ENSOCUT00000026720 | ENSOCUG00000016883 | FERMT1 | DOWN | -5.84 | 0.0039 |
| ENSOCUT00000028395 | ENSOCUG00000014171 | MKRN2 | UP | 3.60 | 0.0039 |
| ENSOCUT00000030548 | ENSOCUG00000024319 | LOC103351908 | DOWN | -4.06 | 0.0040 |
| ENSOCUT00000011739 | ENSOCUG00000011739 | RUNX2 | DOWN | -7.02 | 0.0040 |
| ENSOCUT00000031937 | ENSOCUG00000027241 | MFAP3 | UP | 4.27 | 0.0040 |
| ENSOCUT00000033386 | ENSOCUG00000021242 | PKP1 | DOWN | -3.81 | 0.0040 |
| ENSOCUT00000017019 | ENSOCUG00000017019 | CDH11 | UP | 2.46 | 0.0040 |
| ENSOCUT00000006370 | ENSOCUG00000006372 | KLHL28 | DOWN | -4.66 | 0.0040 |
| ENSOCUT00000015027 | ENSOCUG00000015029 | HNRNPC | UP | 3.78 | 0.0040 |
| ENSOCUT00000013596 | ENSOCUG00000013593 | BPI | DOWN | -4.33 | 0.0040 |
| ENSOCUT00000015493 | ENSOCUG00000015484 | CRNKL1 | DOWN | -10.02 | 0.0042 |
| ENSOCUT00000010587 | ENSOCUG00000010589 | FBXO30 | DOWN | -10.07 | 0.0043 |
| ENSOCUT00000011266 | ENSOCUG00000011267 | ARHGEF9 | UP | 2.81 | 0.0044 |
| ENSOCUT00000022998 | ENSOCUG00000022960 | S100G | DOWN | -8.02 | 0.0044 |
| ENSOCUT00000006804 | ENSOCUG00000006800 | ATP10B | DOWN | -4.25 | 0.0044 |
| ENSOCUT00000028233 | ENSOCUG00000024654 | IL37 | DOWN | -8.21 | 0.0044 |
| ENSOCUT00000015520 | ENSOCUG00000015523 | JHY | DOWN | -5.00 | 0.0045 |
| ENSOCUT00000024311 | ENSOCUG00000008575 | LOC100125981 | UP | 2.40 | 0.0045 |
| ENSOCUT00000005899 | ENSOCUG00000005896 | INPP4B | DOWN | -3.45 | 0.0045 |
| ENSOCUT00000004260 | ENSOCUG00000004246 | PKHD1 | DOWN | -4.43 | 0.0046 |
| ENSOCUT00000014208 | ENSOCUG00000014205 | AKAP6 | UP | 5.33 | 0.0046 |
| ENSOCUT00000013566 | ENSOCUG00000013567 | GRN | UP | 3.12 | 0.0047 |
| ENSOCUT00000025524 | ENSOCUG00000021858 | RIOX1 | UP | 10.07 | 0.0048 |
| ENSOCUT00000003316 | ENSOCUG00000003319 | HLA-DPA1 | UP | 10.06 | 0.0048 |
| ENSOCUT00000006953 | ENSOCUG00000006954 | PRF1 | DOWN | -3.53 | 0.0048 |
| ENSOCUT00000012892 | ENSOCUG00000012881 | COL1A1 | UP | 4.24 | 0.0050 |
| ENSOCUT00000001951 | ENSOCUG00000001949 | MRPL15 | DOWN | -4.37 | 0.0051 |
| ENSOCUT00000003102 | ENSOCUG00000003103 | CXADR | DOWN | -4.94 | 0.0051 |
| ENSOCUT00000022202 | ENSOCUG00000022536 | SPP1 | UP | 2.75 | 0.0054 |
| ENSOCUT00000002989 | ENSOCUG00000002991 | CLCC1 | UP | 4.27 | 0.0056 |
| ENSOCUT00000026874 | ENSOCUG00000017274 | CTLA4 | DOWN | -5.12 | 0.0056 |
| ENSOCUT00000002273 | ENSOCUG00000002274 | BCL6B | UP | 4.16 | 0.0057 |
| ENSOCUT00000008473 | ENSOCUG00000008464 | PRKG2 | DOWN | -3.38 | 0.0057 |
| ENSOCUT00000010545 | ENSOCUG00000010536 | RALGAPB | DOWN | -9.58 | 0.0057 |
| ENSOCUT00000020923 | ENSOCUG00000017519 | MYO10 | UP | 2.12 | 0.0057 |
| ENSOCUT00000028961 | ENSOCUG00000024789 | LPAR3 | DOWN | -5.59 | 0.0057 |
| ENSOCUT00000029432 | ENSOCUG00000026630 | ITGA9 | UP | 5.04 | 0.0058 |
| ENSOCUT00000004664 | ENSOCUG00000004666 | INSL5 | DOWN | -7.36 | 0.0058 |
| ENSOCUT00000017679 | ENSOCUG00000017680 | FAM212B | DOWN | -4.94 | 0.0058 |
| ENSOCUT00000012643 | ENSOCUG00000012653 | . | UP | 8.45 | 0.0058 |
| ENSOCUT00000011873 | ENSOCUG00000011872 | . | UP | 9.77 | 0.0058 |
| ENSOCUT00000002339 | ENSOCUG00000002341 | CAPG | UP | 3.52 | 0.0062 |
| ENSOCUT00000008576 | ENSOCUG00000008568 | ANKRD28 | UP | 2.58 | 0.0063 |
| ENSOCUT00000024355 | ENSOCUG00000025830 | CAPN5 | DOWN | -7.81 | 0.0063 |
| ENSOCUT00000033298 | ENSOCUG00000013567 | GRN | UP | 2.87 | 0.0065 |
| ENSOCUT00000001005 | ENSOCUG00000001006 | TACR1 | DOWN | -2.90 | 0.0066 |
| ENSOCUT00000003024 | ENSOCUG00000003025 | NARS | DOWN | -4.19 | 0.0067 |
| ENSOCUT00000006236 | ENSOCUG00000006227 | JAK1 | UP | 2.74 | 0.0067 |
| ENSOCUT00000014528 | ENSOCUG00000014527 | CTSC | UP | 2.14 | 0.0067 |
| ENSOCUT00000033448 | ENSOCUG00000029471 | . | DOWN | -6.44 | 0.0068 |
| ENSOCUT00000031823 | ENSOCUG00000014718 | NOS1 | DOWN | -5.01 | 0.0068 |
| ENSOCUT00000003090 | ENSOCUG00000003089 | PRG3 | DOWN | -5.45 | 0.0069 |
| ENSOCUT00000029633 | ENSOCUG00000004710 | DDX58 | DOWN | -4.75 | 0.0070 |
| ENSOCUT00000014962 | ENSOCUG00000014963 | . | DOWN | -4.78 | 0.0071 |
| ENSOCUT00000015613 | ENSOCUG00000015612 | NOL11 | DOWN | -3.15 | 0.0071 |
| ENSOCUT00000001106 | ENSOCUG00000001106 | PFN1 | UP | 4.80 | 0.0071 |
| ENSOCUT00000010200 | ENSOCUG00000010200 | PMPCB | DOWN | -4.27 | 0.0073 |
| ENSOCUT00000033822 | ENSOCUG00000003527 | USP7 | UP | 9.34 | 0.0075 |
| ENSOCUT00000029173 | ENSOCUG00000014425 | FILIP1L | DOWN | -6.73 | 0.0076 |
| ENSOCUT00000015664 | ENSOCUG00000015667 | SH3RF2 | DOWN | -3.28 | 0.0076 |
| ENSOCUT00000030615 | ENSOCUG00000022769 | . | UP | 9.31 | 0.0077 |
| ENSOCUT00000014815 | ENSOCUG00000014811 | KIAA1109 | DOWN | -3.03 | 0.0077 |
| ENSOCUT00000009046 | ENSOCUG00000009047 | FCRL2 | UP | 3.14 | 0.0078 |
| ENSOCUT00000028655 | ENSOCUG00000021131 | SMUG1 | UP | 9.28 | 0.0079 |
| ENSOCUT00000004240 | ENSOCUG00000004244 | KLF12 | DOWN | -9.14 | 0.0079 |
| ENSOCUT00000025296 | ENSOCUG00000014689 | IGF1 | DOWN | -3.96 | 0.0080 |
| ENSOCUT00000008235 | ENSOCUG00000008236 | LPL | DOWN | -2.50 | 0.0080 |
| ENSOCUT00000031171 | ENSOCUG00000009547 | MYO5B | DOWN | -3.37 | 0.0080 |
| ENSOCUT00000001316 | ENSOCUG00000001316 | ESRP1 | DOWN | -5.66 | 0.0080 |
| ENSOCUT00000004498 | ENSOCUG00000004494 | THSD7B | DOWN | -4.34 | 0.0081 |
| ENSOCUT00000014334 | ENSOCUG00000014332 | SHF | DOWN | -9.04 | 0.0081 |
| ENSOCUT00000034032 | ENSOCUG00000012754 | RUBCN | DOWN | -3.79 | 0.0081 |
| ENSOCUT00000017499 | ENSOCUG00000017499 | PLPPR5 | DOWN | -2.91 | 0.0081 |
| ENSOCUT00000017698 | ENSOCUG00000017698 | GCM2 | UP | 7.62 | 0.0082 |
| ENSOCUT00000006029 | ENSOCUG00000006031 | TLR6 | UP | 9.22 | 0.0082 |
| ENSOCUT00000016830 | ENSOCUG00000016831 | SLC27A2 | DOWN | -4.58 | 0.0083 |
| ENSOCUT00000033516 | ENSOCUG00000029196 | LOC100353028 | DOWN | -3.52 | 0.0083 |
| ENSOCUT00000002248 | ENSOCUG00000002249 | DHRS7 | UP | 2.20 | 0.0084 |
| ENSOCUT00000021509 | ENSOCUG00000023515 | . | DOWN | -5.06 | 0.0085 |
| ENSOCUT00000005881 | ENSOCUG00000005883 | GPR87 | DOWN | -4.88 | 0.0086 |
| ENSOCUT00000027669 | ENSOCUG00000023049 | . | DOWN | -4.71 | 0.0087 |
| ENSOCUT00000010672 | ENSOCUG00000010671 | SH2D2A | DOWN | -3.28 | 0.0087 |
| ENSOCUT00000026374 | ENSOCUG00000010971 | CRYGB | DOWN | -6.49 | 0.0087 |
| ENSOCUT00000017785 | ENSOCUG00000017786 | TBATA | DOWN | -6.17 | 0.0088 |
| ENSOCUT00000005729 | ENSOCUG00000005729 | RIN3 | UP | 2.41 | 0.0089 |
| ENSOCUT00000026230 | ENSOCUG00000026480 | AOX2 | DOWN | -3.97 | 0.0089 |
| ENSOCUT00000022037 | ENSOCUG00000006320 | SMARCA2 | UP | 3.93 | 0.0089 |
| ENSOCUT00000006752 | ENSOCUG00000006752 | MEST | UP | 2.59 | 0.0090 |
| ENSOCUT00000028972 | ENSOCUG00000024081 | CD63 | UP | 2.67 | 0.0090 |
| ENSOCUT00000000578 | ENSOCUG00000000574 | PLEKHH1 | UP | 2.63 | 0.0091 |
| ENSOCUT00000022103 | ENSOCUG00000026636 | MYH1 | UP | 5.43 | 0.0091 |
| ENSOCUT00000014111 | ENSOCUG00000014109 | CDCA2 | DOWN | -4.88 | 0.0091 |
| ENSOCUT00000023326 | ENSOCUG00000024102 | . | DOWN | -4.67 | 0.0091 |
| ENSOCUT00000009769 | ENSOCUG00000009764 | GPD2 | DOWN | -8.84 | 0.0091 |
| ENSOCUT00000033681 | ENSOCUG00000000776 | LEMD3 | DOWN | -8.87 | 0.0092 |
| ENSOCUT00000016824 | ENSOCUG00000016821 | MCRS1 | DOWN | -8.82 | 0.0092 |
| ENSOCUT00000022344 | ENSOCUG00000026003 | . | UP | 2.68 | 0.0093 |
| ENSOCUT00000022030 | ENSOCUG00000021209 | LOC100343557 | UP | 3.32 | 0.0093 |
| ENSOCUT00000008207 | ENSOCUG00000008204 | SOAT1 | UP | 2.09 | 0.0093 |
| ENSOCUT00000010189 | ENSOCUG00000010189 | LEP | DOWN | -3.76 | 0.0093 |
| ENSOCUT00000031147 | ENSOCUG00000022470 | DRG1 | UP | 2.77 | 0.0094 |
| ENSOCUT00000004006 | ENSOCUG00000003999 | CAMK1G | DOWN | -4.35 | 0.0094 |
| ENSOCUT00000017601 | ENSOCUG00000006566 | CYBB | UP | 4.40 | 0.0094 |
| ENSOCUT00000001355 | ENSOCUG00000001355 | IKZF3 | DOWN | -3.55 | 0.0095 |
| ENSOCUT00000002766 | ENSOCUG00000002770 | FAM3D | DOWN | -6.96 | 0.0097 |
| ENSOCUT00000029634 | ENSOCUG00000009645 | SAT1 | UP | 2.04 | 0.0098 |
| ENSOCUT00000006232 | ENSOCUG00000006229 | TYRO3 | UP | 8.88 | 0.0098 |
| ENSOCUT00000016656 | ENSOCUG00000016650 | DEPDC1B | DOWN | -3.39 | 0.0098 |
| ENSOCUT00000000572 | ENSOCUG00000000572 | FAM192A | DOWN | -3.97 | 0.0100 |
| ENSOCUT00000012461 | ENSOCUG00000012457 | GOLGB1 | DOWN | -2.18 | 0.0100 |
| ENSOCUT00000006034 | ENSOCUG00000029127 | CD164 | UP | 2.78 | 0.0101 |
| ENSOCUT00000004614 | ENSOCUG00000004617 | ANKRD22 | DOWN | -4.44 | 0.0101 |
| ENSOCUT00000034167 | ENSOCUG00000011298 | FAM83D | DOWN | -2.79 | 0.0103 |
| ENSOCUT00000012464 | ENSOCUG00000012464 | CD6 | DOWN | -3.19 | 0.0103 |
| ENSOCUT00000003287 | ENSOCUG00000000547 | SGO2 | DOWN | -2.93 | 0.0104 |
| ENSOCUT00000002691 | ENSOCUG00000002694 | KPNA1 | UP | 8.74 | 0.0105 |
| ENSOCUT00000007803 | ENSOCUG00000007807 | ATMIN | UP | 3.52 | 0.0105 |
| ENSOCUT00000033746 | ENSOCUG00000029433 | TRAV24 | DOWN | -5.24 | 0.0106 |
| ENSOCUT00000002916 | ENSOCUG00000002916 | TRAT1 | DOWN | -4.10 | 0.0106 |
| ENSOCUT00000021363 | ENSOCUG00000022135 | NME1 | DOWN | -3.78 | 0.0106 |
| ENSOCUT00000000113 | ENSOCUG00000000114 | DUSP2 | DOWN | -2.81 | 0.0108 |
| ENSOCUT00000033410 | ENSOCUG00000029742 | . | UP | 4.92 | 0.0108 |
| ENSOCUT00000000073 | ENSOCUG00000000073 | INPP5K | UP | 4.67 | 0.0110 |
| ENSOCUT00000001386 | ENSOCUG00000001387 | TDRKH | DOWN | -3.51 | 0.0110 |
| ENSOCUT00000014014 | ENSOCUG00000014003 | BUB1 | DOWN | -3.08 | 0.0110 |
| ENSOCUT00000008337 | ENSOCUG00000008329 | ADAMTS19 | DOWN | -6.60 | 0.0115 |
| ENSOCUT00000024440 | ENSOCUG00000016744 | SPOCK2 | DOWN | -8.38 | 0.0116 |
| ENSOCUT00000026506 | ENSOCUG00000017148 | RYK | UP | 3.04 | 0.0118 |
| ENSOCUT00000034146 | ENSOCUG00000009866 | ANKRD29 | UP | 6.02 | 0.0119 |
| ENSOCUT00000027317 | ENSOCUG00000024677 | LOC100358336 | UP | 2.46 | 0.0120 |
| ENSOCUT00000009457 | ENSOCUG00000009455 | FRRS1 | UP | 2.48 | 0.0121 |
| ENSOCUT00000008771 | ENSOCUG00000008771 | IL1A | UP | 4.51 | 0.0121 |
| ENSOCUT00000031006 | ENSOCUG00000027298 | . | DOWN | -5.25 | 0.0121 |
| ENSOCUT00000016710 | ENSOCUG00000016706 | KIF11 | DOWN | -2.90 | 0.0123 |
| ENSOCUT00000003208 | ENSOCUG00000003209 | CCBE1 | UP | 6.04 | 0.0125 |
| ENSOCUT00000011704 | ENSOCUG00000011704 | . | UP | 3.83 | 0.0126 |
| ENSOCUT00000009166 | ENSOCUG00000009165 | TTI1 | UP | 3.30 | 0.0126 |
| ENSOCUT00000014395 | ENSOCUG00000014398 | RAP2B | UP | 5.49 | 0.0127 |
| ENSOCUT00000015209 | ENSOCUG00000015166 | . | UP | 5.97 | 0.0128 |
| ENSOCUT00000012975 | ENSOCUG00000012977 | C11orf52 | DOWN | -6.54 | 0.0131 |
| ENSOCUT00000024708 | ENSOCUG00000022021 | IL22RA2 | DOWN | -5.20 | 0.0132 |
| ENSOCUT00000005289 | ENSOCUG00000005292 | TRIM2 | UP | 4.62 | 0.0133 |
| ENSOCUT00000008320 | ENSOCUG00000023743 | . | DOWN | -2.77 | 0.0133 |
| ENSOCUT00000030935 | ENSOCUG00000025569 | CCR7 | DOWN | -2.84 | 0.0134 |
| ENSOCUT00000031649 | ENSOCUG00000027537 | . | DOWN | -4.43 | 0.0135 |
| ENSOCUT00000003898 | ENSOCUG00000003897 | PRIMPOL | DOWN | -2.86 | 0.0136 |
| ENSOCUT00000002106 | ENSOCUG00000002106 | TDP1 | DOWN | -5.62 | 0.0137 |
| ENSOCUT00000002623 | ENSOCUG00000002623 | ESAM | UP | 2.67 | 0.0138 |
| ENSOCUT00000028494 | ENSOCUG00000021297 | TRIM29 | DOWN | -8.09 | 0.0138 |
| ENSOCUT00000001542 | ENSOCUG00000001542 | RIPK2 | UP | 2.20 | 0.0139 |
| ENSOCUT00000023673 | ENSOCUG00000022253 | . | DOWN | -3.99 | 0.0139 |
| ENSOCUT00000002233 | ENSOCUG00000002231 | VLDLR | UP | 2.47 | 0.0139 |
| ENSOCUT00000006547 | ENSOCUG00000006540 | PLCB2 | UP | 2.07 | 0.0139 |
| ENSOCUT00000009076 | ENSOCUG00000009077 | MS4A2 | DOWN | -5.64 | 0.0140 |
| ENSOCUT00000017230 | ENSOCUG00000017165 | LRP1B | UP | 3.07 | 0.0141 |
| ENSOCUT00000028918 | ENSOCUG00000016432 | OLFM4 | DOWN | -5.89 | 0.0142 |
| ENSOCUT00000031997 | ENSOCUG00000022883 | GDPD3 | DOWN | -4.70 | 0.0142 |
| ENSOCUT00000025439 | ENSOCUG00000021388 | P2RX1 | DOWN | -3.02 | 0.0142 |
| ENSOCUT00000004173 | ENSOCUG00000004169 | ASAH1 | UP | 1.93 | 0.0143 |
| ENSOCUT00000025996 | ENSOCUG00000028146 | . | DOWN | -6.55 | 0.0143 |
| ENSOCUT00000013561 | ENSOCUG00000013564 | TRIM63 | DOWN | -2.61 | 0.0144 |
| ENSOCUT00000010262 | ENSOCUG00000010261 | EHF | DOWN | -3.14 | 0.0144 |
| ENSOCUT00000004804 | ENSOCUG00000004804 | FLVCR1 | DOWN | -3.09 | 0.0145 |
| ENSOCUT00000015033 | ENSOCUG00000015020 | COL5A2 | UP | 2.06 | 0.0146 |
| ENSOCUT00000014935 | ENSOCUG00000026994 | TUBA4A | DOWN | -2.40 | 0.0146 |
| ENSOCUT00000004171 | ENSOCUG00000004172 | VSIG4 | UP | 5.71 | 0.0151 |
| ENSOCUT00000006795 | ENSOCUG00000006792 | NCAPG | DOWN | -2.66 | 0.0153 |
| ENSOCUT00000009760 | ENSOCUG00000009757 | SEPT-4 | UP | 3.68 | 0.0153 |
| ENSOCUT00000015133 | ENSOCUG00000015138 | C1QC | UP | 2.03 | 0.0155 |
| ENSOCUT00000000111 | ENSOCUG00000000109 | ADAMTS9 | DOWN | -2.87 | 0.0156 |
| ENSOCUT00000029437 | ENSOCUG00000003276 | PAX1 | DOWN | -4.37 | 0.0158 |
| ENSOCUT00000006898 | ENSOCUG00000006899 | KIF18B | DOWN | -3.30 | 0.0158 |
| ENSOCUT00000010553 | ENSOCUG00000010551 | SLC7A3 | DOWN | -2.88 | 0.0160 |
| ENSOCUT00000006462 | ENSOCUG00000006463 | PRKAG2 | DOWN | -4.03 | 0.0161 |
| ENSOCUT00000028015 | ENSOCUG00000023681 | TSPAN18 | UP | 3.57 | 0.0161 |
| ENSOCUT00000011546 | ENSOCUG00000011550 | CD86 | UP | 2.05 | 0.0161 |
| ENSOCUT00000027500 | ENSOCUG00000023836 | NEFL | DOWN | -2.67 | 0.0162 |
| ENSOCUT00000005427 | ENSOCUG00000005428 | ALOX5 | DOWN | -2.63 | 0.0163 |
| ENSOCUT00000015530 | ENSOCUG00000015533 | HSPA8 | DOWN | -1.98 | 0.0165 |
| ENSOCUT00000022147 | ENSOCUG00000025121 | ALDH6A1 | DOWN | -3.86 | 0.0165 |
| ENSOCUT00000026954 | ENSOCUG00000026443 | UNC45B | DOWN | -4.02 | 0.0166 |
| ENSOCUT00000014811 | ENSOCUG00000014812 | CD93 | UP | 2.14 | 0.0166 |
| ENSOCUT00000009408 | ENSOCUG00000009402 | USP32 | DOWN | -2.17 | 0.0167 |
| ENSOCUT00000008157 | ENSOCUG00000008159 | CD27 | DOWN | -4.79 | 0.0168 |
| ENSOCUT00000009183 | ENSOCUG00000009184 | CTHRC1 | UP | 2.78 | 0.0168 |
| ENSOCUT00000024150 | ENSOCUG00000023078 | . | UP | 2.79 | 0.0169 |
| ENSOCUT00000004356 | ENSOCUG00000004358 | CACYBP | DOWN | -4.17 | 0.0169 |
| ENSOCUT00000033850 | ENSOCUG00000004627 | APH1B | UP | 1.93 | 0.0170 |
| ENSOCUT00000014994 | ENSOCUG00000014996 | SH3RF3 | DOWN | -2.76 | 0.0170 |
| ENSOCUT00000016964 | ENSOCUG00000016959 | CRYBG2 | DOWN | -3.42 | 0.0171 |
| ENSOCUT00000009963 | ENSOCUG00000009965 | TYR | DOWN | -3.33 | 0.0172 |
| ENSOCUT00000012672 | ENSOCUG00000012675 | GFRA4 | DOWN | -5.43 | 0.0173 |
| ENSOCUT00000012565 | ENSOCUG00000012561 | SLC43A3 | UP | 2.51 | 0.0173 |
| ENSOCUT00000012518 | ENSOCUG00000012518 | DSC2 | DOWN | -5.11 | 0.0174 |
| ENSOCUT00000007500 | ENSOCUG00000007499 | LMNB1 | DOWN | -2.33 | 0.0174 |
| ENSOCUT00000003911 | ENSOCUG00000003911 | CD40LG | DOWN | -3.13 | 0.0175 |
| ENSOCUT00000033614 | ENSOCUG00000000285 | LBR | DOWN | -3.35 | 0.0175 |
| ENSOCUT00000016828 | ENSOCUG00000016828 | PRR14L | DOWN | -2.63 | 0.0175 |
| ENSOCUT00000029300 | ENSOCUG00000023401 | ADH2-2 | DOWN | -2.92 | 0.0176 |
| ENSOCUT00000002427 | ENSOCUG00000002427 | SLC6A17 | DOWN | -2.70 | 0.0177 |
| ENSOCUT00000017269 | ENSOCUG00000017266 | FAM168B | DOWN | -4.38 | 0.0178 |
| ENSOCUT00000008954 | ENSOCUG00000008953 | SYNPO2 | UP | 3.15 | 0.0178 |
| ENSOCUT00000004293 | ENSOCUG00000004292 | STRADA | DOWN | -3.17 | 0.0178 |
| ENSOCUT00000009930 | ENSOCUG00000009932 | ESCO2 | DOWN | -2.67 | 0.0179 |
| ENSOCUT00000025440 | ENSOCUG00000020939 | . | UP | 4.49 | 0.0179 |
| ENSOCUT00000002266 | ENSOCUG00000015482 | TMEM241 | DOWN | -4.15 | 0.0179 |
| ENSOCUT00000003569 | ENSOCUG00000003569 | HMGCS1 | UP | 5.17 | 0.0179 |
| ENSOCUT00000008465 | ENSOCUG00000008470 | DCTN5 | DOWN | -4.31 | 0.0182 |
| ENSOCUT00000033656 | ENSOCUG00000029216 | C17H15orf48 | UP | 2.57 | 0.0183 |
| ENSOCUT00000007335 | ENSOCUG00000007335 | HS3ST2 | DOWN | -6.20 | 0.0184 |
| ENSOCUT00000008407 | ENSOCUG00000008408 | ATF7IP2 | DOWN | -3.09 | 0.0184 |
| ENSOCUT00000027133 | ENSOCUG00000027818 | . | DOWN | -5.55 | 0.0185 |
| ENSOCUT00000033897 | ENSOCUG00000006800 | ATP10B | DOWN | -2.57 | 0.0185 |
| ENSOCUT00000011025 | ENSOCUG00000011027 | CHIT1 | UP | 3.91 | 0.0188 |
| ENSOCUT00000031168 | ENSOCUG00000006703 | LTB | DOWN | -4.73 | 0.0189 |
| ENSOCUT00000026174 | ENSOCUG00000022573 | HLA-DMA | UP | 2.03 | 0.0189 |
| ENSOCUT00000000061 | ENSOCUG00000000061 | FBXO5 | DOWN | -2.60 | 0.0189 |
| ENSOCUT00000029756 | ENSOCUG00000022216 | COLEC12 | DOWN | -6.54 | 0.0189 |
| ENSOCUT00000013457 | ENSOCUG00000013454 | PRDM5 | UP | 4.16 | 0.0190 |
| ENSOCUT00000004634 | ENSOCUG00000004632 | CA12 | UP | 2.82 | 0.0192 |
| ENSOCUT00000008618 | ENSOCUG00000008620 | DAPL1 | DOWN | -4.07 | 0.0192 |
| ENSOCUT00000011078 | ENSOCUG00000011080 | GALNT7 | DOWN | -2.26 | 0.0192 |
| ENSOCUT00000033075 | ENSOCUG00000029068 | ZNF383 | DOWN | -4.03 | 0.0192 |
| ENSOCUT00000000931 | ENSOCUG00000000930 | KIF15 | DOWN | -2.38 | 0.0193 |
| ENSOCUT00000009577 | ENSOCUG00000009576 | MSH2 | UP | 4.71 | 0.0194 |
| ENSOCUT00000011281 | ENSOCUG00000011283 | NUDT4 | DOWN | -4.14 | 0.0196 |
| ENSOCUT00000013495 | ENSOCUG00000013498 | ADAMTS4 | UP | 2.98 | 0.0196 |
| ENSOCUT00000013501 | ENSOCUG00000013505 | CPSF6 | DOWN | -1.93 | 0.0197 |
| ENSOCUT00000007793 | ENSOCUG00000007795 | SERPINB10 | DOWN | -3.04 | 0.0198 |
| ENSOCUT00000008706 | ENSOCUG00000008707 | RTL5 | UP | 7.57 | 0.0198 |
| ENSOCUT00000033818 | ENSOCUG00000001414 | DDX23 | UP | 3.02 | 0.0200 |
| ENSOCUT00000027245 | ENSOCUG00000006085 | BBOX1 | DOWN | -2.83 | 0.0200 |
| ENSOCUT00000013627 | ENSOCUG00000013625 | PRSS12 | UP | 2.19 | 0.0201 |
| ENSOCUT00000011013 | ENSOCUG00000011016 | . | DOWN | -7.44 | 0.0202 |
| ENSOCUT00000002941 | ENSOCUG00000002945 | MEOX1 | UP | 2.33 | 0.0203 |
| ENSOCUT00000005707 | ENSOCUG00000005708 | STC1 | UP | 2.46 | 0.0203 |
| ENSOCUT00000014124 | ENSOCUG00000014125 | PGM5 | DOWN | -1.96 | 0.0204 |
| ENSOCUT00000009502 | ENSOCUG00000009504 | CD80 | UP | 1.95 | 0.0205 |
| ENSOCUT00000006801 | ENSOCUG00000006801 | MCM10 | DOWN | -2.87 | 0.0205 |
| ENSOCUT00000013713 | ENSOCUG00000013715 | C5orf34 | DOWN | -4.04 | 0.0206 |
| ENSOCUT00000016071 | ENSOCUG00000016073 | OSTM1 | UP | 1.75 | 0.0213 |
| ENSOCUT00000008087 | ENSOCUG00000008077 | POLQ | DOWN | -2.62 | 0.0214 |
| ENSOCUT00000028390 | ENSOCUG00000024358 | CDIPT | DOWN | -2.31 | 0.0215 |
| ENSOCUT00000006381 | ENSOCUG00000006381 | LY86 | UP | 2.33 | 0.0216 |
| ENSOCUT00000023444 | ENSOCUG00000011064 | CLSTN1 | DOWN | -3.61 | 0.0217 |
| ENSOCUT00000014839 | ENSOCUG00000014835 | SMC4 | DOWN | -2.93 | 0.0219 |
| ENSOCUT00000029378 | ENSOCUG00000029288 | . | DOWN | -5.38 | 0.0219 |
| ENSOCUT00000001819 | ENSOCUG00000001819 | ANAPC10 | DOWN | -3.40 | 0.0221 |
| ENSOCUT00000033372 | ENSOCUG00000004337 | SMARCD2 | DOWN | -2.36 | 0.0222 |
| ENSOCUT00000014225 | ENSOCUG00000014225 | SLCO2B1 | UP | 1.89 | 0.0223 |
| ENSOCUT00000009063 | ENSOCUG00000009065 | HNMT | UP | 1.96 | 0.0224 |
| ENSOCUT00000012405 | ENSOCUG00000012403 | GDA | DOWN | -3.84 | 0.0225 |
| ENSOCUT00000028045 | ENSOCUG00000025540 | NFYC | DOWN | -2.86 | 0.0226 |
| ENSOCUT00000008136 | ENSOCUG00000008137 | MLLT3 | DOWN | -1.93 | 0.0229 |
| ENSOCUT00000001373 | ENSOCUG00000001374 | TSHR | UP | 2.52 | 0.0230 |
| ENSOCUT00000006406 | ENSOCUG00000006404 | SLC6A7 | UP | 3.55 | 0.0231 |
| ENSOCUT00000027624 | ENSOCUG00000023553 | GFI1 | DOWN | -4.04 | 0.0231 |
| ENSOCUT00000033026 | ENSOCUG00000029006 | . | DOWN | -5.64 | 0.0232 |
| ENSOCUT00000017707 | ENSOCUG00000017702 | . | UP | 7.29 | 0.0233 |
| ENSOCUT00000001853 | ENSOCUG00000001853 | SLC15A2 | UP | 1.96 | 0.0235 |
| ENSOCUT00000027183 | ENSOCUG00000021196 | EPB41L3 | UP | 2.79 | 0.0236 |
| ENSOCUT00000016961 | ENSOCUG00000016963 | GNPDA1 | UP | 2.13 | 0.0236 |
| ENSOCUT00000013481 | ENSOCUG00000013489 | LOC100338527 | UP | 3.78 | 0.0236 |
| ENSOCUT00000013778 | ENSOCUG00000013774 | CDH8 | DOWN | -3.09 | 0.0237 |
| ENSOCUT00000010639 | ENSOCUG00000010636 | XPNPEP2 | DOWN | -4.86 | 0.0238 |
| ENSOCUT00000027139 | ENSOCUG00000024304 | FNIP1 | DOWN | -2.19 | 0.0240 |
| ENSOCUT00000033765 | ENSOCUG00000024225 | GMPPA | UP | 3.70 | 0.0240 |
| ENSOCUT00000009051 | ENSOCUG00000009055 | CD69 | DOWN | -2.91 | 0.0243 |
| ENSOCUT00000026418 | ENSOCUG00000014211 | SNTB2 | UP | 2.51 | 0.0245 |
| ENSOCUT00000015619 | ENSOCUG00000015619 | CALB2 | DOWN | -5.29 | 0.0246 |
| ENSOCUT00000016733 | ENSOCUG00000016727 | NELL2 | DOWN | -2.80 | 0.0247 |
| ENSOCUT00000015185 | ENSOCUG00000015190 | HOXA4 | UP | 7.17 | 0.0247 |
| ENSOCUT00000017739 | ENSOCUG00000017739 | BRINP2 | DOWN | -2.72 | 0.0248 |
| ENSOCUT00000017247 | ENSOCUG00000017240 | CDC5L | DOWN | -2.70 | 0.0248 |
| ENSOCUT00000027690 | ENSOCUG00000014806 | GATAD1 | DOWN | -7.07 | 0.0248 |
| ENSOCUT00000004237 | ENSOCUG00000004238 | . | DOWN | -2.55 | 0.0249 |
| ENSOCUT00000010964 | ENSOCUG00000010968 | DCTN6 | UP | 3.14 | 0.0250 |
| ENSOCUT00000023498 | ENSOCUG00000025164 | LOC100351488 | UP | 1.78 | 0.0250 |
| ENSOCUT00000010306 | ENSOCUG00000010286 | DOCK3 | DOWN | -2.46 | 0.0250 |
| ENSOCUT00000010251 | ENSOCUG00000010254 | . | UP | 2.24 | 0.0251 |
| ENSOCUT00000029666 | ENSOCUG00000022945 | ASNS | DOWN | -2.21 | 0.0251 |
| ENSOCUT00000006562 | ENSOCUG00000006561 | XAF1 | DOWN | -2.35 | 0.0253 |
| ENSOCUT00000016662 | ENSOCUG00000016662 | PPIA | DOWN | -4.68 | 0.0254 |
| ENSOCUT00000012055 | ENSOCUG00000012056 | CHODL | DOWN | -6.99 | 0.0254 |
| ENSOCUT00000034010 | ENSOCUG00000029380 | ZNF311 | DOWN | -2.90 | 0.0254 |
| ENSOCUT00000029479 | ENSOCUG00000007702 | TBC1D10C | DOWN | -2.65 | 0.0255 |
| ENSOCUT00000024070 | ENSOCUG00000025878 | FRMD7 | UP | 3.30 | 0.0256 |
| ENSOCUT00000013125 | ENSOCUG00000013126 | TRPC4AP | DOWN | -2.60 | 0.0257 |
| ENSOCUT00000025310 | ENSOCUG00000005491 | CDKN2AIP | DOWN | -4.41 | 0.0258 |
| ENSOCUT00000033286 | ENSOCUG00000002338 | SLAMF8 | UP | 2.36 | 0.0258 |
| ENSOCUT00000017963 | ENSOCUG00000017964 | TBCC | UP | 7.10 | 0.0259 |
| ENSOCUT00000006703 | ENSOCUG00000006703 | LTB | DOWN | -2.38 | 0.0259 |
| ENSOCUT00000007611 | ENSOCUG00000007607 | SAMHD1 | DOWN | -5.87 | 0.0261 |
| ENSOCUT00000010632 | ENSOCUG00000010633 | HMOX1 | UP | 3.67 | 0.0262 |
| ENSOCUT00000024825 | ENSOCUG00000022509 | . | DOWN | -4.73 | 0.0263 |
| ENSOCUT00000031256 | ENSOCUG00000024986 | TRAV23DV6 | DOWN | -4.13 | 0.0263 |
| ENSOCUT00000001840 | ENSOCUG00000001843 | MSL2 | UP | 2.38 | 0.0264 |
| ENSOCUT00000012396 | ENSOCUG00000012393 | MCCC1 | DOWN | -3.23 | 0.0264 |
| ENSOCUT00000006691 | ENSOCUG00000006694 | TNLG1E | DOWN | -4.19 | 0.0266 |
| ENSOCUT00000017125 | ENSOCUG00000017111 | KIF4A | DOWN | -4.36 | 0.0268 |
| ENSOCUT00000033207 | ENSOCUG00000002528 | LRCH4 | DOWN | -3.08 | 0.0269 |
| ENSOCUT00000004588 | ENSOCUG00000004595 | LLPH | DOWN | -4.18 | 0.0271 |
| ENSOCUT00000014747 | ENSOCUG00000014733 | ASPM | DOWN | -2.35 | 0.0271 |
| ENSOCUT00000030772 | ENSOCUG00000026206 | CCL4 | UP | 2.62 | 0.0271 |
| ENSOCUT00000006910 | ENSOCUG00000006910 | CDCP1 | DOWN | -4.07 | 0.0272 |
| ENSOCUT00000000291 | ENSOCUG00000000291 | UBXN4 | UP | 3.49 | 0.0272 |
| ENSOCUT00000007402 | ENSOCUG00000007403 | BAMBI | UP | 3.43 | 0.0274 |
| ENSOCUT00000013046 | ENSOCUG00000013046 | CXorf65 | DOWN | -4.42 | 0.0274 |
| ENSOCUT00000022392 | ENSOCUG00000025012 | ANGPT4 | UP | 2.13 | 0.0278 |
| ENSOCUT00000022268 | ENSOCUG00000021499 | . | DOWN | -6.82 | 0.0279 |
| ENSOCUT00000028340 | ENSOCUG00000028158 | ATP6V1G2 | DOWN | -2.58 | 0.0283 |
| ENSOCUT00000012357 | ENSOCUG00000012363 | AGTR2 | UP | 3.59 | 0.0289 |
| ENSOCUT00000005889 | ENSOCUG00000005891 | ELAVL2 | DOWN | -4.11 | 0.0289 |
| ENSOCUT00000002855 | ENSOCUG00000002858 | CDK1 | DOWN | -2.66 | 0.0291 |
| ENSOCUT00000009624 | ENSOCUG00000009622 | PDLIM7 | UP | 3.15 | 0.0292 |
| ENSOCUT00000010149 | ENSOCUG00000010149 | IL12A | DOWN | -3.12 | 0.0293 |
| ENSOCUT00000033803 | ENSOCUG00000004396 | NQO1 | DOWN | -3.79 | 0.0293 |
| ENSOCUT00000028716 | ENSOCUG00000026691 | SLC31A2 | DOWN | -2.40 | 0.0293 |
| ENSOCUT00000009886 | ENSOCUG00000009869 | LAMA3 | DOWN | -1.74 | 0.0293 |
| ENSOCUT00000031703 | ENSOCUG00000022519 | MXD3 | DOWN | -2.51 | 0.0296 |
| ENSOCUT00000024461 | ENSOCUG00000013950 | KIF18A | DOWN | -2.18 | 0.0296 |
| ENSOCUT00000026024 | ENSOCUG00000023845 | . | UP | 3.09 | 0.0296 |
| ENSOCUT00000013706 | ENSOCUG00000013696 | BICC1 | UP | 1.73 | 0.0296 |
| ENSOCUT00000010766 | ENSOCUG00000010772 | DCHS2 | DOWN | -2.18 | 0.0298 |
| ENSOCUT00000011946 | ENSOCUG00000011949 | LOC100354186 | DOWN | -6.77 | 0.0298 |
| ENSOCUT00000007479 | ENSOCUG00000007482 | CX3CL1 | UP | 3.03 | 0.0298 |
| ENSOCUT00000005159 | ENSOCUG00000005160 | FDFT1 | DOWN | -2.09 | 0.0302 |
| ENSOCUT00000021581 | ENSOCUG00000009918 | SCARA3 | UP | 2.65 | 0.0303 |
| ENSOCUT00000013727 | ENSOCUG00000013725 | FAM20A | UP | 3.18 | 0.0304 |
| ENSOCUT00000013296 | ENSOCUG00000013276 | COL4A2 | UP | 2.58 | 0.0311 |
| ENSOCUT00000031135 | ENSOCUG00000000120 | ADCYAP1R1 | UP | 2.76 | 0.0313 |
| ENSOCUT00000005660 | ENSOCUG00000005655 | SMC2 | DOWN | -2.08 | 0.0315 |
| ENSOCUT00000029117 | ENSOCUG00000025244 | IGFBP7 | UP | 1.95 | 0.0316 |
| ENSOCUT00000025492 | ENSOCUG00000025456 | MAP7 | DOWN | -2.50 | 0.0316 |
| ENSOCUT00000009511 | ENSOCUG00000009512 | PLA1A | UP | 2.35 | 0.0316 |
| ENSOCUT00000023645 | ENSOCUG00000022100 | AGAP3 | UP | 2.20 | 0.0318 |
| ENSOCUT00000002689 | ENSOCUG00000002688 | ATP2A1 | UP | 3.67 | 0.0320 |
| ENSOCUT00000010649 | ENSOCUG00000010649 | MKX | DOWN | -2.73 | 0.0320 |
| ENSOCUT00000033327 | ENSOCUG00000010200 | PMPCB | DOWN | -3.35 | 0.0321 |
| ENSOCUT00000006872 | ENSOCUG00000006873 | NME7 | DOWN | -4.38 | 0.0321 |
| ENSOCUT00000006639 | ENSOCUG00000006637 | SLC23A2 | DOWN | -1.85 | 0.0323 |
| ENSOCUT00000026967 | ENSOCUG00000012473 | ANKAR | DOWN | -3.13 | 0.0324 |
| ENSOCUT00000007354 | ENSOCUG00000007355 | DSG2 | DOWN | -2.25 | 0.0325 |
| ENSOCUT00000014285 | ENSOCUG00000014289 | MYC | UP | 3.12 | 0.0325 |
| ENSOCUT00000023837 | ENSOCUG00000010061 | PLEKHG4 | UP | 3.25 | 0.0328 |
| ENSOCUT00000007250 | ENSOCUG00000007251 | NOL7 | DOWN | -6.61 | 0.0329 |
| ENSOCUT00000025820 | ENSOCUG00000015169 | ATP6V0A1 | UP | 2.19 | 0.0330 |
| ENSOCUT00000015383 | ENSOCUG00000015389 | TEAD4 | UP | 4.44 | 0.0334 |
| ENSOCUT00000009583 | ENSOCUG00000009585 | SRC | DOWN | -5.33 | 0.0337 |
| ENSOCUT00000033970 | ENSOCUG00000029399 | SH2D1B | UP | 2.01 | 0.0337 |
| ENSOCUT00000016493 | ENSOCUG00000016500 | . | UP | 1.80 | 0.0338 |
| ENSOCUT00000033538 | ENSOCUG00000029499 | CWC27 | UP | 4.25 | 0.0338 |
| ENSOCUT00000005938 | ENSOCUG00000005937 | SCD5 | DOWN | -1.90 | 0.0339 |
| ENSOCUT00000016479 | ENSOCUG00000016477 | SELP | UP | 2.03 | 0.0339 |
| ENSOCUT00000031695 | ENSOCUG00000026678 | SP3 | UP | 2.70 | 0.0341 |
| ENSOCUT00000001508 | ENSOCUG00000001504 | RPA1 | DOWN | -2.69 | 0.0342 |
| ENSOCUT00000033808 | ENSOCUG00000004117 | QKI | DOWN | -4.08 | 0.0343 |
| ENSOCUT00000000558 | ENSOCUG00000000560 | HEYL | UP | 2.15 | 0.0344 |
| ENSOCUT00000024405 | ENSOCUG00000008346 | LYPD6B | DOWN | -2.78 | 0.0347 |
| ENSOCUT00000004396 | ENSOCUG00000004396 | NQO1 | DOWN | -2.92 | 0.0347 |
| ENSOCUT00000010774 | ENSOCUG00000010773 | MRVI1 | UP | 3.95 | 0.0347 |
| ENSOCUT00000030014 | ENSOCUG00000023285 | TROAP | DOWN | -2.76 | 0.0347 |
| ENSOCUT00000000272 | ENSOCUG00000000272 | EMC1 | UP | 2.18 | 0.0348 |
| ENSOCUT00000022688 | ENSOCUG00000014256 | TSR1 | DOWN | -2.85 | 0.0348 |
| ENSOCUT00000012229 | ENSOCUG00000012226 | SRP72 | UP | 2.44 | 0.0349 |
| ENSOCUT00000023201 | ENSOCUG00000025657 | . | DOWN | -2.48 | 0.0351 |
| ENSOCUT00000002418 | ENSOCUG00000002420 | MS4A3 | DOWN | -3.66 | 0.0351 |
| ENSOCUT00000029156 | ENSOCUG00000017432 | SELL | DOWN | -2.29 | 0.0353 |
| ENSOCUT00000005825 | ENSOCUG00000005822 | MROH8 | DOWN | -2.62 | 0.0353 |
| ENSOCUT00000006339 | ENSOCUG00000006339 | PATL2 | DOWN | -4.92 | 0.0354 |
| ENSOCUT00000017703 | ENSOCUG00000017700 | PLS1 | DOWN | -2.01 | 0.0354 |
| ENSOCUT00000027900 | ENSOCUG00000021196 | EPB41L3 | UP | 3.41 | 0.0354 |
| ENSOCUT00000013050 | ENSOCUG00000013043 | MYEF2 | DOWN | -1.74 | 0.0355 |
| ENSOCUT00000008983 | ENSOCUG00000008983 | CTCF | DOWN | -2.12 | 0.0356 |
| ENSOCUT00000011913 | ENSOCUG00000011916 | SNTA1 | UP | 4.25 | 0.0357 |
| ENSOCUT00000014065 | ENSOCUG00000014067 | NPC2 | DOWN | -3.25 | 0.0359 |
| ENSOCUT00000013111 | ENSOCUG00000013111 | PCOLCE2 | DOWN | -2.10 | 0.0360 |
| ENSOCUT00000020944 | ENSOCUG00000014258 | TRAC | DOWN | -4.44 | 0.0360 |
| ENSOCUT00000005431 | ENSOCUG00000005432 | TM4SF19 | UP | 3.30 | 0.0360 |
| ENSOCUT00000028678 | ENSOCUG00000026609 | GLMP | UP | 2.87 | 0.0360 |
| ENSOCUT00000000789 | ENSOCUG00000000787 | PTPRO | UP | 1.94 | 0.0364 |
| ENSOCUT00000024881 | ENSOCUG00000008378 | DPP10 | UP | 2.98 | 0.0365 |
| ENSOCUT00000000779 | ENSOCUG00000000779 | RSPO2 | UP | 2.50 | 0.0365 |
| ENSOCUT00000004355 | ENSOCUG00000004354 | PABPC1 | UP | 2.12 | 0.0368 |
| ENSOCUT00000011807 | ENSOCUG00000011804 | FAM91A1 | UP | 2.69 | 0.0368 |
| ENSOCUT00000006632 | ENSOCUG00000006632 | MREG | UP | 2.01 | 0.0369 |
| ENSOCUT00000011635 | ENSOCUG00000011634 | SMPD3 | DOWN | -2.23 | 0.0369 |
| ENSOCUT00000004081 | ENSOCUG00000004078 | OSBPL6 | DOWN | -3.05 | 0.0370 |
| ENSOCUT00000031537 | ENSOCUG00000009184 | CTHRC1 | UP | 2.47 | 0.0370 |
| ENSOCUT00000029874 | ENSOCUG00000021497 | ARHGAP19 | DOWN | -1.78 | 0.0370 |
| ENSOCUT00000015415 | ENSOCUG00000015410 | NDC80 | DOWN | -2.40 | 0.0371 |
| ENSOCUT00000008545 | ENSOCUG00000008545 | RRM2B | DOWN | -4.22 | 0.0372 |
| ENSOCUT00000007546 | ENSOCUG00000007539 | FANCM | DOWN | -2.23 | 0.0373 |
| ENSOCUT00000005657 | ENSOCUG00000005661 | XKRX | DOWN | -4.76 | 0.0373 |
| ENSOCUT00000012934 | ENSOCUG00000012939 | ENPP5 | DOWN | -2.65 | 0.0376 |
| ENSOCUT00000015258 | ENSOCUG00000015247 | ZDHHC14 | UP | 2.20 | 0.0377 |
| ENSOCUT00000015308 | ENSOCUG00000015316 | EBP | DOWN | -1.76 | 0.0379 |
| ENSOCUT00000025134 | ENSOCUG00000027178 | . | UP | 2.50 | 0.0379 |
| ENSOCUT00000024277 | ENSOCUG00000021980 | DNAJC14 | UP | 1.62 | 0.0379 |
| ENSOCUT00000016409 | ENSOCUG00000016412 | ORC4 | DOWN | -2.40 | 0.0380 |
| ENSOCUT00000017309 | ENSOCUG00000017308 | FLOT1 | UP | 2.00 | 0.0382 |
| ENSOCUT00000003416 | ENSOCUG00000003419 | RELL1 | UP | 1.59 | 0.0384 |
| ENSOCUT00000029645 | ENSOCUG00000021424 | VPS72 | DOWN | -5.98 | 0.0386 |
| ENSOCUT00000016217 | ENSOCUG00000016222 | TXLNB | UP | 2.37 | 0.0391 |
| ENSOCUT00000002632 | ENSOCUG00000002624 | NRK | UP | 1.87 | 0.0391 |
| ENSOCUT00000006490 | ENSOCUG00000006493 | STMN1 | DOWN | -2.52 | 0.0393 |
| ENSOCUT00000012337 | ENSOCUG00000012338 | DDX3X | DOWN | -1.72 | 0.0393 |
| ENSOCUT00000000541 | ENSOCUG00000000540 | KCNIP1 | UP | 2.48 | 0.0393 |
| ENSOCUT00000006713 | ENSOCUG00000006714 | MMP2 | UP | 1.77 | 0.0394 |
| ENSOCUT00000017022 | ENSOCUG00000017023 | ATF1 | DOWN | -2.41 | 0.0396 |
| ENSOCUT00000015129 | ENSOCUG00000015126 | FRMD6 | DOWN | -6.07 | 0.0398 |
| ENSOCUT00000010116 | ENSOCUG00000010104 | FN1 | UP | 1.81 | 0.0398 |
| ENSOCUT00000002263 | ENSOCUG00000002260 | ACSS2 | DOWN | -1.76 | 0.0399 |
| ENSOCUT00000002758 | ENSOCUG00000002754 | ABCC3 | UP | 3.88 | 0.0401 |
| ENSOCUT00000015999 | ENSOCUG00000015995 | SLC17A8 | UP | 2.98 | 0.0401 |
| ENSOCUT00000012331 | ENSOCUG00000012328 | RNF128 | UP | 2.06 | 0.0403 |
| ENSOCUT00000015421 | ENSOCUG00000015423 | ZDHHC23 | DOWN | -2.33 | 0.0404 |
| ENSOCUT00000015347 | ENSOCUG00000015345 | SP4 | DOWN | -1.78 | 0.0405 |
| ENSOCUT00000027440 | ENSOCUG00000025119 | RELT | DOWN | -2.85 | 0.0407 |
| ENSOCUT00000021867 | ENSOCUG00000007461 | NAV1 | UP | 1.98 | 0.0411 |
| ENSOCUT00000009264 | ENSOCUG00000009260 | PLCH1 | DOWN | -2.28 | 0.0411 |
| ENSOCUT00000014082 | ENSOCUG00000014085 | CREG2 | DOWN | -3.99 | 0.0412 |
| ENSOCUT00000029729 | ENSOCUG00000002846 | PSME1 | UP | 2.43 | 0.0413 |
| ENSOCUT00000005905 | ENSOCUG00000005903 | SSBP3 | DOWN | -2.65 | 0.0413 |
| ENSOCUT00000024792 | ENSOCUG00000027090 | . | UP | 4.45 | 0.0414 |
| ENSOCUT00000011535 | ENSOCUG00000011530 | PHF2 | UP | 4.48 | 0.0417 |
| ENSOCUT00000028289 | ENSOCUG00000000268 | PCDH15 | UP | 1.94 | 0.0420 |
| ENSOCUT00000005851 | ENSOCUG00000005852 | LECT1 | DOWN | -2.07 | 0.0424 |
| ENSOCUT00000033785 | ENSOCUG00000013725 | FAM20A | UP | 1.94 | 0.0425 |
| ENSOCUT00000005316 | ENSOCUG00000005316 | MAPKAPK3 | UP | 1.60 | 0.0426 |
| ENSOCUT00000008051 | ENSOCUG00000008051 | WASF1 | DOWN | -2.78 | 0.0427 |
| ENSOCUT00000032152 | ENSOCUG00000025220 | . | UP | 4.27 | 0.0428 |
| ENSOCUT00000013449 | ENSOCUG00000013449 | RBPJ | UP | 3.38 | 0.0429 |
| ENSOCUT00000007146 | ENSOCUG00000007145 | KIAA0586 | DOWN | -2.49 | 0.0430 |
| ENSOCUT00000004137 | ENSOCUG00000004136 | SEPT-7 | DOWN | -1.96 | 0.0431 |
| ENSOCUT00000001920 | ENSOCUG00000001923 | CXCR4 | DOWN | -2.36 | 0.0431 |
| ENSOCUT00000024610 | ENSOCUG00000021506 | FOS | DOWN | -2.09 | 0.0432 |
| ENSOCUT00000012429 | ENSOCUG00000012432 | HS3ST3B1 | DOWN | -2.41 | 0.0433 |
| ENSOCUT00000002146 | ENSOCUG00000002147 | KHNYN | UP | 2.85 | 0.0433 |
| ENSOCUT00000005636 | ENSOCUG00000005634 | RAB25 | DOWN | -4.15 | 0.0433 |
| ENSOCUT00000030995 | ENSOCUG00000003186 | HPRT1 | UP | 1.63 | 0.0434 |
| ENSOCUT00000003770 | ENSOCUG00000003763 | PTPN22 | DOWN | -2.38 | 0.0435 |
| ENSOCUT00000014654 | ENSOCUG00000014647 | USP15 | DOWN | -1.51 | 0.0436 |
| ENSOCUT00000009294 | ENSOCUG00000009297 | RAB6A | DOWN | -2.86 | 0.0437 |
| ENSOCUT00000013797 | ENSOCUG00000013802 | ALDOC | DOWN | -2.02 | 0.0439 |
| ENSOCUT00000013056 | ENSOCUG00000013049 | PLXND1 | UP | 3.68 | 0.0439 |
| ENSOCUT00000016263 | ENSOCUG00000016268 | RCN1 | UP | 1.70 | 0.0439 |
| ENSOCUT00000028251 | ENSOCUG00000022602 | . | UP | 2.73 | 0.0440 |
| ENSOCUT00000006895 | ENSOCUG00000006893 | MARCO | UP | 2.94 | 0.0441 |
| ENSOCUT00000008723 | ENSOCUG00000008724 | ERCC6L | DOWN | -2.45 | 0.0443 |
| ENSOCUT00000003162 | ENSOCUG00000003157 | DCBLD1 | DOWN | -3.56 | 0.0446 |
| ENSOCUT00000003314 | ENSOCUG00000003313 | LOX | UP | 1.64 | 0.0446 |
| ENSOCUT00000012185 | ENSOCUG00000012185 | KIAA1211 | DOWN | -2.71 | 0.0446 |
| ENSOCUT00000032094 | ENSOCUG00000022392 | PIGZ | UP | 3.07 | 0.0447 |
| ENSOCUT00000031511 | ENSOCUG00000023599 | APOD | UP | 2.94 | 0.0449 |
| ENSOCUT00000015588 | ENSOCUG00000015599 | GEMIN6 | DOWN | -5.69 | 0.0449 |
| ENSOCUT00000014538 | ENSOCUG00000014539 | KDELC2 | UP | 3.21 | 0.0450 |
| ENSOCUT00000003715 | ENSOCUG00000003715 | LAX1 | DOWN | -2.57 | 0.0450 |
| ENSOCUT00000005200 | ENSOCUG00000005202 | S100A11 | UP | 1.72 | 0.0451 |
| ENSOCUT00000033332 | ENSOCUG00000025851 | CPM | UP | 2.27 | 0.0451 |
| ENSOCUT00000030336 | ENSOCUG00000022237 | PRELID3A | UP | 2.76 | 0.0452 |
| ENSOCUT00000026395 | ENSOCUG00000024104 | . | UP | 5.15 | 0.0454 |
| ENSOCUT00000009259 | ENSOCUG00000009261 | TMEM40 | UP | 2.50 | 0.0455 |
| ENSOCUT00000014911 | ENSOCUG00000014919 | SNX12 | DOWN | -3.19 | 0.0456 |
| ENSOCUT00000004297 | ENSOCUG00000004293 | EPS15 | DOWN | -2.92 | 0.0457 |
| ENSOCUT00000013757 | ENSOCUG00000013761 | GAREM1 | UP | 3.19 | 0.0459 |
| ENSOCUT00000001689 | ENSOCUG00000001689 | RTKN2 | DOWN | -2.58 | 0.0459 |
| ENSOCUT00000007607 | ENSOCUG00000007606 | MAP2K6 | DOWN | -2.08 | 0.0459 |
| ENSOCUT00000005204 | ENSOCUG00000005203 | PHTF2 | DOWN | -2.06 | 0.0459 |
| ENSOCUT00000011294 | ENSOCUG00000011289 | ALCAM | UP | 1.51 | 0.0459 |
| ENSOCUT00000021988 | ENSOCUG00000027603 | PDGFB | UP | 2.99 | 0.0461 |
| ENSOCUT00000013329 | ENSOCUG00000013333 | . | DOWN | -2.51 | 0.0463 |
| ENSOCUT00000012252 | ENSOCUG00000012254 | SNAI2 | UP | 1.72 | 0.0464 |
| ENSOCUT00000015462 | ENSOCUG00000015431 | DOCK10 | UP | 1.75 | 0.0464 |
| ENSOCUT00000029797 | ENSOCUG00000024897 | . | DOWN | -6.03 | 0.0464 |
| ENSOCUT00000010437 | ENSOCUG00000010440 | MSI2 | DOWN | -1.60 | 0.0465 |
| ENSOCUT00000004846 | ENSOCUG00000004847 | GAP43 | DOWN | -2.28 | 0.0465 |
| ENSOCUT00000014920 | ENSOCUG00000014918 | SEC31A | UP | 2.18 | 0.0467 |
| ENSOCUT00000014931 | ENSOCUG00000014931 | . | DOWN | -1.99 | 0.0470 |
| ENSOCUT00000012491 | ENSOCUG00000012491 | OSGEPL1 | DOWN | -2.57 | 0.0470 |
| ENSOCUT00000004623 | ENSOCUG00000004627 | APH1B | UP | 1.86 | 0.0472 |
| ENSOCUT00000016936 | ENSOCUG00000016943 | FAM25A | DOWN | -3.03 | 0.0472 |
| ENSOCUT00000010194 | ENSOCUG00000010192 | CDH5 | UP | 1.70 | 0.0475 |
| ENSOCUT00000014751 | ENSOCUG00000014710 | USH2A | DOWN | -2.29 | 0.0476 |
| ENSOCUT00000033488 | ENSOCUG00000006595 | UBA7 | DOWN | -2.09 | 0.0476 |
| ENSOCUT00000026050 | ENSOCUG00000024804 | BTBD19 | UP | 2.09 | 0.0479 |
| ENSOCUT00000005291 | ENSOCUG00000005294 | RRAGD | UP | 1.68 | 0.0479 |
| ENSOCUT00000007583 | ENSOCUG00000007585 | ARGLU1 | DOWN | -3.16 | 0.0481 |
| ENSOCUT00000005092 | ENSOCUG00000005094 | INHBB | UP | 2.37 | 0.0483 |
| ENSOCUT00000024138 | ENSOCUG00000027825 | AKIP1 | DOWN | -2.90 | 0.0484 |
| ENSOCUT00000007505 | ENSOCUG00000007500 | HMGXB3 | DOWN | -3.22 | 0.0484 |
| ENSOCUT00000006358 | ENSOCUG00000006357 | ADAMTS12 | UP | 1.66 | 0.0485 |
| ENSOCUT00000031514 | ENSOCUG00000021320 | RRBP1 | UP | 2.27 | 0.0486 |
| ENSOCUT00000024937 | ENSOCUG00000003165 | SCUBE2 | UP | 2.38 | 0.0490 |
| ENSOCUT00000001097 | ENSOCUG00000001097 | CGRRF1 | UP | 2.70 | 0.0492 |
| ENSOCUT00000004093 | ENSOCUG00000004093 | PUS7L | DOWN | -1.86 | 0.0492 |
| ENSOCUT00000007731 | ENSOCUG00000007730 | LAD1 | DOWN | -3.08 | 0.0492 |
| ENSOCUT00000014584 | ENSOCUG00000014585 | ZDHHC2 | DOWN | -2.62 | 0.0492 |
| ENSOCUT00000029029 | ENSOCUG00000026567 | RLA-DMB | UP | 1.59 | 0.0495 |
| ENSOCUT00000031942 | ENSOCUG00000009761 | TMEM71 | DOWN | -2.20 | 0.0497 |
| ENSOCUT00000005796 | ENSOCUG00000012774 | KRT4 | DOWN | -5.47 | 0.0498 |
| ENSOCUT00000027484 | ENSOCUG00000026887 | . | DOWN | -4.72 | 0.0498 |
