# Supplementary material for: Comprehensive analysis of circRNA expression pattern and circRNA-miRNA-mRNA network in the pathogenesis of atherosclerosis in rabbits
Source: Aging (Albany NY). 2018 Sep 6;10(9):2266–83. doi: 10.18632/aging.101541 (PMC6188486; doi:10.18632/aging.101541)
Supplement: Supplementary Table S2 [file aging-10-101541-s002.docx]

**Supplementary Table S2.** **Differential expressed circRNA identified by edgeR package.**

| **CircRNA_id** | **Chr** | **Strand** | **Start** | **End** | **Best_hit_transcript** | **Gene_id** | **Gene_name** | **Status** | **LogFC** | **Pvalue** |
| --- | --- | --- | --- | --- | --- | --- | --- | --- | --- | --- |
| ocu-cirR-novel-15310 | 19 | + | 29686768 | 29702746 | ENSOCUT00000009780 | ENSOCUG00000009775 | TEX14 | DOWN | -7.68 | 1.95E-06 |
| ocu-cirR-novel-17881 | 7 | + | 129334306 | 129335069 | ENSOCUT00000015004 | ENSOCUG00000014988 | COL3A1 | UP | 11.89 | 6.00E-06 |
| ocu-cirR-novel-13067 | 14 | - | 4434269 | 4440070 | ENSOCUT00000007175 | ENSOCUG00000007174 | SATB1 | DOWN | -5.38 | 0.0001 |
| ocu-cirR-novel-16238 | 2 | + | 92200007 | 92204484 | ENSOCUT00000009541 | ENSOCUG00000009532 | AFF3 | DOWN | -6.25 | 0.0002 |
| ocu-cirR-novel-10713 | 1 | + | 8168152 | 8174354 | ENSOCUT00000009288 | ENSOCUG00000009279 | ABCA1 | UP | 2.37 | 0.0003 |
| ocu-cirR-novel-12689 | 13 | - | 99301881 | 99316626 | ENSOCUT00000015441 | ENSOCUG00000015438 | MIER1 | DOWN | -6.05 | 0.0004 |
| ocu-cirR-novel-11221 | 11 | + | 78375884 | 78377281 | ENSOCUT00000021575 | ENSOCUG00000010466 | KIF2A | DOWN | -5.80 | 0.0005 |
| ocu-cirR-novel-16820 | 3 | + | 17412145 | 17413369 | ENSOCUT00000008262 | ENSOCUG00000008254 | SEC24A | DOWN | -5.78 | 0.0006 |
| ocu-cirR-novel-10714 | 1 | + | 8168152 | 8196220 | ENSOCUT00000009288 | ENSOCUG00000009279 | ABCA1 | UP | 2.62 | 0.0008 |
| ocu-cirR-novel-13631 | 15 | + | 82165926 | 82171117 | ENSOCUT00000008150 | ENSOCUG00000008151 | CENPC | DOWN | -5.64 | 0.0009 |
| ocu-cirR-novel-11489 | 12 | - | 143102337 | 143104750 | ENSOCUT00000000061 | ENSOCUG00000000061 | FBXO5 | DOWN | -5.76 | 0.0011 |
| ocu-cirR-novel-17879 | 7 | + | 129322965 | 129324011 | ENSOCUT00000015004 | ENSOCUG00000014988 | COL3A1 | UP | 10.51 | 0.0013 |
| ocu-cirR-novel-11502 | 12 | + | 145053078 | 145065992 | ENSOCUT00000023944 | ENSOCUG00000026063 | SCAF8 | UP | 5.61 | 0.0017 |
| ocu-cirR-novel-18489 | 8 | - | 30605109 | 30613253 | ENSOCUT00000001957 | ENSOCUG00000001956 | C12orf4 | DOWN | -6.96 | 0.0026 |
| ocu-cirR-novel-19104 | 9 | + | 73418013 | 73419329 | ENSOCUT00000007198 | ENSOCUG00000007201 | RNF138 | DOWN | -5.35 | 0.0027 |
| ocu-cirR-novel-12026 | 13 | + | 120959864 | 120961322 | ENSOCUT00000017939 | ENSOCUG00000017939 | GPBP1L1 | DOWN | -3.01 | 0.0028 |
| ocu-cirR-novel-19049 | 9 | - | 62360114 | 62362975 | ENSOCUT00000004853 | ENSOCUG00000004848 | ROCK1 | DOWN | -3.80 | 0.0030 |
| ocu-cirR-novel-13564 | 15 | - | 6852154 | 6908559 | ENSOCUT00000004483 | ENSOCUG00000004484 | GLRB | DOWN | -6.57 | 0.0034 |
| ocu-cirR-novel-14852 | 18 | + | 40416065 | 40422353 | ENSOCUT00000016710 | ENSOCUG00000016706 | KIF11 | DOWN | -5.66 | 0.0035 |
| ocu-cirR-novel-16747 | 3 | + | 140715159 | 140720827 | ENSOCUT00000015771 | ENSOCUG00000015771 | SQLE | DOWN | -5.37 | 0.0035 |
| ocu-cirR-novel-15415 | 19 | - | 46615637 | 46617411 | ENSOCUT00000020891 | ENSOCUG00000010815 | CDC27 | DOWN | -5.58 | 0.0035 |
| ocu-cirR-novel-16997 | 3 | - | 57989652 | 57990062 | ENSOCUT00000009624 | ENSOCUG00000009622 | PDLIM7 | DOWN | -6.11 | 0.0041 |
| ocu-cirR-novel-18456 | 8 | + | 23927649 | 23938833 | ENSOCUT00000000793 | ENSOCUG00000000792 | EPS8 | UP | 5.36 | 0.0043 |
| ocu-cirR-novel-14104 | 16 | + | 74511703 | 74545651 | ENSOCUT00000013976 | ENSOCUG00000013965 | KCNT2 | DOWN | -3.27 | 0.0044 |
| ocu-cirR-novel-12686 | 13 | - | 99298875 | 99306680 | ENSOCUT00000015441 | ENSOCUG00000015438 | MIER1 | DOWN | -3.99 | 0.0046 |
| ocu-cirR-novel-15034 | 18 | + | 67111606 | 67112237 | ENSOCUT00000003143 | ENSOCUG00000003141 | SEC23IP | UP | 5.97 | 0.0051 |
| ocu-cirR-novel-18453 | 8 | + | 23913922 | 23922779 | ENSOCUT00000000793 | ENSOCUG00000000792 | EPS8 | UP | 3.04 | 0.0051 |
| ocu-cirR-novel-17059 | 3 | - | 74430690 | 74451762 | ENSOCUT00000005262 | ENSOCUG00000005263 | TOX | DOWN | -5.68 | 0.0052 |
| ocu-cirR-novel-18036 | 7 | - | 156718397 | 156747291 | ENSOCUT00000010116 | ENSOCUG00000010104 | FN1 | UP | 6.08 | 0.0054 |
| ocu-cirR-novel-17934 | 7 | - | 13804206 | 13809649 | ENSOCUT00000027794 | ENSOCUG00000013448 | CNOT4 | DOWN | -5.53 | 0.0057 |
| ocu-cirR-novel-13178 | 14 | + | 78491655 | 78504435 | ENSOCUT00000021686 | ENSOCUG00000012358 | ATP11B | DOWN | -5.34 | 0.0058 |
| ocu-cirR-novel-12940 | 14 | + | 22575809 | 22578990 | ENSOCUT00000017085 | ENSOCUG00000009828 | ARPP21 | DOWN | -6.07 | 0.0061 |
| ocu-cirR-novel-18685 | 8 | + | 79491108 | 79525328 | ENSOCUT00000010323 | ENSOCUG00000010325 | SLAIN1 | DOWN | -5.87 | 0.0062 |
| ocu-cirR-novel-17230 | 4 | + | 33680963 | 33681213 | ENSOCUT00000016807 | ENSOCUG00000016805 | SPATS2 | UP | 1.68 | 0.0066 |
| ocu-cirR-novel-12623 | 13 | + | 80269742 | 80270371 | ENSOCUT00000015687 | ENSOCUG00000015685 | SSX2IP | DOWN | -2.25 | 0.0073 |
| ocu-cirR-novel-16367 | 20 | + | 24397735 | 24399967 | ENSOCUT00000004028 | ENSOCUG00000004026 | CEP128 | DOWN | -3.03 | 0.0076 |
| ocu-cirR-novel-13231 | 14 | - | 91011100 | 91030464 | ENSOCUT00000001799 | ENSOCUG00000001798 | ACAP2 | UP | 5.71 | 0.0078 |
| ocu-cirR-novel-19349 | X | + | 67489348 | 67503890 | ENSOCUT00000013690 | ENSOCUG00000013687 | IL13RA1 | UP | 4.10 | 0.0081 |
| ocu-cirR-novel-11404 | 12 | - | 125377748 | 125381631 | ENSOCUT00000011625 | ENSOCUG00000011619 | HBS1L | DOWN | -5.37 | 0.0086 |
| ocu-cirR-novel-15993 | 2 | + | 31086996 | 31097774 | ENSOCUT00000017142 | ENSOCUG00000017135 | SLC30A9 | UP | 5.08 | 0.0095 |
| ocu-cirR-novel-11768 | 12 | - | 72613131 | 72613553 | ENSOCUT00000008943 | ENSOCUG00000008929 | SNX14 | DOWN | -5.18 | 0.0097 |
| ocu-cirR-novel-18686 | 8 | + | 79491108 | 79533439 | ENSOCUT00000010323 | ENSOCUG00000010325 | SLAIN1 | DOWN | -4.42 | 0.0097 |
| ocu-cirR-novel-11832 | 12 | - | 87077105 | 87078452 | ENSOCUT00000000987 | ENSOCUG00000000987 | PNISR | DOWN | -5.04 | 0.0104 |
| ocu-cirR-novel-10734 | 1 | - | 92257250 | 92273390 | ENSOCUT00000014407 | ENSOCUG00000014409 | CCDC15 | DOWN | -5.45 | 0.0109 |
| ocu-cirR-novel-13065 | 14 | - | 4413944 | 4440070 | ENSOCUT00000007175 | ENSOCUG00000007174 | SATB1 | DOWN | -5.94 | 0.0116 |
| ocu-cirR-novel-10828 | 10 | - | 30623565 | 30635526 | ENSOCUT00000016803 | ENSOCUG00000016806 | GLCCI1 | DOWN | -4.16 | 0.0120 |
| ocu-cirR-novel-14731 | 18 | + | 13881035 | 13883747 | ENSOCUT00000030381 | ENSOCUG00000012547 | USP54 | DOWN | -5.07 | 0.0124 |
| ocu-cirR-novel-18298 | 7 | - | 82628831 | 82747782 | ENSOCUT00000002103 | ENSOCUG00000002102 | GTDC1 | UP | 3.30 | 0.0125 |
| ocu-cirR-novel-13059 | 14 | - | 4394721 | 4404438 | ENSOCUT00000007175 | ENSOCUG00000007174 | SATB1 | DOWN | -5.88 | 0.0125 |
| ocu-cirR-novel-10619 | 1 | - | 59434728 | 59439064 | ENSOCUT00000026662 | ENSOCUG00000009444 | ALDH1A1 | DOWN | -6.85 | 0.0126 |
| ocu-cirR-novel-14063 | 16 | - | 69620189 | 69620791 | ENSOCUT00000029621 | ENSOCUG00000022087 | RNPEP | UP | 2.54 | 0.0126 |
| ocu-cirR-novel-18192 | 7 | + | 41341201 | 41343706 | ENSOCUT00000008792 | ENSOCUG00000008790 | KMT2E | DOWN | -6.83 | 0.0126 |
| ocu-cirR-novel-14100 | 16 | + | 74119137 | 74125235 | ENSOCUT00000014747 | ENSOCUG00000014733 | ASPM | DOWN | -5.22 | 0.0131 |
| ocu-cirR-novel-15632 | 2 | - | 123797168 | 123797575 | ENSOCUT00000013572 | ENSOCUG00000013563 | EHBP1 | UP | 2.77 | 0.0135 |
| ocu-cirR-novel-11651 | 12 | + | 50402510 | 50402779 | ENSOCUT00000015526 | ENSOCUG00000015520 | PHF3 | DOWN | -5.01 | 0.0136 |
| ocu-cirR-novel-11291 | 12 | - | 101899158 | 101900307 | ENSOCUT00000000598 | ENSOCUG00000000598 | HDAC2 | DOWN | -5.41 | 0.0140 |
| ocu-cirR-novel-11587 | 12 | + | 32388501 | 32394206 | ENSOCUT00000013444 | ENSOCUG00000013438 | BICRAL | DOWN | -5.44 | 0.0141 |
| ocu-cirR-novel-12789 | 14 | - | 121941836 | 121963232 | ENSOCUT00000008311 | ENSOCUG00000008311 | ST3GAL6 | DOWN | -3.16 | 0.0142 |
| ocu-cirR-novel-12703 | 13 | + | 99410241 | 99413287 | ENSOCUT00000031843 | ENSOCUG00000021747 | WDR78 | DOWN | -5.54 | 0.0143 |
| ocu-cirR-novel-14173 | 17 | + | 15851419 | 15865422 | ENSOCUT00000015100 | ENSOCUG00000015094 | ZNF280D | DOWN | -5.39 | 0.0144 |
| ocu-cirR-novel-13035 | 14 | + | 36146240 | 36186286 | ENSOCUT00000017709 | ENSOCUG00000017709 | TRPC1 | DOWN | -2.86 | 0.0152 |
| ocu-cirR-novel-15560 | 2 | - | 111668624 | 111679391 | ENSOCUT00000000198 | ENSOCUG00000000197 | HK2 | UP | 5.43 | 0.0153 |
| ocu-cirR-novel-14397 | 17 | - | 32571982 | 32573090 | ENSOCUT00000003796 | ENSOCUG00000003792 | THBS1 | UP | 6.36 | 0.0153 |
| ocu-cirR-novel-17880 | 7 | + | 129326393 | 129333790 | ENSOCUT00000015004 | ENSOCUG00000014988 | COL3A1 | UP | 6.41 | 0.0154 |
| ocu-cirR-novel-19136 | 9 | + | 86183012 | 86183669 | ENSOCUT00000009705 | ENSOCUG00000009706 | SETBP1 | UP | 2.38 | 0.0155 |
| ocu-cirR-novel-17032 | 3 | - | 67633566 | 67634720 | ENSOCUT00000016403 | ENSOCUG00000016393 | RB1CC1 | DOWN | -4.93 | 0.0156 |
| ocu-cirR-novel-18311 | 7 | - | 86632339 | 86637154 | ENSOCUT00000016409 | ENSOCUG00000016412 | ORC4 | DOWN | -4.95 | 0.0156 |
| ocu-cirR-novel-16092 | 2 | + | 51110466 | 51110927 | ENSOCUT00000011078 | ENSOCUG00000011080 | GALNT7 | DOWN | -2.89 | 0.0169 |
| ocu-cirR-novel-15941 | 2 | + | 173809488 | 173816939 | ENSOCUT00000033507 | ENSOCUG00000015422 | NCOA1 | DOWN | -3.22 | 0.0170 |
| ocu-cirR-novel-13356 | 15 | + | 13958701 | 13998582 | ENSOCUT00000021781 | ENSOCUG00000022271 | . | DOWN | -4.88 | 0.0172 |
| ocu-cirR-novel-16865 | 3 | + | 20703568 | 20703896 | ENSOCUT00000008425 | ENSOCUG00000008421 | KDM3B | DOWN | -3.21 | 0.0179 |
| ocu-cirR-novel-17762 | 7 | - | 103864111 | 103864620 | ENSOCUT00000002908 | ENSOCUG00000002909 | GRB14 | DOWN | -2.37 | 0.0185 |
| ocu-cirR-novel-19088 | 9 | - | 72948549 | 72980237 | ENSOCUT00000013283 | ENSOCUG00000013287 | B4GALT6 | DOWN | -3.14 | 0.0186 |
| ocu-cirR-novel-17477 | 4 | + | 80225421 | 80271729 | ENSOCUT00000000883 | ENSOCUG00000000881 | ANO4 | UP | 3.16 | 0.0187 |
| ocu-cirR-novel-15308 | 19 | + | 29686768 | 29691580 | ENSOCUT00000009780 | ENSOCUG00000009775 | TEX14 | DOWN | -4.88 | 0.0195 |
| ocu-cirR-novel-10997 | 11 | - | 206330 | 228312 | ENSOCUT00000009680 | ENSOCUG00000009680 | HOMER1 | UP | 2.91 | 0.0195 |
| ocu-cirR-novel-10985 | 11 | - | 18344243 | 18348693 | ENSOCUT00000004441 | ENSOCUG00000004434 | CHD1 | DOWN | -5.27 | 0.0197 |
| ocu-cirR-novel-15395 | 19 | + | 43260969 | 43263032 | ENSOCUT00000015182 | ENSOCUG00000015169 | ATP6V0A1 | UP | 4.81 | 0.0203 |
| ocu-cirR-novel-10998 | 11 | - | 217983 | 228312 | ENSOCUT00000009680 | ENSOCUG00000009680 | HOMER1 | UP | 2.20 | 0.0206 |
| ocu-cirR-novel-15840 | 2 | + | 154339648 | 154356431 | ENSOCUT00000005878 | ENSOCUG00000005878 | MEMO1 | UP | 2.94 | 0.0215 |
| ocu-cirR-novel-11533 | 12 | - | 20849575 | 20858811 | ENSOCUT00000007067 | ENSOCUG00000007056 | TNXB | DOWN | -6.22 | 0.0219 |
| ocu-cirR-novel-11169 | 11 | + | 66896480 | 66928069 | ENSOCUT00000014457 | ENSOCUG00000014454 | PARP8 | UP | 5.21 | 0.0222 |
| ocu-cirR-novel-10858 | 10 | - | 34510939 | 34514290 | ENSOCUT00000012284 | ENSOCUG00000012264 | COL1A2 | DOWN | -6.17 | 0.0225 |
| ocu-cirR-novel-16123 | 2 | + | 61930697 | 62011256 | ENSOCUT00000003898 | ENSOCUG00000003897 | PRIMPOL | DOWN | -3.32 | 0.0234 |
| ocu-cirR-novel-14097 | 16 | + | 73638737 | 73653067 | ENSOCUT00000025353 | ENSOCUG00000015970 | DENND1B | DOWN | -5.05 | 0.0235 |
| ocu-cirR-novel-17249 | 4 | + | 38264013 | 38264668 | ENSOCUT00000013524 | ENSOCUG00000013517 | NCKAP1L | DOWN | -2.76 | 0.0243 |
| ocu-cirR-novel-15353 | 19 | - | 3828638 | 3832563 | ENSOCUT00000010472 | ENSOCUG00000010460 | NCOR1 | DOWN | -4.68 | 0.0245 |
| ocu-cirR-novel-14939 | 18 | - | 50043572 | 50051770 | ENSOCUT00000002777 | ENSOCUG00000002776 | C10orf76 | UP | 2.87 | 0.0257 |
| ocu-cirR-novel-17160 | 4 | + | 11783831 | 11784210 | ENSOCUT00000000708 | ENSOCUG00000000708 | CDS2 | DOWN | -2.57 | 0.0266 |
| ocu-cirR-novel-17142 | 3 | - | 89467281 | 89488656 | ENSOCUT00000013844 | ENSOCUG00000013843 | STAU2 | DOWN | -2.61 | 0.0269 |
| ocu-cirR-novel-17381 | 4 | - | 59074474 | 59086861 | ENSOCUT00000016706 | ENSOCUG00000016701 | PPP1R12A | DOWN | -4.64 | 0.0275 |
| ocu-cirR-novel-18684 | 8 | - | 78931253 | 78934542 | ENSOCUT00000011928 | ENSOCUG00000011902 | MYCBP2 | DOWN | -4.74 | 0.0280 |
| ocu-cirR-novel-15409 | 19 | + | 45618183 | 45623846 | ENSOCUT00000017523 | ENSOCUG00000017526 | CCDC43 | DOWN | -4.74 | 0.0281 |
| ocu-cirR-novel-16029 | 2 | + | 37703717 | 37715255 | ENSOCUT00000012352 | ENSOCUG00000012352 | DCUN1D4 | DOWN | -4.76 | 0.0281 |
| ocu-cirR-novel-18487 | 8 | - | 28955935 | 28956448 | ENSOCUT00000006086 | ENSOCUG00000006082 | DDX11 | DOWN | -4.65 | 0.0281 |
| ocu-cirR-novel-11087 | 11 | + | 56251332 | 56252735 | ENSOCUT00000005260 | ENSOCUG00000005257 | DNAJC21 | DOWN | -4.75 | 0.0285 |
| ocu-cirR-novel-18638 | 8 | + | 53975823 | 53981784 | ENSOCUT00000010582 | ENSOCUG00000010580 | ELF1 | DOWN | -5.28 | 0.0286 |
| ocu-cirR-novel-18590 | 8 | + | 44820975 | 44824478 | ENSOCUT00000006154 | ENSOCUG00000006153 | PSPC1 | DOWN | -1.74 | 0.0289 |
| ocu-cirR-novel-17713 | 6 | + | 4199105 | 4201688 | ENSOCUT00000008407 | ENSOCUG00000008408 | ATF7IP2 | DOWN | -5.94 | 0.0290 |
| ocu-cirR-novel-15230 | 19 | - | 25484162 | 25485563 | ENSOCUT00000014508 | ENSOCUG00000014504 | TADA2A | UP | 4.71 | 0.0292 |
| ocu-cirR-novel-17858 | 7 | + | 121475965 | 121486340 | ENSOCUT00000028028 | ENSOCUG00000003008 | ITGA4 | DOWN | -2.96 | 0.0299 |
| ocu-cirR-novel-18642 | 8 | + | 54027700 | 54028992 | ENSOCUT00000010582 | ENSOCUG00000010580 | ELF1 | DOWN | -1.62 | 0.0299 |
| ocu-cirR-novel-10065 | 1 | + | 113692486 | 113696970 | ENSOCUT00000008304 | ENSOCUG00000008303 | MMP12 | UP | 5.24 | 0.0308 |
| ocu-cirR-novel-18038 | 7 | - | 156747849 | 156751096 | ENSOCUT00000010116 | ENSOCUG00000010104 | FN1 | DOWN | -5.86 | 0.0311 |
| ocu-cirR-novel-19076 | 9 | + | 65084466 | 65085519 | ENSOCUT00000009886 | ENSOCUG00000009869 | LAMA3 | DOWN | -2.80 | 0.0320 |
| ocu-cirR-novel-13061 | 14 | - | 4394721 | 4440070 | ENSOCUT00000007175 | ENSOCUG00000007174 | SATB1 | DOWN | -3.67 | 0.0321 |
| ocu-cirR-novel-19354 | X | - | 69449342 | 69452238 | ENSOCUT00000007584 | ENSOCUG00000007579 | ATRX | DOWN | -5.09 | 0.0326 |
| ocu-cirR-novel-14319 | 17 | - | 28879078 | 28884278 | ENSOCUT00000017325 | ENSOCUG00000017326 | TUBGCP4 | DOWN | -5.06 | 0.0328 |
| ocu-cirR-novel-16268 | 2 | + | 95710760 | 95713292 | ENSOCUT00000010564 | ENSOCUG00000010565 | TPCN3 | UP | 4.66 | 0.0330 |
| ocu-cirR-novel-10057 | 1 | - | 113208795 | 113240029 | ENSOCUT00000022604 | ENSOCUG00000016257 | DYNC2H1 | UP | 1.42 | 0.0335 |
| ocu-cirR-novel-14342 | 17 | - | 29624964 | 29629940 | ENSOCUT00000011002 | ENSOCUG00000011004 | HAUS2 | DOWN | -2.55 | 0.0337 |
| ocu-cirR-novel-13500 | 15 | - | 62949886 | 62956489 | ENSOCUT00000013304 | ENSOCUG00000013294 | HELQ | DOWN | -4.82 | 0.0344 |
| ocu-cirR-novel-17860 | 7 | + | 121475965 | 121499876 | ENSOCUT00000028028 | ENSOCUG00000003008 | ITGA4 | DOWN | -5.18 | 0.0345 |
| ocu-cirR-novel-13719 | 16 | - | 10810550 | 10820767 | ENSOCUT00000009863 | ENSOCUG00000009863 | PRKCQ | DOWN | -5.18 | 0.0345 |
| ocu-cirR-novel-14915 | 18 | - | 48192444 | 48193457 | ENSOCUT00000013129 | ENSOCUG00000013130 | CWF19L1 | DOWN | -4.62 | 0.0346 |
| ocu-cirR-novel-10230 | 1 | - | 150896262 | 150907230 | ENSOCUT00000016336 | ENSOCUG00000016329 | DENND5A | UP | 2.59 | 0.0347 |
| ocu-cirR-novel-17728 | 6 | - | 5216182 | 5217852 | ENSOCUT00000017016 | ENSOCUG00000017012 | ZC3H7A | DOWN | -5.72 | 0.0348 |
| ocu-cirR-novel-10143 | 1 | - | 13222294 | 13222537 | ENSOCUT00000004216 | ENSOCUG00000004217 | STX17 | UP | 3.33 | 0.0351 |
| ocu-cirR-novel-16636 | 3 | - | 117745208 | 117747449 | ENSOCUT00000008545 | ENSOCUG00000008545 | RRM2B | DOWN | -5.18 | 0.0351 |
| ocu-cirR-novel-10444 | 1 | - | 26350155 | 26351996 | ENSOCUT00000017669 | ENSOCUG00000017666 | TEK | UP | 5.06 | 0.0354 |
| ocu-cirR-novel-10715 | 1 | + | 8194009 | 8196220 | ENSOCUT00000009288 | ENSOCUG00000009279 | ABCA1 | UP | 2.43 | 0.0355 |
| ocu-cirR-novel-16617 | 3 | + | 114808827 | 114892383 | ENSOCUT00000008743 | ENSOCUG00000008725 | VPS13B | UP | 3.56 | 0.0356 |
| ocu-cirR-novel-15960 | 2 | - | 28369450 | 28378511 | ENSOCUT00000000579 | ENSOCUG00000000579 | TMEM156 | DOWN | -5.08 | 0.0361 |
| ocu-cirR-novel-14389 | 17 | - | 32049543 | 32052084 | ENSOCUT00000006516 | ENSOCUG00000006506 | EIF2AK4 | UP | 2.62 | 0.0362 |
| ocu-cirR-novel-18286 | 7 | + | 75754771 | 75759218 | ENSOCUT00000009063 | ENSOCUG00000009065 | HNMT | UP | 4.74 | 0.0366 |
| ocu-cirR-novel-18122 | 7 | + | 20857644 | 20876054 | ENSOCUT00000008373 | ENSOCUG00000008373 | WASL | DOWN | -3.02 | 0.0370 |
| ocu-cirR-novel-16228 | 2 | - | 90257135 | 90260946 | ENSOCUT00000006957 | ENSOCUG00000006957 | IL1R1 | UP | 2.26 | 0.0371 |
| ocu-cirR-novel-18415 | 8 | - | 14991344 | 14998978 | ENSOCUT00000015967 | ENSOCUG00000015956 | LRMP | DOWN | -3.67 | 0.0373 |
| ocu-cirR-novel-16792 | 3 | + | 15632133 | 15637697 | ENSOCUT00000028451 | ENSOCUG00000025001 | RAD50 | DOWN | 4.81 | 0.0375 |
| ocu-cirR-novel-12331 | 13 | - | 32969251 | 32969706 | ENSOCUT00000025802 | ENSOCUG00000017667 | ATP1A2 | DOWN | -4.98 | 0.0379 |
| ocu-cirR-novel-16618 | 3 | + | 114808827 | 114970635 | ENSOCUT00000008743 | ENSOCUG00000008725 | VPS13B | UP | 5.22 | 0.0380 |
| ocu-cirR-novel-13897 | 16 | + | 3717139 | 3730485 | ENSOCUT00000015957 | ENSOCUG00000015950 | ARHGAP12 | UP | 2.15 | 0.0381 |
| ocu-cirR-novel-10919 | 10 | + | 45078648 | 45082207 | ENSOCUT00000002425 | ENSOCUG00000002419 | OGDH | UP | 2.39 | 0.0383 |
| ocu-cirR-novel-16458 | 20 | + | 9053986 | 9054398 | ENSOCUT00000015862 | ENSOCUG00000015859 | PRKCH | DOWN | -1.61 | 0.0388 |
| ocu-cirR-novel-15158 | 19 | - | 18676792 | 18710446 | ENSOCUT00000015740 | ENSOCUG00000015730 | TAOK1 | DOWN | -3.13 | 0.0391 |
| ocu-cirR-novel-19160 | 9 | - | 90230489 | 90245982 | ENSOCUT00000015855 | ENSOCUG00000015851 | DYM | UP | 2.39 | 0.0392 |
| ocu-cirR-novel-16327 | 20 | + | 13909815 | 13916917 | ENSOCUT00000005686 | ENSOCUG00000005685 | TRIP11 | UP | 4.61 | 0.0404 |
| ocu-cirR-novel-16001 | 2 | - | 31687978 | 31697067 | ENSOCUT00000022300 | ENSOCUG00000013152 | ATP8A1 | DOWN | -2.61 | 0.0406 |
| ocu-cirR-novel-15925 | 2 | - | 173014979 | 173032120 | ENSOCUT00000005722 | ENSOCUG00000005723 | ATAD2B | DOWN | -4.45 | 0.0408 |
| ocu-cirR-novel-11092 | 11 | - | 57398910 | 57418007 | ENSOCUT00000009161 | ENSOCUG00000009162 | NADK2 | DOWN | -4.90 | 0.0412 |
| ocu-cirR-novel-16819 | 3 | + | 17406890 | 17415253 | ENSOCUT00000008262 | ENSOCUG00000008254 | SEC24A | DOWN | -2.85 | 0.0422 |
| ocu-cirR-novel-12697 | 13 | + | 99347865 | 99389617 | ENSOCUT00000031843 | ENSOCUG00000021747 | WDR78 | DOWN | -2.97 | 0.0426 |
| ocu-cirR-novel-15740 | 2 | - | 142033506 | 142041910 | ENSOCUT00000005459 | ENSOCUG00000005461 | PPM1B | DOWN | -1.50 | 0.0432 |
| ocu-cirR-novel-15638 | 2 | + | 124771148 | 124772410 | ENSOCUT00000004989 | ENSOCUG00000004986 | XPO1 | DOWN | -1.52 | 0.0438 |
| ocu-cirR-novel-14330 | 17 | - | 29117357 | 29120353 | ENSOCUT00000022131 | ENSOCUG00000017449 | TMEM62 | DOWN | -1.58 | 0.0440 |
| ocu-cirR-novel-12487 | 13 | + | 56410147 | 56429422 | ENSOCUT00000000172 | ENSOCUG00000000172 | VAV3 | DOWN | -5.51 | 0.0446 |
| ocu-cirR-novel-16256 | 2 | + | 93478539 | 93480933 | ENSOCUT00000003385 | ENSOCUG00000003383 | MGAT4A | DOWN | -4.89 | 0.0446 |
| ocu-cirR-novel-12538 | 13 | - | 6878445 | 6883717 | ENSOCUT00000016451 | ENSOCUG00000016453 | TOR1AIP2 | UP | 1.83 | 0.0447 |
| ocu-cirR-novel-11377 | 12 | - | 121826483 | 121833777 | ENSOCUT00000001817 | ENSOCUG00000001817 | MED23 | DOWN | -4.70 | 0.0454 |
| ocu-cirR-novel-16006 | 2 | + | 33444476 | 33459816 | ENSOCUT00000017915 | ENSOCUG00000017915 | GUF1 | UP | 2.59 | 0.0463 |
| ocu-cirR-novel-16351 | 20 | + | 16066403 | 16067933 | ENSOCUT00000004393 | ENSOCUG00000004392 | FOXN3 | DOWN | -2.16 | 0.0464 |
| ocu-cirR-novel-17758 | 7 | - | 101162791 | 101164590 | ENSOCUT00000011454 | ENSOCUG00000011445 | DPP4 | DOWN | -2.39 | 0.0466 |
| ocu-cirR-novel-18121 | 7 | - | 204282 | 205756 | ENSOCUT00000005642 | ENSOCUG00000005642 | MTPN | DOWN | -5.02 | 0.0467 |
| ocu-cirR-novel-11500 | 12 | + | 145053078 | 145061676 | ENSOCUT00000023944 | ENSOCUG00000026063 | SCAF8 | DOWN | -3.23 | 0.0470 |
| ocu-cirR-novel-19262 | X | + | 31964180 | 31964846 | ENSOCUT00000005647 | ENSOCUG00000005647 | RP2 | UP | 4.56 | 0.0481 |
| ocu-cirR-novel-17488 | 4 | - | 81075228 | 81084834 | ENSOCUT00000009301 | ENSOCUG00000009299 | GNPTAB | UP | 4.56 | 0.0481 |
| ocu-cirR-novel-14538 | 17 | - | 64570437 | 64585396 | ENSOCUT00000007557 | ENSOCUG00000007550 | MIS18BP1 | DOWN | -2.57 | 0.0487 |
| ocu-cirR-novel-13094 | 14 | + | 50130953 | 50138310 | ENSOCUT00000001481 | ENSOCUG00000001480 | GMPS | DOWN | -4.51 | 0.0490 |
| ocu-cirR-novel-14960 | 18 | + | 51928186 | 51934232 | ENSOCUT00000003039 | ENSOCUG00000003035 | SLK | DOWN | -2.94 | 0.0493 |
