# Supplementary material for: Comprehensive analysis of circRNA expression pattern and circRNA-miRNA-mRNA network in the pathogenesis of atherosclerosis in rabbits
Source: Aging (Albany NY). 2018 Sep 6;10(9):2266–83. doi: 10.18632/aging.101541 (PMC6188486; doi:10.18632/aging.101541)
Supplement: Supplementary Table S3 [file aging-10-101541-s003.docx]

**Supplementary Table S3. Differential expressed miRNA identified by edgeR package.**

| **MiRNA** | **Status** | **logFC** | **PValue** |
| --- | --- | --- | --- |
| ocu-miR-12092-5p | UP | 4.48 | 1.45E-07 |
| ocu-miR-196b-5p | DOWN | -6.33 | 5.61E-06 |
| ocu-miR-424-5p | UP | 3.33 | 1.39E-05 |
| ocu-miR-450a-5p | UP | 3.24 | 3.91E-05 |
| ocu-miR-542-3p | UP | 3.28 | 4.72E-05 |
| ocu-miR-204-5p | DOWN | -2.60 | 7.37E-05 |
| ocu-miR-128b-3p | DOWN | -3.56 | 7.88E-05 |
| ocu-miR-128a-3p | DOWN | -3.56 | 9.74E-05 |
| ocu-miR-21-5p | UP | 2.23 | 0.0001 |
| ocu-miR-34a-5p | UP | 2.45 | 0.0002 |
| ocu-miR-12092-3p | UP | 3.50 | 0.0003 |
| ocu-miR-424-3p | UP | 2.61 | 0.0003 |
| ocu-miR-889-3p | UP | 2.21 | 0.0004 |
| ocu-miR-335-5p | UP | 2.35 | 0.0004 |
| ocu-miR-672-5p | DOWN | -3.20 | 0.0004 |
| ocu-miR-25-3p | DOWN | -2.14 | 0.0005 |
| ocu-miR-199a-5p | UP | 1.85 | 0.0006 |
| ocu-miR-411-5p | UP | 2.22 | 0.0006 |
| ocu-miR-200c-3p | DOWN | -4.57 | 0.0006 |
| ocu-miR-196a-5p | DOWN | -4.50 | 0.0007 |
| ocu-miR-141-3p | DOWN | -4.91 | 0.0007 |
| ocu-miR-182-5p | DOWN | -2.61 | 0.0007 |
| ocu-miR-503-3p | UP | 5.34 | 0.0010 |
| ocu-miR-205-5p | DOWN | -5.67 | 0.0011 |
| ocu-miR-487a-3p | UP | 2.67 | 0.0011 |
| ocu-miR-127-5p | UP | 2.11 | 0.0012 |
| ocu-miR-122-5p | UP | 5.28 | 0.0015 |
| ocu-miR-214-3p | UP | 1.88 | 0.0021 |
| ocu-miR-1-3p | DOWN | -1.95 | 0.0022 |
| ocu-miR-183-5p | DOWN | -2.33 | 0.0024 |
| ocu-miR-377-3p | UP | 2.52 | 0.0027 |
| ocu-miR-450a-2-3p | UP | 2.66 | 0.0028 |
| ocu-miR-214-5p | UP | 1.94 | 0.0030 |
| ocu-miR-190a-5p | DOWN | -2.34 | 0.0030 |
| ocu-miR-15b-3p | DOWN | -2.14 | 0.0031 |
| ocu-miR-136-3p | UP | 1.88 | 0.0031 |
| ocu-miR-451-5p | DOWN | -2.17 | 0.0041 |
| ocu-miR-130b-3p | DOWN | -3.28 | 0.0042 |
| ocu-miR-450b-5p | UP | 2.58 | 0.0042 |
| ocu-miR-92a-3p | DOWN | -1.90 | 0.0043 |
| ocu-miR-96-5p | DOWN | -2.27 | 0.0045 |
| ocu-miR-655-3p | UP | 1.79 | 0.0046 |
| ocu-miR-539-3p | UP | 2.18 | 0.0047 |
| ocu-miR-432-5p | UP | 2.00 | 0.0047 |
| ocu-miR-363-5p | DOWN | -2.82 | 0.0049 |
| ocu-miR-299-3p | UP | 1.73 | 0.0050 |
| ocu-miR-154-5p | UP | 1.97 | 0.0053 |
| ocu-miR-135a-5p | DOWN | -2.23 | 0.0056 |
| ocu-miR-656-3p | UP | 1.79 | 0.0057 |
| ocu-miR-486-5p | DOWN | -1.92 | 0.0057 |
| ocu-miR-93-5p | DOWN | -1.71 | 0.0067 |
| ocu-miR-449a-5p | UP | 1.84 | 0.0083 |
| ocu-miR-181a-3p | DOWN | -2.63 | 0.0083 |
| ocu-miR-363-3p | DOWN | -2.02 | 0.0085 |
| ocu-miR-21-3p | UP | 3.07 | 0.0089 |
| ocu-miR-7a-5p | DOWN | -1.54 | 0.0089 |
| ocu-miR-199a-3p | UP | 1.41 | 0.0091 |
| ocu-miR-154-3p | UP | 1.91 | 0.0094 |
| ocu-miR-539-5p | UP | 1.94 | 0.0103 |
| ocu-miR-134-5p | UP | 1.84 | 0.0113 |
| ocu-miR-376a-3p | UP | 1.80 | 0.0120 |
| ocu-miR-409-5p | UP | 1.57 | 0.0123 |
| ocu-miR-215-5p | DOWN | -2.21 | 0.0129 |
| ocu-miR-143-3p | DOWN | -1.51 | 0.0130 |
| ocu-miR-141-5p | DOWN | -3.71 | 0.0132 |
| ocu-miR-487b-3p | UP | 1.64 | 0.0132 |
| ocu-miR-142-5p | DOWN | -2.19 | 0.0141 |
| ocu-miR-20b-5p | DOWN | -2.07 | 0.0146 |
| ocu-miR-497-5p | UP | 1.60 | 0.0148 |
| ocu-miR-18a-3p | DOWN | -2.03 | 0.0149 |
| ocu-miR-450a-3p | UP | 2.57 | 0.0160 |
| ocu-miR-29a-5p | UP | 2.20 | 0.0160 |
| ocu-miR-145-3p | DOWN | -1.41 | 0.0166 |
| ocu-miR-503-5p | UP | 2.11 | 0.0166 |
| ocu-miR-106b-3p | DOWN | -1.64 | 0.0170 |
| ocu-miR-541-5p | UP | 2.50 | 0.0173 |
| ocu-miR-106a-5p | DOWN | -1.97 | 0.0177 |
| ocu-miR-20a-5p | DOWN | -1.66 | 0.0191 |
| ocu-miR-15b-5p | DOWN | -1.78 | 0.0192 |
| ocu-miR-370-3p | UP | 1.49 | 0.0199 |
| ocu-miR-383-5p | DOWN | -1.62 | 0.0199 |
| ocu-miR-181b-3p | DOWN | -2.69 | 0.0200 |
| ocu-miR-33a-5p | DOWN | -3.39 | 0.0211 |
| ocu-miR-142-3p | DOWN | -2.33 | 0.0214 |
| ocu-miR-130b-5p | DOWN | -2.42 | 0.0215 |
| ocu-miR-136-5p | UP | 1.79 | 0.0219 |
| ocu-miR-181b-5p | DOWN | -1.85 | 0.0223 |
| ocu-miR-410-3p | UP | 1.58 | 0.0235 |
| ocu-miR-302a-5p | DOWN | -2.64 | 0.0241 |
| ocu-miR-145-5p | DOWN | -1.54 | 0.0267 |
| ocu-miR-342-3p | DOWN | -1.85 | 0.0267 |
| ocu-miR-211-5p | UP | 1.62 | 0.0279 |
| ocu-miR-411-3p | UP | 1.49 | 0.0282 |
| ocu-miR-19b-3p | DOWN | -1.64 | 0.0310 |
| ocu-miR-204-3p | DOWN | -1.44 | 0.0318 |
| ocu-miR-190b-5p | DOWN | -2.58 | 0.0324 |
| ocu-miR-377-5p | UP | 2.31 | 0.0334 |
| ocu-miR-7a-3p | DOWN | -1.41 | 0.0334 |
| ocu-miR-181d-3p | UP | 2.43 | 0.0334 |
| ocu-miR-671-3p | DOWN | -1.67 | 0.0339 |
| ocu-miR-337-5p | UP | 2.14 | 0.0347 |
| ocu-miR-1298-5p | DOWN | -1.74 | 0.0348 |
| ocu-miR-31-3p | UP | 1.82 | 0.0363 |
| ocu-miR-181a-5p | DOWN | -1.59 | 0.0364 |
| ocu-miR-181a-2-3p | DOWN | -1.63 | 0.0385 |
| ocu-miR-16b-3p | DOWN | -1.65 | 0.0387 |
| ocu-miR-382-3p | UP | 1.38 | 0.0398 |
| ocu-miR-17-5p | DOWN | -1.58 | 0.0419 |
| ocu-miR-455-3p | UP | 1.34 | 0.0425 |
| ocu-miR-133a-5p | DOWN | -1.55 | 0.0431 |
| ocu-miR-299-5p | UP | 1.48 | 0.0438 |
| ocu-miR-22-5p | UP | 1.20 | 0.0471 |
| ocu-miR-194-5p | DOWN | -1.53 | 0.0475 |
