# Supplementary material for: Comprehensive analysis of circRNA expression pattern and circRNA-miRNA-mRNA network in the pathogenesis of atherosclerosis in rabbits
Source: Aging (Albany NY). 2018 Sep 6;10(9):2266–83. doi: 10.18632/aging.101541 (PMC6188486; doi:10.18632/aging.101541)
Supplement: Supplementary Table S4 [file aging-10-101541-s004.docx]

**Supplementary Table S4. CircRNA-mRNA interaction pairs based on hypergeometric test.**

| **CircRNA** | **Gene_id** | **Gene_name** | **Pvalue** |
| --- | --- | --- | --- |
| ocu-cirR-novel-10057 | ENSOCUG00000003569 | HMGCS1 | 0.0247 |
| ocu-cirR-novel-10057 | ENSOCUG00000016005 | EXO5 | 0.0123 |
| ocu-cirR-novel-10057 | ENSOCUG00000023764 | CCDC80 | 0.0494 |
| ocu-cirR-novel-10065 | ENSOCUG00000000114 | DUSP2 | 0.0491 |
| ocu-cirR-novel-10065 | ENSOCUG00000004136 | SEPT-7 | 0.0111 |
| ocu-cirR-novel-10065 | ENSOCUG00000016580 | CTSB | 0.0139 |
| ocu-cirR-novel-10065 | ENSOCUG00000017700 | PLS1 | 0.0247 |
| ocu-cirR-novel-10230 | ENSOCUG00000001858 | ICOS | 0.0087 |
| ocu-cirR-novel-10230 | ENSOCUG00000003850 | PTCRA | 0.0471 |
| ocu-cirR-novel-10230 | ENSOCUG00000003999 | CAMK1G | 0.0304 |
| ocu-cirR-novel-10230 | ENSOCUG00000005294 | RRAGD | 0.0165 |
| ocu-cirR-novel-10230 | ENSOCUG00000006910 | CDCP1 | 0.0125 |
| ocu-cirR-novel-10230 | ENSOCUG00000009585 | SRC | 0.0165 |
| ocu-cirR-novel-10230 | ENSOCUG00000009828 | ARPP21 | 0.0471 |
| ocu-cirR-novel-10230 | ENSOCUG00000009863 | PRKCQ | 0.0381 |
| ocu-cirR-novel-10230 | ENSOCUG00000009918 | SCARA3 | 0.0304 |
| ocu-cirR-novel-10230 | ENSOCUG00000011267 | ARHGEF9 | 0.0017 |
| ocu-cirR-novel-10230 | ENSOCUG00000011550 | CD86 | 0.0071 |
| ocu-cirR-novel-10230 | ENSOCUG00000013625 | PRSS12 | 0.0471 |
| ocu-cirR-novel-10230 | ENSOCUG00000014812 | CD93 | 0.0165 |
| ocu-cirR-novel-10230 | ENSOCUG00000024349 | LOC100337909 | 0.0087 |
| ocu-cirR-novel-10230 | ENSOCUG00000024777 | . | 0.0165 |
| ocu-cirR-novel-10230 | ENSOCUG00000025121 | ALDH6A1 | 0.0087 |
| ocu-cirR-novel-10230 | ENSOCUG00000026691 | SLC31A2 | 0.0381 |
| ocu-cirR-novel-10714 | ENSOCUG00000003299 | DMRT2 | 0.0090 |
| ocu-cirR-novel-10714 | ENSOCUG00000006800 | ATP10B | 0.0004 |
| ocu-cirR-novel-10714 | ENSOCUG00000009918 | SCARA3 | 0.0134 |
| ocu-cirR-novel-10714 | ENSOCUG00000015482 | TMEM241 | 0.0053 |
| ocu-cirR-novel-10714 | ENSOCUG00000025244 | IGFBP7 | 0.0370 |
| ocu-cirR-novel-10714 | ENSOCUG00000026988 | CHI3L1 | 0.0471 |
| ocu-cirR-novel-10714 | ENSOCUG00000028052 | AMOTL2 | 0.0389 |
| ocu-cirR-novel-10715 | ENSOCUG00000003299 | DMRT2 | 0.0090 |
| ocu-cirR-novel-10715 | ENSOCUG00000006800 | ATP10B | 0.0004 |
| ocu-cirR-novel-10715 | ENSOCUG00000009918 | SCARA3 | 0.0134 |
| ocu-cirR-novel-10715 | ENSOCUG00000015482 | TMEM241 | 0.0053 |
| ocu-cirR-novel-10715 | ENSOCUG00000025244 | IGFBP7 | 0.0370 |
| ocu-cirR-novel-10715 | ENSOCUG00000026988 | CHI3L1 | 0.0471 |
| ocu-cirR-novel-10715 | ENSOCUG00000028052 | AMOTL2 | 0.0389 |
| ocu-cirR-novel-10858 | ENSOCUG00000001542 | RIPK2 | 0.0494 |
| ocu-cirR-novel-10858 | ENSOCUG00000001590 | KLC4 | 0.0259 |
| ocu-cirR-novel-10858 | ENSOCUG00000003792 | THBS1 | 0.0467 |
| ocu-cirR-novel-10858 | ENSOCUG00000004337 | SMARCD2 | 0.0384 |
| ocu-cirR-novel-10858 | ENSOCUG00000005891 | ELAVL2 | 0.0107 |
| ocu-cirR-novel-10858 | ENSOCUG00000006752 | MEST | 0.0384 |
| ocu-cirR-novel-10858 | ENSOCUG00000007145 | KIAA0586 | 0.0019 |
| ocu-cirR-novel-10858 | ENSOCUG00000008167 | CALU | 0.0025 |
| ocu-cirR-novel-10858 | ENSOCUG00000009866 | ANKRD29 | 0.0276 |
| ocu-cirR-novel-10858 | ENSOCUG00000009869 | LAMA3 | 0.0357 |
| ocu-cirR-novel-10858 | ENSOCUG00000010772 | DCHS2 | 0.0467 |
| ocu-cirR-novel-10858 | ENSOCUG00000011937 | KIF3A | 0.0467 |
| ocu-cirR-novel-10858 | ENSOCUG00000012578 | CAMK2G | 0.0467 |
| ocu-cirR-novel-10858 | ENSOCUG00000015897 | ASF1A | 0.0005 |
| ocu-cirR-novel-10858 | ENSOCUG00000017386 | RPL30 | 0.0494 |
| ocu-cirR-novel-10858 | ENSOCUG00000017498 | . | 0.0176 |
| ocu-cirR-novel-10858 | ENSOCUG00000017821 | GATA3 | 0.0176 |
| ocu-cirR-novel-10919 | ENSOCUG00000001142 | SNX13 | 0.0491 |
| ocu-cirR-novel-10919 | ENSOCUG00000001165 | RANBP2 | 0.0491 |
| ocu-cirR-novel-10919 | ENSOCUG00000002623 | ESAM | 0.0170 |
| ocu-cirR-novel-10919 | ENSOCUG00000006320 | SMARCA2 | 0.0491 |
| ocu-cirR-novel-10919 | ENSOCUG00000010095 | ZNF18 | 0.0324 |
| ocu-cirR-novel-10919 | ENSOCUG00000010440 | MSI2 | 0.0281 |
| ocu-cirR-novel-10919 | ENSOCUG00000013078 | FAXDC2 | 0.0472 |
| ocu-cirR-novel-10919 | ENSOCUG00000013961 | DNAH11 | 0.0247 |
| ocu-cirR-novel-10919 | ENSOCUG00000015482 | TMEM241 | 0.0324 |
| ocu-cirR-novel-10919 | ENSOCUG00000024081 | CD63 | 0.0491 |
| ocu-cirR-novel-10919 | ENSOCUG00000024139 | TRA2A | 0.0247 |
| ocu-cirR-novel-10985 | ENSOCUG00000004293 | EPS15 | 0.0247 |
| ocu-cirR-novel-10985 | ENSOCUG00000005822 | MROH8 | 0.0494 |
| ocu-cirR-novel-10985 | ENSOCUG00000009402 | USP32 | 0.0494 |
| ocu-cirR-novel-10985 | ENSOCUG00000011085 | CTSS | 0.0370 |
| ocu-cirR-novel-10985 | ENSOCUG00000014338 | ABCA5 | 0.0494 |
| ocu-cirR-novel-10985 | ENSOCUG00000022021 | IL22RA2 | 0.0494 |
| ocu-cirR-novel-10997 | ENSOCUG00000004632 | CA12 | 0.0420 |
| ocu-cirR-novel-10997 | ENSOCUG00000006910 | CDCP1 | 0.0472 |
| ocu-cirR-novel-10997 | ENSOCUG00000008167 | CALU | 0.0086 |
| ocu-cirR-novel-10997 | ENSOCUG00000008568 | ANKRD28 | 0.0241 |
| ocu-cirR-novel-10997 | ENSOCUG00000009547 | MYO5B | 0.0491 |
| ocu-cirR-novel-10997 | ENSOCUG00000026567 | RLA-DMB | 0.0491 |
| ocu-cirR-novel-10998 | ENSOCUG00000004632 | CA12 | 0.0420 |
| ocu-cirR-novel-10998 | ENSOCUG00000006910 | CDCP1 | 0.0472 |
| ocu-cirR-novel-10998 | ENSOCUG00000008167 | CALU | 0.0086 |
| ocu-cirR-novel-10998 | ENSOCUG00000008568 | ANKRD28 | 0.0241 |
| ocu-cirR-novel-10998 | ENSOCUG00000009547 | MYO5B | 0.0491 |
| ocu-cirR-novel-10998 | ENSOCUG00000026567 | RLA-DMB | 0.0491 |
| ocu-cirR-novel-11092 | ENSOCUG00000001414 | DDX23 | 0.0055 |
| ocu-cirR-novel-11092 | ENSOCUG00000001542 | RIPK2 | 0.0370 |
| ocu-cirR-novel-11092 | ENSOCUG00000003186 | HPRT1 | 0.0053 |
| ocu-cirR-novel-11092 | ENSOCUG00000007702 | TBC1D10C | 0.0389 |
| ocu-cirR-novel-11092 | ENSOCUG00000008210 | CCR9 | 0.0314 |
| ocu-cirR-novel-11092 | ENSOCUG00000008983 | CTCF | 0.0471 |
| ocu-cirR-novel-11092 | ENSOCUG00000017700 | PLS1 | 0.0370 |
| ocu-cirR-novel-11092 | ENSOCUG00000024358 | CDIPT | 0.0471 |
| ocu-cirR-novel-11092 | ENSOCUG00000026630 | ITGA9 | 0.0314 |
| ocu-cirR-novel-11169 | ENSOCUG00000001250 | EDNRA | 0.0139 |
| ocu-cirR-novel-11169 | ENSOCUG00000003089 | PRG3 | 0.0247 |
| ocu-cirR-novel-11169 | ENSOCUG00000003276 | PAX1 | 0.0491 |
| ocu-cirR-novel-11169 | ENSOCUG00000003792 | THBS1 | 0.0086 |
| ocu-cirR-novel-11169 | ENSOCUG00000003863 | CD74 | 0.0491 |
| ocu-cirR-novel-11169 | ENSOCUG00000005439 | CCNJL | 0.0281 |
| ocu-cirR-novel-11169 | ENSOCUG00000006320 | SMARCA2 | 0.0491 |
| ocu-cirR-novel-11169 | ENSOCUG00000006970 | SLC44A4 | 0.0247 |
| ocu-cirR-novel-11169 | ENSOCUG00000009047 | FCRL2 | 0.0491 |
| ocu-cirR-novel-11169 | ENSOCUG00000010247 | ATP6V0D2 | 0.0491 |
| ocu-cirR-novel-11169 | ENSOCUG00000010633 | HMOX1 | 0.0491 |
| ocu-cirR-novel-11169 | ENSOCUG00000015599 | GEMIN6 | 0.0491 |
| ocu-cirR-novel-11169 | ENSOCUG00000016500 | . | 0.0491 |
| ocu-cirR-novel-11169 | ENSOCUG00000025091 | AARD | 0.0491 |
| ocu-cirR-novel-11221 | ENSOCUG00000009190 | HEXA | 0.0491 |
| ocu-cirR-novel-11221 | ENSOCUG00000013137 | CETP | 0.0491 |
| ocu-cirR-novel-11221 | ENSOCUG00000013593 | BPI | 0.0247 |
| ocu-cirR-novel-11221 | ENSOCUG00000015431 | DOCK10 | 0.0247 |
| ocu-cirR-novel-11221 | ENSOCUG00000016500 | . | 0.0491 |
| ocu-cirR-novel-11291 | ENSOCUG00000000788 | GADL1 | 0.0494 |
| ocu-cirR-novel-11291 | ENSOCUG00000001387 | TDRKH | 0.0494 |
| ocu-cirR-novel-11291 | ENSOCUG00000009322 | MCOLN2 | 0.0494 |
| ocu-cirR-novel-11291 | ENSOCUG00000009680 | HOMER1 | 0.0247 |
| ocu-cirR-novel-11291 | ENSOCUG00000023836 | NEFL | 0.0370 |
| ocu-cirR-novel-11377 | ENSOCUG00000000268 | PCDH15 | 0.0408 |
| ocu-cirR-novel-11377 | ENSOCUG00000001553 | LIPA | 0.0467 |
| ocu-cirR-novel-11377 | ENSOCUG00000002871 | DNTT | 0.0113 |
| ocu-cirR-novel-11377 | ENSOCUG00000003792 | THBS1 | 0.0286 |
| ocu-cirR-novel-11377 | ENSOCUG00000005012 | SH2D1A | 0.0246 |
| ocu-cirR-novel-11377 | ENSOCUG00000005891 | ELAVL2 | 0.0467 |
| ocu-cirR-novel-11377 | ENSOCUG00000006632 | MREG | 0.0408 |
| ocu-cirR-novel-11377 | ENSOCUG00000009701 | CSF3 | 0.0467 |
| ocu-cirR-novel-11377 | ENSOCUG00000010247 | ATP6V0D2 | 0.0086 |
| ocu-cirR-novel-11377 | ENSOCUG00000010633 | HMOX1 | 0.0086 |
| ocu-cirR-novel-11377 | ENSOCUG00000011739 | . | 0.0408 |
| ocu-cirR-novel-11377 | ENSOCUG00000016465 | TP63 | 0.0467 |
| ocu-cirR-novel-11377 | ENSOCUG00000016706 | KIF11 | 0.0246 |
| ocu-cirR-novel-11377 | ENSOCUG00000017747 | CPNE8 | 0.0059 |
| ocu-cirR-novel-11377 | ENSOCUG00000021242 | PKP1 | 0.0188 |
| ocu-cirR-novel-11377 | ENSOCUG00000024358 | CDIPT | 0.0102 |
| ocu-cirR-novel-11377 | ENSOCUG00000029399 | SH2D1B | 0.0102 |
| ocu-cirR-novel-11489 | ENSOCUG00000002555 | SCP2 | 0.0389 |
| ocu-cirR-novel-11489 | ENSOCUG00000002623 | ESAM | 0.0471 |
| ocu-cirR-novel-11489 | ENSOCUG00000002624 | NRK | 0.0186 |
| ocu-cirR-novel-11489 | ENSOCUG00000003031 | GGH | 0.0090 |
| ocu-cirR-novel-11489 | ENSOCUG00000005603 | TESPA1 | 0.0246 |
| ocu-cirR-novel-11489 | ENSOCUG00000005655 | SMC2 | 0.0246 |
| ocu-cirR-novel-11489 | ENSOCUG00000006337 | TRH | 0.0090 |
| ocu-cirR-novel-11489 | ENSOCUG00000008346 | LYPD6B | 0.0028 |
| ocu-cirR-novel-11489 | ENSOCUG00000008474 | CD2 | 0.0134 |
| ocu-cirR-novel-11489 | ENSOCUG00000009077 | MS4A2 | 0.0009 |
| ocu-cirR-novel-11489 | ENSOCUG00000009311 | CD84 | 0.0246 |
| ocu-cirR-novel-11489 | ENSOCUG00000009882 | NCAPH | 0.0028 |
| ocu-cirR-novel-11489 | ENSOCUG00000010261 | EHF | 0.0010 |
| ocu-cirR-novel-11489 | ENSOCUG00000011064 | CLSTN1 | 0.0080 |
| ocu-cirR-novel-11489 | ENSOCUG00000012455 | MDFIC | 0.0004 |
| ocu-cirR-novel-11489 | ENSOCUG00000015020 | COL5A2 | 0.0186 |
| ocu-cirR-novel-11489 | ENSOCUG00000016254 | STAB2 | 0.0026 |
| ocu-cirR-novel-11489 | ENSOCUG00000016268 | RCN1 | 0.0080 |
| ocu-cirR-novel-11489 | ENSOCUG00000016959 | CRYBG2 | 0.0471 |
| ocu-cirR-novel-11489 | ENSOCUG00000017023 | ATF1 | 0.0314 |
| ocu-cirR-novel-11489 | ENSOCUG00000017438 | SELE | 0.0090 |
| ocu-cirR-novel-11489 | ENSOCUG00000017563 | AKAP1 | 0.0134 |
| ocu-cirR-novel-11489 | ENSOCUG00000023078 | . | 0.0053 |
| ocu-cirR-novel-11489 | ENSOCUG00000023764 | CCDC80 | 0.0055 |
| ocu-cirR-novel-11489 | ENSOCUG00000025244 | IGFBP7 | 0.0370 |
| ocu-cirR-novel-11489 | ENSOCUG00000025830 | CAPN5 | 0.0471 |
| ocu-cirR-novel-11489 | ENSOCUG00000027603 | PDGFB | 0.0343 |
| ocu-cirR-novel-11489 | ENSOCUG00000029154 | . | 0.0080 |
| ocu-cirR-novel-11489 | ENSOCUG00000029742 | MAP1LC3C | 0.0389 |
| ocu-cirR-novel-11500 | ENSOCUG00000001165 | RANBP2 | 0.0491 |
| ocu-cirR-novel-11500 | ENSOCUG00000001387 | TDRKH | 0.0019 |
| ocu-cirR-novel-11500 | ENSOCUG00000002528 | LRCH4 | 0.0491 |
| ocu-cirR-novel-11500 | ENSOCUG00000002898 | FBLN7 | 0.0281 |
| ocu-cirR-novel-11500 | ENSOCUG00000009680 | HOMER1 | 0.0491 |
| ocu-cirR-novel-11500 | ENSOCUG00000009929 | C3orf52 | 0.0139 |
| ocu-cirR-novel-11500 | ENSOCUG00000025590 | LAT | 0.0491 |
| ocu-cirR-novel-11500 | ENSOCUG00000026206 | CCL4 | 0.0491 |
| ocu-cirR-novel-11500 | ENSOCUG00000029399 | SH2D1B | 0.0170 |
| ocu-cirR-novel-11502 | ENSOCUG00000001165 | RANBP2 | 0.0491 |
| ocu-cirR-novel-11502 | ENSOCUG00000001387 | TDRKH | 0.0019 |
| ocu-cirR-novel-11502 | ENSOCUG00000002528 | LRCH4 | 0.0491 |
| ocu-cirR-novel-11502 | ENSOCUG00000002898 | FBLN7 | 0.0281 |
| ocu-cirR-novel-11502 | ENSOCUG00000009680 | HOMER1 | 0.0491 |
| ocu-cirR-novel-11502 | ENSOCUG00000009929 | C3orf52 | 0.0139 |
| ocu-cirR-novel-11502 | ENSOCUG00000025590 | LAT | 0.0491 |
| ocu-cirR-novel-11502 | ENSOCUG00000026206 | CCL4 | 0.0491 |
| ocu-cirR-novel-11502 | ENSOCUG00000029399 | SH2D1B | 0.0170 |
| ocu-cirR-novel-11533 | ENSOCUG00000001387 | TDRKH | 0.0467 |
| ocu-cirR-novel-11533 | ENSOCUG00000003186 | HPRT1 | 0.0046 |
| ocu-cirR-novel-11533 | ENSOCUG00000004847 | GAP43 | 0.0086 |
| ocu-cirR-novel-11533 | ENSOCUG00000005094 | INHBB | 0.0246 |
| ocu-cirR-novel-11533 | ENSOCUG00000006186 | CPXM1 | 0.0286 |
| ocu-cirR-novel-11533 | ENSOCUG00000006350 | SLC12A4 | 0.0246 |
| ocu-cirR-novel-11533 | ENSOCUG00000006595 | UBA7 | 0.0202 |
| ocu-cirR-novel-11533 | ENSOCUG00000006694 | TNLG1E | 0.0007 |
| ocu-cirR-novel-11533 | ENSOCUG00000007702 | TBC1D10C | 0.0068 |
| ocu-cirR-novel-11533 | ENSOCUG00000009241 | RAB11FIP2 | 0.0352 |
| ocu-cirR-novel-11533 | ENSOCUG00000010061 | PLEKHG4 | 0.0286 |
| ocu-cirR-novel-11533 | ENSOCUG00000010671 | SH2D2A | 0.0059 |
| ocu-cirR-novel-11533 | ENSOCUG00000010772 | DCHS2 | 0.0286 |
| ocu-cirR-novel-11533 | ENSOCUG00000010972 | LOC100328967 | 0.0059 |
| ocu-cirR-novel-11533 | ENSOCUG00000011064 | CLSTN1 | 0.0088 |
| ocu-cirR-novel-11533 | ENSOCUG00000013333 | HS3ST3A1 | 0.0202 |
| ocu-cirR-novel-11533 | ENSOCUG00000013567 | GRN | 0.0025 |
| ocu-cirR-novel-11533 | ENSOCUG00000016706 | KIF11 | 0.0246 |
| ocu-cirR-novel-11533 | ENSOCUG00000016727 | NELL2 | 0.0188 |
| ocu-cirR-novel-11533 | ENSOCUG00000021580 | FGF23 | 0.0188 |
| ocu-cirR-novel-11533 | ENSOCUG00000022391 | . | 0.0467 |
| ocu-cirR-novel-11533 | ENSOCUG00000022519 | MXD3 | 0.0056 |
| ocu-cirR-novel-11533 | ENSOCUG00000023078 | . | 0.0046 |
| ocu-cirR-novel-11533 | ENSOCUG00000025569 | CCR7 | 0.0467 |
| ocu-cirR-novel-11533 | ENSOCUG00000026206 | CCL4 | 0.0086 |
| ocu-cirR-novel-11533 | ENSOCUG00000026630 | ITGA9 | 0.0043 |
| ocu-cirR-novel-11651 | ENSOCUG00000006350 | SLC12A4 | 0.0370 |
| ocu-cirR-novel-11832 | ENSOCUG00000001316 | ESRP1 | 0.0370 |
| ocu-cirR-novel-11832 | ENSOCUG00000001414 | DDX23 | 0.0494 |
| ocu-cirR-novel-11832 | ENSOCUG00000003165 | SCUBE2 | 0.0370 |
| ocu-cirR-novel-11832 | ENSOCUG00000005012 | SH2D1A | 0.0370 |
| ocu-cirR-novel-11832 | ENSOCUG00000005094 | INHBB | 0.0370 |
| ocu-cirR-novel-11832 | ENSOCUG00000005729 | RIN3 | 0.0494 |
| ocu-cirR-novel-11832 | ENSOCUG00000005937 | SCD5 | 0.0247 |
| ocu-cirR-novel-11832 | ENSOCUG00000009195 | CLEC4E | 0.0494 |
| ocu-cirR-novel-11832 | ENSOCUG00000009701 | CSF3 | 0.0494 |
| ocu-cirR-novel-11832 | ENSOCUG00000009932 | ESCO2 | 0.0370 |
| ocu-cirR-novel-11832 | ENSOCUG00000010203 | TLN1 | 0.0370 |
| ocu-cirR-novel-11832 | ENSOCUG00000010247 | ATP6V0D2 | 0.0247 |
| ocu-cirR-novel-11832 | ENSOCUG00000014988 | COL3A1 | 0.0123 |
| ocu-cirR-novel-11832 | ENSOCUG00000015138 | C1QC | 0.0494 |
| ocu-cirR-novel-11832 | ENSOCUG00000025512 | SPINK8 | 0.0123 |
| ocu-cirR-novel-11832 | ENSOCUG00000025590 | LAT | 0.0247 |
| ocu-cirR-novel-11832 | ENSOCUG00000026887 | . | 0.0494 |
| ocu-cirR-novel-11832 | ENSOCUG00000027275 | TM4SF1 | 0.0494 |
| ocu-cirR-novel-11832 | ENSOCUG00000029569 | KRT17 | 0.0123 |
| ocu-cirR-novel-12026 | ENSOCUG00000001144 | SLC6A6 | 0.0314 |
| ocu-cirR-novel-12026 | ENSOCUG00000003031 | GGH | 0.0090 |
| ocu-cirR-novel-12026 | ENSOCUG00000004035 | IRF6 | 0.0389 |
| ocu-cirR-novel-12026 | ENSOCUG00000004169 | ASAH1 | 0.0090 |
| ocu-cirR-novel-12026 | ENSOCUG00000006561 | XAF1 | 0.0009 |
| ocu-cirR-novel-12026 | ENSOCUG00000007265 | TREM2 | 0.0370 |
| ocu-cirR-novel-12026 | ENSOCUG00000007765 | PSAT1 | 0.0028 |
| ocu-cirR-novel-12026 | ENSOCUG00000010061 | PLEKHG4 | 0.0246 |
| ocu-cirR-novel-12026 | ENSOCUG00000010551 | SLC7A3 | 0.0370 |
| ocu-cirR-novel-12026 | ENSOCUG00000012034 | RBM47 | 0.0389 |
| ocu-cirR-novel-12026 | ENSOCUG00000012328 | RNF128 | 0.0370 |
| ocu-cirR-novel-12026 | ENSOCUG00000012400 | PTH1R | 0.0370 |
| ocu-cirR-novel-12026 | ENSOCUG00000016831 | SLC27A2 | 0.0370 |
| ocu-cirR-novel-12026 | ENSOCUG00000016963 | GNPDA1 | 0.0066 |
| ocu-cirR-novel-12026 | ENSOCUG00000021497 | ARHGAP19 | 0.0043 |
| ocu-cirR-novel-12026 | ENSOCUG00000025830 | CAPN5 | 0.0471 |
| ocu-cirR-novel-12331 | ENSOCUG00000000109 | ADAMTS9 | 0.0494 |
| ocu-cirR-novel-12331 | ENSOCUG00000002555 | SCP2 | 0.0052 |
| ocu-cirR-novel-12331 | ENSOCUG00000002623 | ESAM | 0.0071 |
| ocu-cirR-novel-12331 | ENSOCUG00000002624 | NRK | 0.0357 |
| ocu-cirR-novel-12331 | ENSOCUG00000003031 | GGH | 0.0176 |
| ocu-cirR-novel-12331 | ENSOCUG00000004035 | IRF6 | 0.0052 |
| ocu-cirR-novel-12331 | ENSOCUG00000005603 | TESPA1 | 0.0467 |
| ocu-cirR-novel-12331 | ENSOCUG00000005655 | SMC2 | 0.0467 |
| ocu-cirR-novel-12331 | ENSOCUG00000008204 | SOAT1 | 0.0105 |
| ocu-cirR-novel-12331 | ENSOCUG00000008346 | LYPD6B | 0.0055 |
| ocu-cirR-novel-12331 | ENSOCUG00000008474 | CD2 | 0.0259 |
| ocu-cirR-novel-12331 | ENSOCUG00000009077 | MS4A2 | 0.0019 |
| ocu-cirR-novel-12331 | ENSOCUG00000009311 | CD84 | 0.0467 |
| ocu-cirR-novel-12331 | ENSOCUG00000009882 | NCAPH | 0.0055 |
| ocu-cirR-novel-12331 | ENSOCUG00000011064 | CLSTN1 | 0.0276 |
| ocu-cirR-novel-12331 | ENSOCUG00000011298 | FAM83D | 0.0494 |
| ocu-cirR-novel-12331 | ENSOCUG00000012432 | HS3ST3B1 | 0.0121 |
| ocu-cirR-novel-12331 | ENSOCUG00000012455 | MDFIC | 0.0357 |
| ocu-cirR-novel-12331 | ENSOCUG00000013498 | ADAMTS4 | 0.0018 |
| ocu-cirR-novel-12331 | ENSOCUG00000014070 | ZNF697 | 0.0153 |
| ocu-cirR-novel-12331 | ENSOCUG00000014252 | MAPKAPK2 | 0.0121 |
| ocu-cirR-novel-12331 | ENSOCUG00000015020 | COL5A2 | 0.0357 |
| ocu-cirR-novel-12331 | ENSOCUG00000016959 | CRYBG2 | 0.0071 |
| ocu-cirR-novel-12331 | ENSOCUG00000017019 | CDH11 | 0.0107 |
| ocu-cirR-novel-12331 | ENSOCUG00000017438 | SELE | 0.0176 |
| ocu-cirR-novel-12331 | ENSOCUG00000017563 | AKAP1 | 0.0259 |
| ocu-cirR-novel-12331 | ENSOCUG00000021747 | WDR78 | 0.0494 |
| ocu-cirR-novel-12331 | ENSOCUG00000023764 | CCDC80 | 0.0107 |
| ocu-cirR-novel-12487 | ENSOCUG00000000575 | HAVCR1 | 0.0491 |
| ocu-cirR-novel-12487 | ENSOCUG00000001853 | SLC15A2 | 0.0204 |
| ocu-cirR-novel-12487 | ENSOCUG00000002254 | DNMBP | 0.0204 |
| ocu-cirR-novel-12487 | ENSOCUG00000003209 | CCBE1 | 0.0324 |
| ocu-cirR-novel-12487 | ENSOCUG00000003294 | WDR26 | 0.0204 |
| ocu-cirR-novel-12487 | ENSOCUG00000003863 | CD74 | 0.0491 |
| ocu-cirR-novel-12487 | ENSOCUG00000004035 | IRF6 | 0.0139 |
| ocu-cirR-novel-12487 | ENSOCUG00000004136 | SEPT-7 | 0.0111 |
| ocu-cirR-novel-12487 | ENSOCUG00000004293 | EPS15 | 0.0491 |
| ocu-cirR-novel-12487 | ENSOCUG00000006357 | ADAMTS12 | 0.0204 |
| ocu-cirR-novel-12487 | ENSOCUG00000008137 | MLLT3 | 0.0491 |
| ocu-cirR-novel-12487 | ENSOCUG00000009055 | CD69 | 0.0491 |
| ocu-cirR-novel-12487 | ENSOCUG00000009261 | TMEM40 | 0.0241 |
| ocu-cirR-novel-12487 | ENSOCUG00000009504 | CD80 | 0.0491 |
| ocu-cirR-novel-12487 | ENSOCUG00000009761 | TMEM71 | 0.0491 |
| ocu-cirR-novel-12487 | ENSOCUG00000010095 | ZNF18 | 0.0324 |
| ocu-cirR-novel-12487 | ENSOCUG00000010887 | RPS6KA1 | 0.0204 |
| ocu-cirR-novel-12487 | ENSOCUG00000011550 | CD86 | 0.0019 |
| ocu-cirR-novel-12487 | ENSOCUG00000013111 | PCOLCE2 | 0.0370 |
| ocu-cirR-novel-12487 | ENSOCUG00000013564 | TRIM63 | 0.0491 |
| ocu-cirR-novel-12487 | ENSOCUG00000015138 | C1QC | 0.0019 |
| ocu-cirR-novel-12487 | ENSOCUG00000015492 | GLP2R | 0.0065 |
| ocu-cirR-novel-12487 | ENSOCUG00000015651 | ATG3 | 0.0204 |
| ocu-cirR-novel-12487 | ENSOCUG00000016268 | RCN1 | 0.0420 |
| ocu-cirR-novel-12487 | ENSOCUG00000024225 | GMPPA | 0.0491 |
| ocu-cirR-novel-12487 | ENSOCUG00000025121 | ALDH6A1 | 0.0204 |
| ocu-cirR-novel-12487 | ENSOCUG00000026567 | RLA-DMB | 0.0491 |
| ocu-cirR-novel-12487 | ENSOCUG00000029154 | . | 0.0420 |
| ocu-cirR-novel-12487 | ENSOCUG00000029380 | ZNF311 | 0.0031 |
| ocu-cirR-novel-12487 | ENSOCUG00000029541 | ZNF157 | 0.0247 |
| ocu-cirR-novel-12538 | ENSOCUG00000001006 | TACR1 | 0.0314 |
| ocu-cirR-novel-12538 | ENSOCUG00000002858 | CDK1 | 0.0186 |
| ocu-cirR-novel-12538 | ENSOCUG00000003319 | HLA-DPA1 | 0.0471 |
| ocu-cirR-novel-12538 | ENSOCUG00000005655 | SMC2 | 0.0246 |
| ocu-cirR-novel-12538 | ENSOCUG00000006986 | SCIN | 0.0186 |
| ocu-cirR-novel-12538 | ENSOCUG00000008071 | . | 0.0370 |
| ocu-cirR-novel-12538 | ENSOCUG00000009863 | PRKCQ | 0.0019 |
| ocu-cirR-novel-12538 | ENSOCUG00000012034 | RBM47 | 0.0389 |
| ocu-cirR-novel-12538 | ENSOCUG00000012363 | AGTR2 | 0.0370 |
| ocu-cirR-novel-12538 | ENSOCUG00000012561 | SLC43A3 | 0.0055 |
| ocu-cirR-novel-12538 | ENSOCUG00000012754 | RUBCN | 0.0134 |
| ocu-cirR-novel-12538 | ENSOCUG00000014988 | COL3A1 | 0.0370 |
| ocu-cirR-novel-12538 | ENSOCUG00000021980 | DNAJC14 | 0.0471 |
| ocu-cirR-novel-12538 | ENSOCUG00000023553 | GFI1 | 0.0370 |
| ocu-cirR-novel-12538 | ENSOCUG00000025512 | SPINK8 | 0.0370 |
| ocu-cirR-novel-12538 | ENSOCUG00000026966 | SASH3 | 0.0186 |
| ocu-cirR-novel-12538 | ENSOCUG00000029569 | KRT17 | 0.0370 |
| ocu-cirR-novel-12686 | ENSOCUG00000002624 | NRK | 0.0357 |
| ocu-cirR-novel-12686 | ENSOCUG00000003031 | GGH | 0.0176 |
| ocu-cirR-novel-12686 | ENSOCUG00000003299 | DMRT2 | 0.0176 |
| ocu-cirR-novel-12686 | ENSOCUG00000004451 | LIMS1 | 0.0094 |
| ocu-cirR-novel-12686 | ENSOCUG00000005603 | TESPA1 | 0.0467 |
| ocu-cirR-novel-12686 | ENSOCUG00000005655 | SMC2 | 0.0467 |
| ocu-cirR-novel-12686 | ENSOCUG00000008346 | LYPD6B | 0.0055 |
| ocu-cirR-novel-12686 | ENSOCUG00000008474 | CD2 | 0.0259 |
| ocu-cirR-novel-12686 | ENSOCUG00000009077 | MS4A2 | 0.0019 |
| ocu-cirR-novel-12686 | ENSOCUG00000009311 | CD84 | 0.0467 |
| ocu-cirR-novel-12686 | ENSOCUG00000009882 | NCAPH | 0.0055 |
| ocu-cirR-novel-12686 | ENSOCUG00000010887 | RPS6KA1 | 0.0094 |
| ocu-cirR-novel-12686 | ENSOCUG00000011064 | CLSTN1 | 0.0276 |
| ocu-cirR-novel-12686 | ENSOCUG00000012455 | MDFIC | 0.0357 |
| ocu-cirR-novel-12686 | ENSOCUG00000012457 | GOLGB1 | 0.0094 |
| ocu-cirR-novel-12686 | ENSOCUG00000013078 | FAXDC2 | 0.0327 |
| ocu-cirR-novel-12686 | ENSOCUG00000014070 | ZNF697 | 0.0153 |
| ocu-cirR-novel-12686 | ENSOCUG00000014252 | MAPKAPK2 | 0.0121 |
| ocu-cirR-novel-12686 | ENSOCUG00000015020 | COL5A2 | 0.0357 |
| ocu-cirR-novel-12686 | ENSOCUG00000017023 | ATF1 | 0.0037 |
| ocu-cirR-novel-12686 | ENSOCUG00000017438 | SELE | 0.0176 |
| ocu-cirR-novel-12686 | ENSOCUG00000017563 | AKAP1 | 0.0259 |
| ocu-cirR-novel-12686 | ENSOCUG00000023764 | CCDC80 | 0.0107 |
| ocu-cirR-novel-12689 | ENSOCUG00000002624 | NRK | 0.0357 |
| ocu-cirR-novel-12689 | ENSOCUG00000003031 | GGH | 0.0176 |
| ocu-cirR-novel-12689 | ENSOCUG00000003299 | DMRT2 | 0.0176 |
| ocu-cirR-novel-12689 | ENSOCUG00000004451 | LIMS1 | 0.0094 |
| ocu-cirR-novel-12689 | ENSOCUG00000005603 | TESPA1 | 0.0467 |
| ocu-cirR-novel-12689 | ENSOCUG00000005655 | SMC2 | 0.0467 |
| ocu-cirR-novel-12689 | ENSOCUG00000008346 | LYPD6B | 0.0055 |
| ocu-cirR-novel-12689 | ENSOCUG00000008474 | CD2 | 0.0259 |
| ocu-cirR-novel-12689 | ENSOCUG00000009077 | MS4A2 | 0.0019 |
| ocu-cirR-novel-12689 | ENSOCUG00000009311 | CD84 | 0.0467 |
| ocu-cirR-novel-12689 | ENSOCUG00000009882 | NCAPH | 0.0055 |
| ocu-cirR-novel-12689 | ENSOCUG00000010887 | RPS6KA1 | 0.0094 |
| ocu-cirR-novel-12689 | ENSOCUG00000011064 | CLSTN1 | 0.0276 |
| ocu-cirR-novel-12689 | ENSOCUG00000012455 | MDFIC | 0.0357 |
| ocu-cirR-novel-12689 | ENSOCUG00000012457 | GOLGB1 | 0.0094 |
| ocu-cirR-novel-12689 | ENSOCUG00000013078 | FAXDC2 | 0.0327 |
| ocu-cirR-novel-12689 | ENSOCUG00000014070 | ZNF697 | 0.0153 |
| ocu-cirR-novel-12689 | ENSOCUG00000014252 | MAPKAPK2 | 0.0121 |
| ocu-cirR-novel-12689 | ENSOCUG00000015020 | COL5A2 | 0.0357 |
| ocu-cirR-novel-12689 | ENSOCUG00000017023 | ATF1 | 0.0037 |
| ocu-cirR-novel-12689 | ENSOCUG00000017438 | SELE | 0.0176 |
| ocu-cirR-novel-12689 | ENSOCUG00000017563 | AKAP1 | 0.0259 |
| ocu-cirR-novel-12689 | ENSOCUG00000023764 | CCDC80 | 0.0107 |
| ocu-cirR-novel-12697 | ENSOCUG00000003999 | CAMK1G | 0.0134 |
| ocu-cirR-novel-12697 | ENSOCUG00000012363 | AGTR2 | 0.0370 |
| ocu-cirR-novel-12697 | ENSOCUG00000013567 | GRN | 0.0246 |
| ocu-cirR-novel-12703 | ENSOCUG00000000930 | KIF15 | 0.0417 |
| ocu-cirR-novel-12703 | ENSOCUG00000001553 | LIPA | 0.0259 |
| ocu-cirR-novel-12703 | ENSOCUG00000001751 | RND3 | 0.0228 |
| ocu-cirR-novel-12703 | ENSOCUG00000001819 | ANAPC10 | 0.0046 |
| ocu-cirR-novel-12703 | ENSOCUG00000004617 | ANKRD22 | 0.0417 |
| ocu-cirR-novel-12703 | ENSOCUG00000005294 | RRAGD | 0.0022 |
| ocu-cirR-novel-12703 | ENSOCUG00000006194 | KYNU | 0.0393 |
| ocu-cirR-novel-12703 | ENSOCUG00000006632 | MREG | 0.0165 |
| ocu-cirR-novel-12703 | ENSOCUG00000006719 | LPCAT2 | 0.0304 |
| ocu-cirR-novel-12703 | ENSOCUG00000008226 | FBN2 | 0.0417 |
| ocu-cirR-novel-12703 | ENSOCUG00000008568 | ANKRD28 | 0.0495 |
| ocu-cirR-novel-12703 | ENSOCUG00000009195 | CLEC4E | 0.0259 |
| ocu-cirR-novel-12703 | ENSOCUG00000012457 | GOLGB1 | 0.0393 |
| ocu-cirR-novel-12703 | ENSOCUG00000013696 | BICC1 | 0.0053 |
| ocu-cirR-novel-12703 | ENSOCUG00000014332 | SHF | 0.0259 |
| ocu-cirR-novel-12703 | ENSOCUG00000014811 | KIAA1109 | 0.0417 |
| ocu-cirR-novel-12703 | ENSOCUG00000015410 | NDC80 | 0.0017 |
| ocu-cirR-novel-12703 | ENSOCUG00000015619 | CALB2 | 0.0259 |
| ocu-cirR-novel-12703 | ENSOCUG00000021706 | TRAV9-2 | 0.0046 |
| ocu-cirR-novel-12703 | ENSOCUG00000024777 | . | 0.0417 |
| ocu-cirR-novel-12703 | ENSOCUG00000026691 | SLC31A2 | 0.0026 |
| ocu-cirR-novel-12789 | ENSOCUG00000001165 | RANBP2 | 0.0247 |
| ocu-cirR-novel-12789 | ENSOCUG00000003850 | PTCRA | 0.0370 |
| ocu-cirR-novel-12789 | ENSOCUG00000004244 | KLF12 | 0.0370 |
| ocu-cirR-novel-12789 | ENSOCUG00000024081 | CD63 | 0.0247 |
| ocu-cirR-novel-12789 | ENSOCUG00000024139 | TRA2A | 0.0123 |
| ocu-cirR-novel-13035 | ENSOCUG00000000109 | ADAMTS9 | 0.0370 |
| ocu-cirR-novel-13035 | ENSOCUG00000001006 | TACR1 | 0.0314 |
| ocu-cirR-novel-13035 | ENSOCUG00000001751 | RND3 | 0.0389 |
| ocu-cirR-novel-13035 | ENSOCUG00000002835 | CTGF | 0.0370 |
| ocu-cirR-novel-13035 | ENSOCUG00000003319 | HLA-DPA1 | 0.0471 |
| ocu-cirR-novel-13035 | ENSOCUG00000012578 | CAMK2G | 0.0246 |
| ocu-cirR-novel-13035 | ENSOCUG00000016883 | FERMT1 | 0.0389 |
| ocu-cirR-novel-13035 | ENSOCUG00000021747 | WDR78 | 0.0370 |
| ocu-cirR-novel-13035 | ENSOCUG00000023896 | E2F2 | 0.0471 |
| ocu-cirR-novel-13059 | ENSOCUG00000000575 | HAVCR1 | 0.0491 |
| ocu-cirR-novel-13059 | ENSOCUG00000002555 | SCP2 | 0.0139 |
| ocu-cirR-novel-13059 | ENSOCUG00000003209 | CCBE1 | 0.0324 |
| ocu-cirR-novel-13059 | ENSOCUG00000004035 | IRF6 | 0.0139 |
| ocu-cirR-novel-13059 | ENSOCUG00000005603 | TESPA1 | 0.0086 |
| ocu-cirR-novel-13059 | ENSOCUG00000009077 | MS4A2 | 0.0491 |
| ocu-cirR-novel-13059 | ENSOCUG00000009761 | TMEM71 | 0.0491 |
| ocu-cirR-novel-13059 | ENSOCUG00000010095 | ZNF18 | 0.0324 |
| ocu-cirR-novel-13059 | ENSOCUG00000010887 | RPS6KA1 | 0.0204 |
| ocu-cirR-novel-13059 | ENSOCUG00000013498 | ADAMTS4 | 0.0472 |
| ocu-cirR-novel-13059 | ENSOCUG00000015020 | COL5A2 | 0.0065 |
| ocu-cirR-novel-13059 | ENSOCUG00000016254 | STAB2 | 0.0204 |
| ocu-cirR-novel-13059 | ENSOCUG00000016268 | RCN1 | 0.0420 |
| ocu-cirR-novel-13059 | ENSOCUG00000017438 | SELE | 0.0031 |
| ocu-cirR-novel-13059 | ENSOCUG00000024225 | GMPPA | 0.0491 |
| ocu-cirR-novel-13059 | ENSOCUG00000029154 | . | 0.0420 |
| ocu-cirR-novel-13061 | ENSOCUG00000000787 | PTPRO | 0.0170 |
| ocu-cirR-novel-13061 | ENSOCUG00000009863 | PRKCQ | 0.0170 |
| ocu-cirR-novel-13061 | ENSOCUG00000013696 | BICC1 | 0.0329 |
| ocu-cirR-novel-13061 | ENSOCUG00000016706 | KIF11 | 0.0314 |
| ocu-cirR-novel-13065 | ENSOCUG00000000787 | PTPRO | 0.0165 |
| ocu-cirR-novel-13065 | ENSOCUG00000015897 | ASF1A | 0.0286 |
| ocu-cirR-novel-13065 | ENSOCUG00000016432 | OLFM4 | 0.0417 |
| ocu-cirR-novel-13067 | ENSOCUG00000000787 | PTPRO | 0.0471 |
| ocu-cirR-novel-13067 | ENSOCUG00000001542 | RIPK2 | 0.0370 |
| ocu-cirR-novel-13067 | ENSOCUG00000007702 | TBC1D10C | 0.0389 |
| ocu-cirR-novel-13067 | ENSOCUG00000008983 | CTCF | 0.0471 |
| ocu-cirR-novel-13067 | ENSOCUG00000015897 | ASF1A | 0.0090 |
| ocu-cirR-novel-13067 | ENSOCUG00000016432 | OLFM4 | 0.0134 |
| ocu-cirR-novel-13067 | ENSOCUG00000016883 | FERMT1 | 0.0389 |
| ocu-cirR-novel-13067 | ENSOCUG00000029399 | SH2D1B | 0.0471 |
| ocu-cirR-novel-13094 | ENSOCUG00000009504 | CD80 | 0.0491 |
| ocu-cirR-novel-13094 | ENSOCUG00000012363 | AGTR2 | 0.0247 |
| ocu-cirR-novel-13094 | ENSOCUG00000014003 | BUB1 | 0.0491 |
| ocu-cirR-novel-13356 | ENSOCUG00000001504 | RPA1 | 0.0370 |
| ocu-cirR-novel-13356 | ENSOCUG00000001553 | LIPA | 0.0494 |
| ocu-cirR-novel-13356 | ENSOCUG00000003089 | PRG3 | 0.0123 |
| ocu-cirR-novel-13356 | ENSOCUG00000003276 | PAX1 | 0.0247 |
| ocu-cirR-novel-13356 | ENSOCUG00000003863 | CD74 | 0.0247 |
| ocu-cirR-novel-13356 | ENSOCUG00000006320 | SMARCA2 | 0.0247 |
| ocu-cirR-novel-13356 | ENSOCUG00000006797 | MED12 | 0.0494 |
| ocu-cirR-novel-13356 | ENSOCUG00000006970 | SLC44A4 | 0.0123 |
| ocu-cirR-novel-13356 | ENSOCUG00000009047 | FCRL2 | 0.0247 |
| ocu-cirR-novel-13356 | ENSOCUG00000010247 | ATP6V0D2 | 0.0247 |
| ocu-cirR-novel-13356 | ENSOCUG00000010633 | HMOX1 | 0.0247 |
| ocu-cirR-novel-13356 | ENSOCUG00000010649 | MKX | 0.0494 |
| ocu-cirR-novel-13356 | ENSOCUG00000011270 | CD1B | 0.0370 |
| ocu-cirR-novel-13356 | ENSOCUG00000015493 | ANXA1 | 0.0370 |
| ocu-cirR-novel-13356 | ENSOCUG00000015599 | GEMIN6 | 0.0247 |
| ocu-cirR-novel-13356 | ENSOCUG00000016465 | TP63 | 0.0494 |
| ocu-cirR-novel-13356 | ENSOCUG00000016500 | . | 0.0247 |
| ocu-cirR-novel-13356 | ENSOCUG00000025091 | AARD | 0.0247 |
| ocu-cirR-novel-13356 | ENSOCUG00000025569 | CCR7 | 0.0494 |
| ocu-cirR-novel-13356 | ENSOCUG00000029127 | CD164 | 0.0370 |
| ocu-cirR-novel-13500 | ENSOCUG00000001316 | ESRP1 | 0.0370 |
| ocu-cirR-novel-13500 | ENSOCUG00000001414 | DDX23 | 0.0494 |
| ocu-cirR-novel-13500 | ENSOCUG00000003165 | SCUBE2 | 0.0370 |
| ocu-cirR-novel-13500 | ENSOCUG00000005012 | SH2D1A | 0.0370 |
| ocu-cirR-novel-13500 | ENSOCUG00000005094 | INHBB | 0.0370 |
| ocu-cirR-novel-13500 | ENSOCUG00000005729 | RIN3 | 0.0494 |
| ocu-cirR-novel-13500 | ENSOCUG00000005937 | SCD5 | 0.0247 |
| ocu-cirR-novel-13500 | ENSOCUG00000009195 | CLEC4E | 0.0494 |
| ocu-cirR-novel-13500 | ENSOCUG00000009701 | CSF3 | 0.0494 |
| ocu-cirR-novel-13500 | ENSOCUG00000009932 | ESCO2 | 0.0370 |
| ocu-cirR-novel-13500 | ENSOCUG00000010203 | TLN1 | 0.0370 |
| ocu-cirR-novel-13500 | ENSOCUG00000010247 | ATP6V0D2 | 0.0247 |
| ocu-cirR-novel-13500 | ENSOCUG00000014988 | COL3A1 | 0.0123 |
| ocu-cirR-novel-13500 | ENSOCUG00000015138 | C1QC | 0.0494 |
| ocu-cirR-novel-13500 | ENSOCUG00000025512 | SPINK8 | 0.0123 |
| ocu-cirR-novel-13500 | ENSOCUG00000025590 | LAT | 0.0247 |
| ocu-cirR-novel-13500 | ENSOCUG00000026887 | . | 0.0494 |
| ocu-cirR-novel-13500 | ENSOCUG00000027275 | TM4SF1 | 0.0494 |
| ocu-cirR-novel-13500 | ENSOCUG00000029569 | KRT17 | 0.0123 |
| ocu-cirR-novel-13719 | ENSOCUG00000025803 | LGALS9 | 0.0247 |
| ocu-cirR-novel-13719 | ENSOCUG00000026988 | CHI3L1 | 0.0170 |
| ocu-cirR-novel-13897 | ENSOCUG00000009965 | TYR | 0.0370 |
| ocu-cirR-novel-13897 | ENSOCUG00000013505 | CPSF6 | 0.0246 |
| ocu-cirR-novel-13897 | ENSOCUG00000013593 | BPI | 0.0370 |
| ocu-cirR-novel-13897 | ENSOCUG00000015431 | DOCK10 | 0.0370 |
| ocu-cirR-novel-13897 | ENSOCUG00000024358 | CDIPT | 0.0471 |
| ocu-cirR-novel-14100 | ENSOCUG00000003294 | WDR26 | 0.0094 |
| ocu-cirR-novel-14100 | ENSOCUG00000004632 | CA12 | 0.0276 |
| ocu-cirR-novel-14100 | ENSOCUG00000006752 | MEST | 0.0023 |
| ocu-cirR-novel-14100 | ENSOCUG00000008562 | BTD | 0.0494 |
| ocu-cirR-novel-14100 | ENSOCUG00000010104 | FN1 | 0.0494 |
| ocu-cirR-novel-14100 | ENSOCUG00000013454 | PRDM5 | 0.0259 |
| ocu-cirR-novel-14100 | ENSOCUG00000025803 | LGALS9 | 0.0494 |
| ocu-cirR-novel-14173 | ENSOCUG00000007483 | TBC1D12 | 0.0491 |
| ocu-cirR-novel-14330 | ENSOCUG00000000109 | ADAMTS9 | 0.0370 |
| ocu-cirR-novel-14330 | ENSOCUG00000021747 | WDR78 | 0.0370 |
| ocu-cirR-novel-14389 | ENSOCUG00000000540 | KCNIP1 | 0.0055 |
| ocu-cirR-novel-14389 | ENSOCUG00000000787 | PTPRO | 0.0471 |
| ocu-cirR-novel-14389 | ENSOCUG00000001006 | TACR1 | 0.0314 |
| ocu-cirR-novel-14389 | ENSOCUG00000001964 | LARS2 | 0.0237 |
| ocu-cirR-novel-14389 | ENSOCUG00000002260 | ACSS2 | 0.0055 |
| ocu-cirR-novel-14389 | ENSOCUG00000002623 | ESAM | 0.0471 |
| ocu-cirR-novel-14389 | ENSOCUG00000006045 | EZH1 | 0.0156 |
| ocu-cirR-novel-14389 | ENSOCUG00000006186 | CPXM1 | 0.0246 |
| ocu-cirR-novel-14389 | ENSOCUG00000006719 | LPCAT2 | 0.0471 |
| ocu-cirR-novel-14389 | ENSOCUG00000009260 | PLCH1 | 0.0026 |
| ocu-cirR-novel-14389 | ENSOCUG00000009929 | C3orf52 | 0.0389 |
| ocu-cirR-novel-14389 | ENSOCUG00000009965 | TYR | 0.0370 |
| ocu-cirR-novel-14389 | ENSOCUG00000010671 | SH2D2A | 0.0090 |
| ocu-cirR-novel-14389 | ENSOCUG00000011064 | CLSTN1 | 0.0080 |
| ocu-cirR-novel-14389 | ENSOCUG00000011080 | GALNT7 | 0.0186 |
| ocu-cirR-novel-14389 | ENSOCUG00000013078 | FAXDC2 | 0.0096 |
| ocu-cirR-novel-14389 | ENSOCUG00000013505 | CPSF6 | 0.0246 |
| ocu-cirR-novel-14389 | ENSOCUG00000013961 | DNAH11 | 0.0370 |
| ocu-cirR-novel-14389 | ENSOCUG00000015619 | CALB2 | 0.0055 |
| ocu-cirR-novel-14389 | ENSOCUG00000016073 | OSTM1 | 0.0186 |
| ocu-cirR-novel-14389 | ENSOCUG00000016477 | SELP | 0.0314 |
| ocu-cirR-novel-14389 | ENSOCUG00000016883 | FERMT1 | 0.0389 |
| ocu-cirR-novel-14389 | ENSOCUG00000017308 | FLOT1 | 0.0055 |
| ocu-cirR-novel-14389 | ENSOCUG00000017747 | CPNE8 | 0.0090 |
| ocu-cirR-novel-14389 | ENSOCUG00000023078 | . | 0.0053 |
| ocu-cirR-novel-14389 | ENSOCUG00000024358 | CDIPT | 0.0471 |
| ocu-cirR-novel-14389 | ENSOCUG00000025012 | ANGPT4 | 0.0134 |
| ocu-cirR-novel-14389 | ENSOCUG00000025830 | CAPN5 | 0.0471 |
| ocu-cirR-novel-14389 | ENSOCUG00000029154 | . | 0.0080 |
| ocu-cirR-novel-14389 | ENSOCUG00000029380 | ZNF311 | 0.0090 |
| ocu-cirR-novel-14389 | ENSOCUG00000029742 | MAP1LC3C | 0.0389 |
| ocu-cirR-novel-14397 | ENSOCUG00000008346 | LYPD6B | 0.0370 |
| ocu-cirR-novel-14397 | ENSOCUG00000009077 | MS4A2 | 0.0247 |
| ocu-cirR-novel-14397 | ENSOCUG00000009701 | CSF3 | 0.0494 |
| ocu-cirR-novel-14397 | ENSOCUG00000009882 | NCAPH | 0.0370 |
| ocu-cirR-novel-14397 | ENSOCUG00000023764 | CCDC80 | 0.0494 |
| ocu-cirR-novel-14731 | ENSOCUG00000000540 | KCNIP1 | 0.0494 |
| ocu-cirR-novel-14731 | ENSOCUG00000007483 | TBC1D12 | 0.0247 |
| ocu-cirR-novel-14731 | ENSOCUG00000025540 | NFYC | 0.0494 |
| ocu-cirR-novel-14939 | ENSOCUG00000000575 | HAVCR1 | 0.0247 |
| ocu-cirR-novel-14939 | ENSOCUG00000001316 | ESRP1 | 0.0370 |
| ocu-cirR-novel-14939 | ENSOCUG00000001504 | RPA1 | 0.0370 |
| ocu-cirR-novel-14939 | ENSOCUG00000009761 | TMEM71 | 0.0247 |
| ocu-cirR-novel-14939 | ENSOCUG00000011550 | CD86 | 0.0494 |
| ocu-cirR-novel-14939 | ENSOCUG00000013625 | PRSS12 | 0.0370 |
| ocu-cirR-novel-14939 | ENSOCUG00000015138 | C1QC | 0.0494 |
| ocu-cirR-novel-14939 | ENSOCUG00000024225 | GMPPA | 0.0247 |
| ocu-cirR-novel-14939 | ENSOCUG00000026887 | . | 0.0494 |
| ocu-cirR-novel-15158 | ENSOCUG00000000574 | PLEKHH1 | 0.0286 |
| ocu-cirR-novel-15158 | ENSOCUG00000001964 | LARS2 | 0.0253 |
| ocu-cirR-novel-15158 | ENSOCUG00000003299 | DMRT2 | 0.0286 |
| ocu-cirR-novel-15158 | ENSOCUG00000008620 | DAPL1 | 0.0286 |
| ocu-cirR-novel-15158 | ENSOCUG00000015166 | . | 0.0417 |
| ocu-cirR-novel-15158 | ENSOCUG00000015723 | SLC15A3 | 0.0286 |
| ocu-cirR-novel-15353 | ENSOCUG00000000575 | HAVCR1 | 0.0491 |
| ocu-cirR-novel-15353 | ENSOCUG00000002555 | SCP2 | 0.0139 |
| ocu-cirR-novel-15353 | ENSOCUG00000003209 | CCBE1 | 0.0324 |
| ocu-cirR-novel-15353 | ENSOCUG00000005603 | TESPA1 | 0.0086 |
| ocu-cirR-novel-15353 | ENSOCUG00000005883 | GPR87 | 0.0204 |
| ocu-cirR-novel-15353 | ENSOCUG00000009077 | MS4A2 | 0.0491 |
| ocu-cirR-novel-15353 | ENSOCUG00000009761 | TMEM71 | 0.0491 |
| ocu-cirR-novel-15353 | ENSOCUG00000010887 | RPS6KA1 | 0.0204 |
| ocu-cirR-novel-15353 | ENSOCUG00000013498 | ADAMTS4 | 0.0472 |
| ocu-cirR-novel-15353 | ENSOCUG00000015020 | COL5A2 | 0.0065 |
| ocu-cirR-novel-15353 | ENSOCUG00000016254 | STAB2 | 0.0204 |
| ocu-cirR-novel-15353 | ENSOCUG00000016268 | RCN1 | 0.0420 |
| ocu-cirR-novel-15353 | ENSOCUG00000017438 | SELE | 0.0031 |
| ocu-cirR-novel-15353 | ENSOCUG00000023896 | E2F2 | 0.0170 |
| ocu-cirR-novel-15353 | ENSOCUG00000024225 | GMPPA | 0.0491 |
| ocu-cirR-novel-15353 | ENSOCUG00000029154 | . | 0.0420 |
| ocu-cirR-novel-15353 | ENSOCUG00000029216 | C17H15orf48 | 0.0139 |
| ocu-cirR-novel-15395 | ENSOCUG00000002858 | CDK1 | 0.0186 |
| ocu-cirR-novel-15395 | ENSOCUG00000003313 | LOX | 0.0314 |
| ocu-cirR-novel-15395 | ENSOCUG00000003999 | CAMK1G | 0.0134 |
| ocu-cirR-novel-15395 | ENSOCUG00000008071 | . | 0.0370 |
| ocu-cirR-novel-15395 | ENSOCUG00000009863 | PRKCQ | 0.0471 |
| ocu-cirR-novel-15395 | ENSOCUG00000010104 | FN1 | 0.0370 |
| ocu-cirR-novel-15395 | ENSOCUG00000010436 | LEF1 | 0.0134 |
| ocu-cirR-novel-15395 | ENSOCUG00000012754 | RUBCN | 0.0134 |
| ocu-cirR-novel-15395 | ENSOCUG00000012911 | CD4 | 0.0186 |
| ocu-cirR-novel-15395 | ENSOCUG00000013454 | PRDM5 | 0.0134 |
| ocu-cirR-novel-15395 | ENSOCUG00000021980 | DNAJC14 | 0.0471 |
| ocu-cirR-novel-15395 | ENSOCUG00000023553 | GFI1 | 0.0370 |
| ocu-cirR-novel-15395 | ENSOCUG00000026480 | AOX2 | 0.0186 |
| ocu-cirR-novel-15409 | ENSOCUG00000002555 | SCP2 | 0.0052 |
| ocu-cirR-novel-15409 | ENSOCUG00000002624 | NRK | 0.0357 |
| ocu-cirR-novel-15409 | ENSOCUG00000002858 | CDK1 | 0.0357 |
| ocu-cirR-novel-15409 | ENSOCUG00000003031 | GGH | 0.0176 |
| ocu-cirR-novel-15409 | ENSOCUG00000005603 | TESPA1 | 0.0467 |
| ocu-cirR-novel-15409 | ENSOCUG00000005655 | SMC2 | 0.0025 |
| ocu-cirR-novel-15409 | ENSOCUG00000008071 | . | 0.0494 |
| ocu-cirR-novel-15409 | ENSOCUG00000008346 | LYPD6B | 0.0055 |
| ocu-cirR-novel-15409 | ENSOCUG00000008474 | CD2 | 0.0259 |
| ocu-cirR-novel-15409 | ENSOCUG00000009077 | MS4A2 | 0.0019 |
| ocu-cirR-novel-15409 | ENSOCUG00000009311 | CD84 | 0.0467 |
| ocu-cirR-novel-15409 | ENSOCUG00000009882 | NCAPH | 0.0055 |
| ocu-cirR-novel-15409 | ENSOCUG00000012455 | MDFIC | 0.0357 |
| ocu-cirR-novel-15409 | ENSOCUG00000014070 | ZNF697 | 0.0153 |
| ocu-cirR-novel-15409 | ENSOCUG00000015020 | COL5A2 | 0.0357 |
| ocu-cirR-novel-15409 | ENSOCUG00000017438 | SELE | 0.0176 |
| ocu-cirR-novel-15409 | ENSOCUG00000017563 | AKAP1 | 0.0259 |
| ocu-cirR-novel-15409 | ENSOCUG00000023553 | GFI1 | 0.0494 |
| ocu-cirR-novel-15409 | ENSOCUG00000023764 | CCDC80 | 0.0107 |
| ocu-cirR-novel-15560 | ENSOCUG00000001142 | SNX13 | 0.0111 |
| ocu-cirR-novel-15560 | ENSOCUG00000001819 | ANAPC10 | 0.0111 |
| ocu-cirR-novel-15560 | ENSOCUG00000002623 | ESAM | 0.0017 |
| ocu-cirR-novel-15560 | ENSOCUG00000002624 | NRK | 0.0271 |
| ocu-cirR-novel-15560 | ENSOCUG00000003715 | LAX1 | 0.0241 |
| ocu-cirR-novel-15560 | ENSOCUG00000004337 | SMARCD2 | 0.0292 |
| ocu-cirR-novel-15560 | ENSOCUG00000005439 | CCNJL | 0.0435 |
| ocu-cirR-novel-15560 | ENSOCUG00000005883 | GPR87 | 0.0241 |
| ocu-cirR-novel-15560 | ENSOCUG00000006194 | KYNU | 0.0241 |
| ocu-cirR-novel-15560 | ENSOCUG00000006697 | HK1 | 0.0329 |
| ocu-cirR-novel-15560 | ENSOCUG00000006719 | LPCAT2 | 0.0170 |
| ocu-cirR-novel-15560 | ENSOCUG00000008346 | LYPD6B | 0.0314 |
| ocu-cirR-novel-15560 | ENSOCUG00000008474 | CD2 | 0.0165 |
| ocu-cirR-novel-15560 | ENSOCUG00000009077 | MS4A2 | 0.0111 |
| ocu-cirR-novel-15560 | ENSOCUG00000009260 | PLCH1 | 0.0241 |
| ocu-cirR-novel-15560 | ENSOCUG00000009882 | NCAPH | 0.0314 |
| ocu-cirR-novel-15560 | ENSOCUG00000010009 | EPX | 0.0226 |
| ocu-cirR-novel-15560 | ENSOCUG00000012457 | GOLGB1 | 0.0241 |
| ocu-cirR-novel-15560 | ENSOCUG00000012939 | ENPP5 | 0.0271 |
| ocu-cirR-novel-15560 | ENSOCUG00000013498 | ADAMTS4 | 0.0031 |
| ocu-cirR-novel-15560 | ENSOCUG00000013725 | FAM20A | 0.0021 |
| ocu-cirR-novel-15560 | ENSOCUG00000014070 | ZNF697 | 0.0435 |
| ocu-cirR-novel-15560 | ENSOCUG00000015619 | CALB2 | 0.0037 |
| ocu-cirR-novel-15560 | ENSOCUG00000016959 | CRYBG2 | 0.0170 |
| ocu-cirR-novel-15560 | ENSOCUG00000017563 | AKAP1 | 0.0165 |
| ocu-cirR-novel-15560 | ENSOCUG00000021506 | FOS | 0.0314 |
| ocu-cirR-novel-15560 | ENSOCUG00000021706 | TRAV9-2 | 0.0111 |
| ocu-cirR-novel-15560 | ENSOCUG00000022575 | VTN | 0.0408 |
| ocu-cirR-novel-15560 | ENSOCUG00000024789 | LPAR3 | 0.0065 |
| ocu-cirR-novel-15560 | ENSOCUG00000027626 | CD247 | 0.0073 |
| ocu-cirR-novel-15632 | ENSOCUG00000009828 | ARPP21 | 0.0370 |
| ocu-cirR-novel-15632 | ENSOCUG00000009932 | ESCO2 | 0.0370 |
| ocu-cirR-novel-15632 | ENSOCUG00000025569 | CCR7 | 0.0494 |
| ocu-cirR-novel-15840 | ENSOCUG00000001504 | RPA1 | 0.0370 |
| ocu-cirR-novel-15840 | ENSOCUG00000001553 | LIPA | 0.0494 |
| ocu-cirR-novel-15840 | ENSOCUG00000003089 | PRG3 | 0.0123 |
| ocu-cirR-novel-15840 | ENSOCUG00000003276 | PAX1 | 0.0247 |
| ocu-cirR-novel-15840 | ENSOCUG00000003863 | CD74 | 0.0247 |
| ocu-cirR-novel-15840 | ENSOCUG00000006320 | SMARCA2 | 0.0247 |
| ocu-cirR-novel-15840 | ENSOCUG00000006797 | MED12 | 0.0494 |
| ocu-cirR-novel-15840 | ENSOCUG00000006970 | SLC44A4 | 0.0123 |
| ocu-cirR-novel-15840 | ENSOCUG00000009047 | FCRL2 | 0.0247 |
| ocu-cirR-novel-15840 | ENSOCUG00000010247 | ATP6V0D2 | 0.0247 |
| ocu-cirR-novel-15840 | ENSOCUG00000010633 | HMOX1 | 0.0247 |
| ocu-cirR-novel-15840 | ENSOCUG00000010649 | MKX | 0.0494 |
| ocu-cirR-novel-15840 | ENSOCUG00000011270 | CD1B | 0.0370 |
| ocu-cirR-novel-15840 | ENSOCUG00000015493 | ANXA1 | 0.0370 |
| ocu-cirR-novel-15840 | ENSOCUG00000015599 | GEMIN6 | 0.0247 |
| ocu-cirR-novel-15840 | ENSOCUG00000016465 | TP63 | 0.0494 |
| ocu-cirR-novel-15840 | ENSOCUG00000016500 | . | 0.0247 |
| ocu-cirR-novel-15840 | ENSOCUG00000025091 | AARD | 0.0247 |
| ocu-cirR-novel-15840 | ENSOCUG00000025569 | CCR7 | 0.0494 |
| ocu-cirR-novel-15840 | ENSOCUG00000029127 | CD164 | 0.0370 |
| ocu-cirR-novel-15925 | ENSOCUG00000000540 | KCNIP1 | 0.0494 |
| ocu-cirR-novel-15925 | ENSOCUG00000000788 | GADL1 | 0.0494 |
| ocu-cirR-novel-15925 | ENSOCUG00000003863 | CD74 | 0.0247 |
| ocu-cirR-novel-15925 | ENSOCUG00000004293 | EPS15 | 0.0247 |
| ocu-cirR-novel-15925 | ENSOCUG00000006540 | PLCB2 | 0.0370 |
| ocu-cirR-novel-15925 | ENSOCUG00000007355 | DSG2 | 0.0494 |
| ocu-cirR-novel-15925 | ENSOCUG00000008137 | MLLT3 | 0.0247 |
| ocu-cirR-novel-15925 | ENSOCUG00000008142 | TCF7L2 | 0.0370 |
| ocu-cirR-novel-15925 | ENSOCUG00000009055 | CD69 | 0.0247 |
| ocu-cirR-novel-15925 | ENSOCUG00000009504 | CD80 | 0.0247 |
| ocu-cirR-novel-15925 | ENSOCUG00000009701 | CSF3 | 0.0494 |
| ocu-cirR-novel-15925 | ENSOCUG00000009828 | ARPP21 | 0.0370 |
| ocu-cirR-novel-15925 | ENSOCUG00000010203 | TLN1 | 0.0370 |
| ocu-cirR-novel-15925 | ENSOCUG00000011289 | ALCAM | 0.0370 |
| ocu-cirR-novel-15925 | ENSOCUG00000011550 | CD86 | 0.0494 |
| ocu-cirR-novel-15925 | ENSOCUG00000013564 | TRIM63 | 0.0247 |
| ocu-cirR-novel-15925 | ENSOCUG00000014332 | SHF | 0.0494 |
| ocu-cirR-novel-15925 | ENSOCUG00000015138 | C1QC | 0.0494 |
| ocu-cirR-novel-15925 | ENSOCUG00000022391 | . | 0.0494 |
| ocu-cirR-novel-15925 | ENSOCUG00000026567 | RLA-DMB | 0.0247 |
| ocu-cirR-novel-15925 | ENSOCUG00000027275 | TM4SF1 | 0.0494 |
| ocu-cirR-novel-15925 | ENSOCUG00000029541 | ZNF157 | 0.0123 |
| ocu-cirR-novel-15941 | ENSOCUG00000001751 | RND3 | 0.0389 |
| ocu-cirR-novel-15941 | ENSOCUG00000002835 | CTGF | 0.0370 |
| ocu-cirR-novel-15941 | ENSOCUG00000003319 | HLA-DPA1 | 0.0471 |
| ocu-cirR-novel-15941 | ENSOCUG00000004632 | CA12 | 0.0080 |
| ocu-cirR-novel-15941 | ENSOCUG00000006632 | MREG | 0.0314 |
| ocu-cirR-novel-15941 | ENSOCUG00000006719 | LPCAT2 | 0.0471 |
| ocu-cirR-novel-15941 | ENSOCUG00000006800 | ATP10B | 0.0186 |
| ocu-cirR-novel-15941 | ENSOCUG00000010189 | LEP | 0.0034 |
| ocu-cirR-novel-15941 | ENSOCUG00000011530 | PHF2 | 0.0090 |
| ocu-cirR-novel-15941 | ENSOCUG00000012338 | DDX3X | 0.0134 |
| ocu-cirR-novel-15941 | ENSOCUG00000013567 | GRN | 0.0246 |
| ocu-cirR-novel-15941 | ENSOCUG00000015523 | C11orf63 | 0.0090 |
| ocu-cirR-novel-15941 | ENSOCUG00000016906 | . | 0.0026 |
| ocu-cirR-novel-15941 | ENSOCUG00000017023 | ATF1 | 0.0314 |
| ocu-cirR-novel-15960 | ENSOCUG00000010583 | FBP2 | 0.0247 |
| ocu-cirR-novel-15960 | ENSOCUG00000021506 | FOS | 0.0370 |
| ocu-cirR-novel-15960 | ENSOCUG00000023599 | APOD | 0.0247 |
| ocu-cirR-novel-15993 | ENSOCUG00000000930 | KIF15 | 0.0031 |
| ocu-cirR-novel-15993 | ENSOCUG00000001553 | LIPA | 0.0019 |
| ocu-cirR-novel-15993 | ENSOCUG00000001751 | RND3 | 0.0139 |
| ocu-cirR-novel-15993 | ENSOCUG00000001819 | ANAPC10 | 0.0003 |
| ocu-cirR-novel-15993 | ENSOCUG00000002254 | DNMBP | 0.0204 |
| ocu-cirR-novel-15993 | ENSOCUG00000003294 | WDR26 | 0.0204 |
| ocu-cirR-novel-15993 | ENSOCUG00000003715 | LAX1 | 0.0204 |
| ocu-cirR-novel-15993 | ENSOCUG00000004632 | CA12 | 0.0420 |
| ocu-cirR-novel-15993 | ENSOCUG00000005294 | RRAGD | 0.0031 |
| ocu-cirR-novel-15993 | ENSOCUG00000005883 | GPR87 | 0.0204 |
| ocu-cirR-novel-15993 | ENSOCUG00000006194 | KYNU | 0.0204 |
| ocu-cirR-novel-15993 | ENSOCUG00000006632 | MREG | 0.0111 |
| ocu-cirR-novel-15993 | ENSOCUG00000006697 | HK1 | 0.0241 |
| ocu-cirR-novel-15993 | ENSOCUG00000006719 | LPCAT2 | 0.0170 |
| ocu-cirR-novel-15993 | ENSOCUG00000008210 | CCR9 | 0.0111 |
| ocu-cirR-novel-15993 | ENSOCUG00000008568 | ANKRD28 | 0.0241 |
| ocu-cirR-novel-15993 | ENSOCUG00000008575 | LOC100125981 | 0.0281 |
| ocu-cirR-novel-15993 | ENSOCUG00000008983 | CTCF | 0.0170 |
| ocu-cirR-novel-15993 | ENSOCUG00000009065 | HNMT | 0.0065 |
| ocu-cirR-novel-15993 | ENSOCUG00000009195 | CLEC4E | 0.0019 |
| ocu-cirR-novel-15993 | ENSOCUG00000009929 | C3orf52 | 0.0139 |
| ocu-cirR-novel-15993 | ENSOCUG00000010009 | EPX | 0.0472 |
| ocu-cirR-novel-15993 | ENSOCUG00000011780 | CYFIP2 | 0.0139 |
| ocu-cirR-novel-15993 | ENSOCUG00000012034 | RBM47 | 0.0139 |
| ocu-cirR-novel-15993 | ENSOCUG00000012103 | FAT2 | 0.0241 |
| ocu-cirR-novel-15993 | ENSOCUG00000012457 | GOLGB1 | 0.0204 |
| ocu-cirR-novel-15993 | ENSOCUG00000012939 | ENPP5 | 0.0065 |
| ocu-cirR-novel-15993 | ENSOCUG00000013111 | PCOLCE2 | 0.0370 |
| ocu-cirR-novel-15993 | ENSOCUG00000013449 | RBPJ | 0.0241 |
| ocu-cirR-novel-15993 | ENSOCUG00000013498 | ADAMTS4 | 0.0472 |
| ocu-cirR-novel-15993 | ENSOCUG00000013696 | BICC1 | 0.0241 |
| ocu-cirR-novel-15993 | ENSOCUG00000013725 | FAM20A | 0.0420 |
| ocu-cirR-novel-15993 | ENSOCUG00000014332 | SHF | 0.0019 |
| ocu-cirR-novel-15993 | ENSOCUG00000014811 | KIAA1109 | 0.0031 |
| ocu-cirR-novel-15993 | ENSOCUG00000015410 | NDC80 | 0.0139 |
| ocu-cirR-novel-15993 | ENSOCUG00000015482 | TMEM241 | 0.0324 |
| ocu-cirR-novel-15993 | ENSOCUG00000015619 | CALB2 | 0.0019 |
| ocu-cirR-novel-15993 | ENSOCUG00000016963 | GNPDA1 | 0.0370 |
| ocu-cirR-novel-15993 | ENSOCUG00000021706 | TRAV9-2 | 0.0003 |
| ocu-cirR-novel-15993 | ENSOCUG00000022575 | VTN | 0.0086 |
| ocu-cirR-novel-15993 | ENSOCUG00000023896 | E2F2 | 0.0170 |
| ocu-cirR-novel-15993 | ENSOCUG00000024789 | LPAR3 | 0.0281 |
| ocu-cirR-novel-15993 | ENSOCUG00000025119 | RELT | 0.0170 |
| ocu-cirR-novel-15993 | ENSOCUG00000026691 | SLC31A2 | 0.0170 |
| ocu-cirR-novel-15993 | ENSOCUG00000027626 | CD247 | 0.0111 |
| ocu-cirR-novel-15993 | ENSOCUG00000029216 | C17H15orf48 | 0.0139 |
| ocu-cirR-novel-16001 | ENSOCUG00000004738 | STK31 | 0.0494 |
| ocu-cirR-novel-16001 | ENSOCUG00000007839 | IL6ST | 0.0494 |
| ocu-cirR-novel-16001 | ENSOCUG00000010104 | FN1 | 0.0494 |
| ocu-cirR-novel-16001 | ENSOCUG00000013454 | PRDM5 | 0.0259 |
| ocu-cirR-novel-16006 | ENSOCUG00000001853 | SLC15A2 | 0.0204 |
| ocu-cirR-novel-16006 | ENSOCUG00000002924 | LGALS3 | 0.0420 |
| ocu-cirR-novel-16006 | ENSOCUG00000003186 | HPRT1 | 0.0324 |
| ocu-cirR-novel-16006 | ENSOCUG00000009065 | HNMT | 0.0065 |
| ocu-cirR-novel-16006 | ENSOCUG00000012432 | HS3ST3B1 | 0.0241 |
| ocu-cirR-novel-16006 | ENSOCUG00000014225 | SLCO2B1 | 0.0247 |
| ocu-cirR-novel-16006 | ENSOCUG00000024081 | CD63 | 0.0491 |
| ocu-cirR-novel-16006 | ENSOCUG00000024887 | CAPRIN1 | 0.0247 |
| ocu-cirR-novel-16006 | ENSOCUG00000029068 | ZNF383 | 0.0491 |
| ocu-cirR-novel-16092 | ENSOCUG00000007145 | KIAA0586 | 0.0491 |
| ocu-cirR-novel-16123 | ENSOCUG00000004738 | STK31 | 0.0370 |
| ocu-cirR-novel-16123 | ENSOCUG00000007839 | IL6ST | 0.0370 |
| ocu-cirR-novel-16123 | ENSOCUG00000008329 | ADAMTS19 | 0.0090 |
| ocu-cirR-novel-16123 | ENSOCUG00000009863 | PRKCQ | 0.0471 |
| ocu-cirR-novel-16123 | ENSOCUG00000025830 | CAPN5 | 0.0471 |
| ocu-cirR-novel-16123 | ENSOCUG00000027815 | LOC100338913 | 0.0389 |
| ocu-cirR-novel-16228 | ENSOCUG00000001542 | RIPK2 | 0.0370 |
| ocu-cirR-novel-16228 | ENSOCUG00000002945 | MEOX1 | 0.0186 |
| ocu-cirR-novel-16228 | ENSOCUG00000003792 | THBS1 | 0.0246 |
| ocu-cirR-novel-16228 | ENSOCUG00000004337 | SMARCD2 | 0.0114 |
| ocu-cirR-novel-16228 | ENSOCUG00000008167 | CALU | 0.0246 |
| ocu-cirR-novel-16228 | ENSOCUG00000010200 | PMPCB | 0.0009 |
| ocu-cirR-novel-16228 | ENSOCUG00000010671 | SH2D2A | 0.0090 |
| ocu-cirR-novel-16228 | ENSOCUG00000010772 | DCHS2 | 0.0246 |
| ocu-cirR-novel-16228 | ENSOCUG00000015897 | ASF1A | 0.0090 |
| ocu-cirR-novel-16228 | ENSOCUG00000016477 | SELP | 0.0314 |
| ocu-cirR-novel-16228 | ENSOCUG00000016706 | KIF11 | 0.0028 |
| ocu-cirR-novel-16228 | ENSOCUG00000017386 | RPL30 | 0.0370 |
| ocu-cirR-novel-16228 | ENSOCUG00000017498 | . | 0.0090 |
| ocu-cirR-novel-16228 | ENSOCUG00000022519 | MXD3 | 0.0208 |
| ocu-cirR-novel-16228 | ENSOCUG00000026630 | ITGA9 | 0.0010 |
| ocu-cirR-novel-16228 | ENSOCUG00000029742 | MAP1LC3C | 0.0389 |
| ocu-cirR-novel-16238 | ENSOCUG00000000930 | KIF15 | 0.0286 |
| ocu-cirR-novel-16238 | ENSOCUG00000007482 | CX3CL1 | 0.0286 |
| ocu-cirR-novel-16238 | ENSOCUG00000009701 | CSF3 | 0.0176 |
| ocu-cirR-novel-16238 | ENSOCUG00000016432 | OLFM4 | 0.0417 |
| ocu-cirR-novel-16268 | ENSOCUG00000009932 | ESCO2 | 0.0370 |
| ocu-cirR-novel-16327 | ENSOCUG00000001542 | RIPK2 | 0.0123 |
| ocu-cirR-novel-16327 | ENSOCUG00000005891 | ELAVL2 | 0.0494 |
| ocu-cirR-novel-16327 | ENSOCUG00000009701 | CSF3 | 0.0494 |
| ocu-cirR-novel-16327 | ENSOCUG00000016706 | KIF11 | 0.0370 |
| ocu-cirR-novel-16327 | ENSOCUG00000022391 | . | 0.0494 |
| ocu-cirR-novel-16351 | ENSOCUG00000002898 | FBLN7 | 0.0043 |
| ocu-cirR-novel-16351 | ENSOCUG00000005263 | TOX | 0.0186 |
| ocu-cirR-novel-16351 | ENSOCUG00000006540 | PLCB2 | 0.0028 |
| ocu-cirR-novel-16351 | ENSOCUG00000007702 | TBC1D10C | 0.0014 |
| ocu-cirR-novel-16351 | ENSOCUG00000008210 | CCR9 | 0.0314 |
| ocu-cirR-novel-16351 | ENSOCUG00000009585 | SRC | 0.0090 |
| ocu-cirR-novel-16351 | ENSOCUG00000010436 | LEF1 | 0.0134 |
| ocu-cirR-novel-16351 | ENSOCUG00000015492 | GLP2R | 0.0186 |
| ocu-cirR-novel-16351 | ENSOCUG00000016959 | CRYBG2 | 0.0471 |
| ocu-cirR-novel-16351 | ENSOCUG00000021388 | P2RX1 | 0.0389 |
| ocu-cirR-novel-16351 | ENSOCUG00000027241 | MFAP3 | 0.0246 |
| ocu-cirR-novel-16351 | ENSOCUG00000027275 | TM4SF1 | 0.0055 |
| ocu-cirR-novel-16351 | ENSOCUG00000029541 | ZNF157 | 0.0370 |
| ocu-cirR-novel-16367 | ENSOCUG00000009190 | HEXA | 0.0491 |
| ocu-cirR-novel-16367 | ENSOCUG00000013137 | CETP | 0.0491 |
| ocu-cirR-novel-16367 | ENSOCUG00000016500 | . | 0.0491 |
| ocu-cirR-novel-16367 | ENSOCUG00000024349 | LOC100337909 | 0.0204 |
| ocu-cirR-novel-16458 | ENSOCUG00000001165 | RANBP2 | 0.0247 |
| ocu-cirR-novel-16458 | ENSOCUG00000003850 | PTCRA | 0.0370 |
| ocu-cirR-novel-16458 | ENSOCUG00000004244 | KLF12 | 0.0370 |
| ocu-cirR-novel-16458 | ENSOCUG00000024081 | CD63 | 0.0247 |
| ocu-cirR-novel-16458 | ENSOCUG00000024139 | TRA2A | 0.0123 |
| ocu-cirR-novel-16617 | ENSOCUG00000029337 | MMP3 | 0.0494 |
| ocu-cirR-novel-16618 | ENSOCUG00000004617 | ANKRD22 | 0.0417 |
| ocu-cirR-novel-16618 | ENSOCUG00000007702 | TBC1D10C | 0.0228 |
| ocu-cirR-novel-16618 | ENSOCUG00000017019 | CDH11 | 0.0259 |
| ocu-cirR-novel-16747 | ENSOCUG00000001590 | KLC4 | 0.0046 |
| ocu-cirR-novel-16747 | ENSOCUG00000003186 | HPRT1 | 0.0324 |
| ocu-cirR-novel-16747 | ENSOCUG00000003209 | CCBE1 | 0.0324 |
| ocu-cirR-novel-16747 | ENSOCUG00000014225 | SLCO2B1 | 0.0247 |
| ocu-cirR-novel-16747 | ENSOCUG00000014338 | ABCA5 | 0.0019 |
| ocu-cirR-novel-16747 | ENSOCUG00000017700 | PLS1 | 0.0247 |
| ocu-cirR-novel-16747 | ENSOCUG00000023681 | TSPAN18 | 0.0204 |
| ocu-cirR-novel-16747 | ENSOCUG00000024081 | CD63 | 0.0491 |
| ocu-cirR-novel-16747 | ENSOCUG00000024887 | CAPRIN1 | 0.0247 |
| ocu-cirR-novel-16747 | ENSOCUG00000025121 | ALDH6A1 | 0.0204 |
| ocu-cirR-novel-16747 | ENSOCUG00000026966 | SASH3 | 0.0065 |
| ocu-cirR-novel-16747 | ENSOCUG00000026988 | CHI3L1 | 0.0170 |
| ocu-cirR-novel-16747 | ENSOCUG00000029068 | ZNF383 | 0.0491 |
| ocu-cirR-novel-16792 | ENSOCUG00000000560 | HEYL | 0.0420 |
| ocu-cirR-novel-16792 | ENSOCUG00000001104 | RNF167 | 0.0281 |
| ocu-cirR-novel-16792 | ENSOCUG00000001751 | RND3 | 0.0139 |
| ocu-cirR-novel-16792 | ENSOCUG00000002835 | CTGF | 0.0247 |
| ocu-cirR-novel-16792 | ENSOCUG00000004494 | THSD7B | 0.0491 |
| ocu-cirR-novel-16792 | ENSOCUG00000006910 | CDCP1 | 0.0472 |
| ocu-cirR-novel-16792 | ENSOCUG00000009047 | FCRL2 | 0.0491 |
| ocu-cirR-novel-16792 | ENSOCUG00000013078 | FAXDC2 | 0.0472 |
| ocu-cirR-novel-16792 | ENSOCUG00000014252 | MAPKAPK2 | 0.0241 |
| ocu-cirR-novel-16792 | ENSOCUG00000017023 | ATF1 | 0.0111 |
| ocu-cirR-novel-16792 | ENSOCUG00000023285 | TROAP | 0.0491 |
| ocu-cirR-novel-16792 | ENSOCUG00000027626 | CD247 | 0.0111 |
| ocu-cirR-novel-16819 | ENSOCUG00000000575 | HAVCR1 | 0.0491 |
| ocu-cirR-novel-16819 | ENSOCUG00000001853 | SLC15A2 | 0.0204 |
| ocu-cirR-novel-16819 | ENSOCUG00000002924 | LGALS3 | 0.0420 |
| ocu-cirR-novel-16819 | ENSOCUG00000005439 | CCNJL | 0.0281 |
| ocu-cirR-novel-16819 | ENSOCUG00000007145 | KIAA0586 | 0.0491 |
| ocu-cirR-novel-16819 | ENSOCUG00000008167 | CALU | 0.0086 |
| ocu-cirR-novel-16819 | ENSOCUG00000009241 | RAB11FIP2 | 0.0324 |
| ocu-cirR-novel-16819 | ENSOCUG00000009761 | TMEM71 | 0.0491 |
| ocu-cirR-novel-16819 | ENSOCUG00000010200 | PMPCB | 0.0491 |
| ocu-cirR-novel-16819 | ENSOCUG00000010636 | XPNPEP2 | 0.0491 |
| ocu-cirR-novel-16819 | ENSOCUG00000010887 | RPS6KA1 | 0.0204 |
| ocu-cirR-novel-16819 | ENSOCUG00000012578 | CAMK2G | 0.0086 |
| ocu-cirR-novel-16819 | ENSOCUG00000015651 | ATG3 | 0.0204 |
| ocu-cirR-novel-16819 | ENSOCUG00000016268 | RCN1 | 0.0420 |
| ocu-cirR-novel-16819 | ENSOCUG00000017386 | RPL30 | 0.0247 |
| ocu-cirR-novel-16819 | ENSOCUG00000024225 | GMPPA | 0.0491 |
| ocu-cirR-novel-16997 | ENSOCUG00000000560 | HEYL | 0.0420 |
| ocu-cirR-novel-16997 | ENSOCUG00000001142 | SNX13 | 0.0491 |
| ocu-cirR-novel-16997 | ENSOCUG00000003863 | CD74 | 0.0491 |
| ocu-cirR-novel-16997 | ENSOCUG00000004035 | IRF6 | 0.0139 |
| ocu-cirR-novel-16997 | ENSOCUG00000004293 | EPS15 | 0.0491 |
| ocu-cirR-novel-16997 | ENSOCUG00000006357 | ADAMTS12 | 0.0204 |
| ocu-cirR-novel-16997 | ENSOCUG00000007335 | HS3ST2 | 0.0491 |
| ocu-cirR-novel-16997 | ENSOCUG00000007702 | TBC1D10C | 0.0139 |
| ocu-cirR-novel-16997 | ENSOCUG00000007892 | FBXL4 | 0.0491 |
| ocu-cirR-novel-16997 | ENSOCUG00000008137 | MLLT3 | 0.0491 |
| ocu-cirR-novel-16997 | ENSOCUG00000008191 | GABRP | 0.0065 |
| ocu-cirR-novel-16997 | ENSOCUG00000009055 | CD69 | 0.0491 |
| ocu-cirR-novel-16997 | ENSOCUG00000009190 | HEXA | 0.0491 |
| ocu-cirR-novel-16997 | ENSOCUG00000009261 | TMEM40 | 0.0241 |
| ocu-cirR-novel-16997 | ENSOCUG00000009504 | CD80 | 0.0491 |
| ocu-cirR-novel-16997 | ENSOCUG00000011298 | FAM83D | 0.0247 |
| ocu-cirR-novel-16997 | ENSOCUG00000013111 | PCOLCE2 | 0.0370 |
| ocu-cirR-novel-16997 | ENSOCUG00000013564 | TRIM63 | 0.0491 |
| ocu-cirR-novel-16997 | ENSOCUG00000015599 | GEMIN6 | 0.0491 |
| ocu-cirR-novel-16997 | ENSOCUG00000016368 | TMEM14C | 0.0491 |
| ocu-cirR-novel-16997 | ENSOCUG00000016959 | CRYBG2 | 0.0170 |
| ocu-cirR-novel-16997 | ENSOCUG00000024789 | LPAR3 | 0.0281 |
| ocu-cirR-novel-16997 | ENSOCUG00000025012 | ANGPT4 | 0.0046 |
| ocu-cirR-novel-16997 | ENSOCUG00000026567 | RLA-DMB | 0.0491 |
| ocu-cirR-novel-16997 | ENSOCUG00000029541 | ZNF157 | 0.0247 |
| ocu-cirR-novel-17032 | ENSOCUG00000004035 | IRF6 | 0.0389 |
| ocu-cirR-novel-17032 | ENSOCUG00000004136 | SEPT-7 | 0.0314 |
| ocu-cirR-novel-17032 | ENSOCUG00000006800 | ATP10B | 0.0186 |
| ocu-cirR-novel-17032 | ENSOCUG00000007355 | DSG2 | 0.0055 |
| ocu-cirR-novel-17032 | ENSOCUG00000008191 | GABRP | 0.0186 |
| ocu-cirR-novel-17032 | ENSOCUG00000009828 | ARPP21 | 0.0028 |
| ocu-cirR-novel-17032 | ENSOCUG00000009863 | PRKCQ | 0.0471 |
| ocu-cirR-novel-17032 | ENSOCUG00000010436 | LEF1 | 0.0134 |
| ocu-cirR-novel-17032 | ENSOCUG00000011550 | CD86 | 0.0055 |
| ocu-cirR-novel-17032 | ENSOCUG00000013111 | PCOLCE2 | 0.0066 |
| ocu-cirR-novel-17032 | ENSOCUG00000015138 | C1QC | 0.0055 |
| ocu-cirR-novel-17032 | ENSOCUG00000015492 | GLP2R | 0.0186 |
| ocu-cirR-novel-17032 | ENSOCUG00000029380 | ZNF311 | 0.0090 |
| ocu-cirR-novel-17032 | ENSOCUG00000029541 | ZNF157 | 0.0370 |
| ocu-cirR-novel-17059 | ENSOCUG00000005822 | MROH8 | 0.0107 |
| ocu-cirR-novel-17059 | ENSOCUG00000008568 | ANKRD28 | 0.0121 |
| ocu-cirR-novel-17059 | ENSOCUG00000011085 | CTSS | 0.0055 |
| ocu-cirR-novel-17059 | ENSOCUG00000013449 | RBPJ | 0.0121 |
| ocu-cirR-novel-17059 | ENSOCUG00000013725 | FAM20A | 0.0276 |
| ocu-cirR-novel-17059 | ENSOCUG00000014338 | ABCA5 | 0.0107 |
| ocu-cirR-novel-17059 | ENSOCUG00000017700 | PLS1 | 0.0494 |
| ocu-cirR-novel-17059 | ENSOCUG00000022021 | IL22RA2 | 0.0107 |
| ocu-cirR-novel-17059 | ENSOCUG00000026691 | SLC31A2 | 0.0071 |
| ocu-cirR-novel-17059 | ENSOCUG00000026966 | SASH3 | 0.0357 |
| ocu-cirR-novel-17059 | ENSOCUG00000029337 | MMP3 | 0.0494 |
| ocu-cirR-novel-17142 | ENSOCUG00000009077 | MS4A2 | 0.0491 |
| ocu-cirR-novel-17142 | ENSOCUG00000017563 | AKAP1 | 0.0046 |
| ocu-cirR-novel-17381 | ENSOCUG00000007892 | FBXL4 | 0.0247 |
| ocu-cirR-novel-17381 | ENSOCUG00000009212 | CD1A | 0.0494 |
| ocu-cirR-novel-17381 | ENSOCUG00000009322 | MCOLN2 | 0.0494 |
| ocu-cirR-novel-17381 | ENSOCUG00000009402 | USP32 | 0.0494 |
| ocu-cirR-novel-17381 | ENSOCUG00000016465 | TP63 | 0.0494 |
| ocu-cirR-novel-17381 | ENSOCUG00000025244 | IGFBP7 | 0.0123 |
| ocu-cirR-novel-17488 | ENSOCUG00000000560 | HEYL | 0.0276 |
| ocu-cirR-novel-17488 | ENSOCUG00000001250 | EDNRA | 0.0052 |
| ocu-cirR-novel-17488 | ENSOCUG00000002254 | DNMBP | 0.0094 |
| ocu-cirR-novel-17488 | ENSOCUG00000002898 | FBLN7 | 0.0153 |
| ocu-cirR-novel-17488 | ENSOCUG00000003089 | PRG3 | 0.0494 |
| ocu-cirR-novel-17488 | ENSOCUG00000003276 | PAX1 | 0.0019 |
| ocu-cirR-novel-17488 | ENSOCUG00000003419 | RELL1 | 0.0467 |
| ocu-cirR-novel-17488 | ENSOCUG00000003863 | CD74 | 0.0019 |
| ocu-cirR-novel-17488 | ENSOCUG00000004136 | SEPT-7 | 0.0037 |
| ocu-cirR-novel-17488 | ENSOCUG00000006325 | GALM | 0.0259 |
| ocu-cirR-novel-17488 | ENSOCUG00000006800 | ATP10B | 0.0357 |
| ocu-cirR-novel-17488 | ENSOCUG00000006970 | SLC44A4 | 0.0494 |
| ocu-cirR-novel-17488 | ENSOCUG00000008464 | PRKG2 | 0.0037 |
| ocu-cirR-novel-17488 | ENSOCUG00000009751 | HCK | 0.0176 |
| ocu-cirR-novel-17488 | ENSOCUG00000009866 | ANKRD29 | 0.0276 |
| ocu-cirR-novel-17488 | ENSOCUG00000009918 | SCARA3 | 0.0259 |
| ocu-cirR-novel-17488 | ENSOCUG00000010436 | LEF1 | 0.0259 |
| ocu-cirR-novel-17488 | ENSOCUG00000011410 | CAPSL | 0.0176 |
| ocu-cirR-novel-17488 | ENSOCUG00000015492 | GLP2R | 0.0357 |
| ocu-cirR-novel-17488 | ENSOCUG00000016268 | RCN1 | 0.0276 |
| ocu-cirR-novel-17488 | ENSOCUG00000016465 | TP63 | 0.0107 |
| ocu-cirR-novel-17488 | ENSOCUG00000016727 | NELL2 | 0.0357 |
| ocu-cirR-novel-17488 | ENSOCUG00000017240 | CDC5L | 0.0176 |
| ocu-cirR-novel-17488 | ENSOCUG00000022391 | . | 0.0107 |
| ocu-cirR-novel-17488 | ENSOCUG00000023078 | . | 0.0189 |
| ocu-cirR-novel-17488 | ENSOCUG00000025244 | IGFBP7 | 0.0494 |
| ocu-cirR-novel-17488 | ENSOCUG00000027241 | MFAP3 | 0.0467 |
| ocu-cirR-novel-17488 | ENSOCUG00000029154 | . | 0.0276 |
| ocu-cirR-novel-17488 | ENSOCUG00000029541 | ZNF157 | 0.0494 |
| ocu-cirR-novel-17488 | ENSOCUG00000029742 | MAP1LC3C | 0.0052 |
| ocu-cirR-novel-17713 | ENSOCUG00000000560 | HEYL | 0.0276 |
| ocu-cirR-novel-17713 | ENSOCUG00000001590 | KLC4 | 0.0259 |
| ocu-cirR-novel-17713 | ENSOCUG00000001853 | SLC15A2 | 0.0094 |
| ocu-cirR-novel-17713 | ENSOCUG00000003792 | THBS1 | 0.0467 |
| ocu-cirR-novel-17713 | ENSOCUG00000005316 | MAPKAPK3 | 0.0052 |
| ocu-cirR-novel-17713 | ENSOCUG00000007145 | KIAA0586 | 0.0019 |
| ocu-cirR-novel-17713 | ENSOCUG00000007265 | TREM2 | 0.0494 |
| ocu-cirR-novel-17713 | ENSOCUG00000008167 | CALU | 0.0467 |
| ocu-cirR-novel-17713 | ENSOCUG00000008474 | CD2 | 0.0259 |
| ocu-cirR-novel-17713 | ENSOCUG00000009866 | ANKRD29 | 0.0276 |
| ocu-cirR-novel-17713 | ENSOCUG00000009869 | LAMA3 | 0.0357 |
| ocu-cirR-novel-17713 | ENSOCUG00000010551 | SLC7A3 | 0.0494 |
| ocu-cirR-novel-17713 | ENSOCUG00000010636 | XPNPEP2 | 0.0019 |
| ocu-cirR-novel-17713 | ENSOCUG00000011080 | GALNT7 | 0.0357 |
| ocu-cirR-novel-17713 | ENSOCUG00000011937 | KIF3A | 0.0467 |
| ocu-cirR-novel-17713 | ENSOCUG00000012328 | RNF128 | 0.0494 |
| ocu-cirR-novel-17713 | ENSOCUG00000012400 | PTH1R | 0.0494 |
| ocu-cirR-novel-17713 | ENSOCUG00000012455 | MDFIC | 0.0357 |
| ocu-cirR-novel-17713 | ENSOCUG00000012578 | CAMK2G | 0.0467 |
| ocu-cirR-novel-17713 | ENSOCUG00000015897 | ASF1A | 0.0176 |
| ocu-cirR-novel-17713 | ENSOCUG00000016831 | SLC27A2 | 0.0494 |
| ocu-cirR-novel-17713 | ENSOCUG00000017386 | RPL30 | 0.0494 |
| ocu-cirR-novel-17713 | ENSOCUG00000017498 | . | 0.0176 |
| ocu-cirR-novel-17713 | ENSOCUG00000021497 | ARHGAP19 | 0.0153 |
| ocu-cirR-novel-17713 | ENSOCUG00000024349 | LOC100337909 | 0.0094 |
| ocu-cirR-novel-17728 | ENSOCUG00000002924 | LGALS3 | 0.0420 |
| ocu-cirR-novel-17728 | ENSOCUG00000010200 | PMPCB | 0.0491 |
| ocu-cirR-novel-17728 | ENSOCUG00000011573 | LOC100343709 | 0.0491 |
| ocu-cirR-novel-17728 | ENSOCUG00000016650 | DEPDC1B | 0.0491 |
| ocu-cirR-novel-17728 | ENSOCUG00000026206 | CCL4 | 0.0491 |
| ocu-cirR-novel-17762 | ENSOCUG00000004738 | STK31 | 0.0123 |
| ocu-cirR-novel-17762 | ENSOCUG00000007839 | IL6ST | 0.0123 |
| ocu-cirR-novel-17762 | ENSOCUG00000009761 | TMEM71 | 0.0247 |
| ocu-cirR-novel-17762 | ENSOCUG00000010192 | CDH5 | 0.0247 |
| ocu-cirR-novel-17762 | ENSOCUG00000016368 | TMEM14C | 0.0247 |
| ocu-cirR-novel-17858 | ENSOCUG00000001553 | LIPA | 0.0494 |
| ocu-cirR-novel-17858 | ENSOCUG00000010104 | FN1 | 0.0123 |
| ocu-cirR-novel-17858 | ENSOCUG00000011573 | LOC100343709 | 0.0247 |
| ocu-cirR-novel-17858 | ENSOCUG00000014003 | BUB1 | 0.0247 |
| ocu-cirR-novel-17858 | ENSOCUG00000016706 | KIF11 | 0.0370 |
| ocu-cirR-novel-17858 | ENSOCUG00000016942 | ALDH1A2 | 0.0247 |
| ocu-cirR-novel-17858 | ENSOCUG00000017308 | FLOT1 | 0.0494 |
| ocu-cirR-novel-17858 | ENSOCUG00000023836 | NEFL | 0.0370 |
| ocu-cirR-novel-17860 | ENSOCUG00000001553 | LIPA | 0.0494 |
| ocu-cirR-novel-17860 | ENSOCUG00000010104 | FN1 | 0.0123 |
| ocu-cirR-novel-17860 | ENSOCUG00000011573 | LOC100343709 | 0.0247 |
| ocu-cirR-novel-17860 | ENSOCUG00000014003 | BUB1 | 0.0247 |
| ocu-cirR-novel-17860 | ENSOCUG00000016706 | KIF11 | 0.0370 |
| ocu-cirR-novel-17860 | ENSOCUG00000016942 | ALDH1A2 | 0.0247 |
| ocu-cirR-novel-17860 | ENSOCUG00000017308 | FLOT1 | 0.0494 |
| ocu-cirR-novel-17860 | ENSOCUG00000023836 | NEFL | 0.0370 |
| ocu-cirR-novel-17879 | ENSOCUG00000001481 | RIPOR2 | 0.0259 |
| ocu-cirR-novel-17879 | ENSOCUG00000001853 | SLC15A2 | 0.0094 |
| ocu-cirR-novel-17879 | ENSOCUG00000003209 | CCBE1 | 0.0189 |
| ocu-cirR-novel-17879 | ENSOCUG00000004035 | IRF6 | 0.0052 |
| ocu-cirR-novel-17879 | ENSOCUG00000004136 | SEPT-7 | 0.0037 |
| ocu-cirR-novel-17879 | ENSOCUG00000006350 | SLC12A4 | 0.0055 |
| ocu-cirR-novel-17879 | ENSOCUG00000006357 | ADAMTS12 | 0.0094 |
| ocu-cirR-novel-17879 | ENSOCUG00000007265 | TREM2 | 0.0494 |
| ocu-cirR-novel-17879 | ENSOCUG00000007482 | CX3CL1 | 0.0176 |
| ocu-cirR-novel-17879 | ENSOCUG00000008142 | TCF7L2 | 0.0055 |
| ocu-cirR-novel-17879 | ENSOCUG00000008474 | CD2 | 0.0259 |
| ocu-cirR-novel-17879 | ENSOCUG00000009241 | RAB11FIP2 | 0.0189 |
| ocu-cirR-novel-17879 | ENSOCUG00000009261 | TMEM40 | 0.0121 |
| ocu-cirR-novel-17879 | ENSOCUG00000010061 | PLEKHG4 | 0.0467 |
| ocu-cirR-novel-17879 | ENSOCUG00000010095 | ZNF18 | 0.0189 |
| ocu-cirR-novel-17879 | ENSOCUG00000010551 | SLC7A3 | 0.0494 |
| ocu-cirR-novel-17879 | ENSOCUG00000011267 | ARHGEF9 | 0.0037 |
| ocu-cirR-novel-17879 | ENSOCUG00000011550 | CD86 | 0.0107 |
| ocu-cirR-novel-17879 | ENSOCUG00000012328 | RNF128 | 0.0494 |
| ocu-cirR-novel-17879 | ENSOCUG00000012400 | PTH1R | 0.0494 |
| ocu-cirR-novel-17879 | ENSOCUG00000012578 | CAMK2G | 0.0467 |
| ocu-cirR-novel-17879 | ENSOCUG00000015138 | C1QC | 0.0107 |
| ocu-cirR-novel-17879 | ENSOCUG00000015492 | GLP2R | 0.0016 |
| ocu-cirR-novel-17879 | ENSOCUG00000016268 | RCN1 | 0.0276 |
| ocu-cirR-novel-17879 | ENSOCUG00000016432 | OLFM4 | 0.0259 |
| ocu-cirR-novel-17879 | ENSOCUG00000016727 | NELL2 | 0.0357 |
| ocu-cirR-novel-17879 | ENSOCUG00000016831 | SLC27A2 | 0.0494 |
| ocu-cirR-novel-17879 | ENSOCUG00000021242 | PKP1 | 0.0357 |
| ocu-cirR-novel-17879 | ENSOCUG00000021580 | FGF23 | 0.0357 |
| ocu-cirR-novel-17879 | ENSOCUG00000024225 | GMPPA | 0.0019 |
| ocu-cirR-novel-17879 | ENSOCUG00000025121 | ALDH6A1 | 0.0094 |
| ocu-cirR-novel-17879 | ENSOCUG00000027241 | MFAP3 | 0.0467 |
| ocu-cirR-novel-17879 | ENSOCUG00000029380 | ZNF311 | 0.0176 |
| ocu-cirR-novel-17879 | ENSOCUG00000029541 | ZNF157 | 0.0494 |
| ocu-cirR-novel-17880 | ENSOCUG00000000560 | HEYL | 0.0472 |
| ocu-cirR-novel-17880 | ENSOCUG00000001590 | KLC4 | 0.0304 |
| ocu-cirR-novel-17880 | ENSOCUG00000001853 | SLC15A2 | 0.0087 |
| ocu-cirR-novel-17880 | ENSOCUG00000004035 | IRF6 | 0.0262 |
| ocu-cirR-novel-17880 | ENSOCUG00000005263 | TOX | 0.0490 |
| ocu-cirR-novel-17880 | ENSOCUG00000007145 | KIAA0586 | 0.0170 |
| ocu-cirR-novel-17880 | ENSOCUG00000008137 | MLLT3 | 0.0170 |
| ocu-cirR-novel-17880 | ENSOCUG00000008474 | CD2 | 0.0304 |
| ocu-cirR-novel-17880 | ENSOCUG00000009828 | ARPP21 | 0.0471 |
| ocu-cirR-novel-17880 | ENSOCUG00000010203 | TLN1 | 0.0471 |
| ocu-cirR-novel-17880 | ENSOCUG00000010238 | PIEZO2 | 0.0304 |
| ocu-cirR-novel-17880 | ENSOCUG00000010425 | FABP5 | 0.0001 |
| ocu-cirR-novel-17880 | ENSOCUG00000011419 | NEK7 | 0.0165 |
| ocu-cirR-novel-17880 | ENSOCUG00000012578 | CAMK2G | 0.0008 |
| ocu-cirR-novel-17880 | ENSOCUG00000015020 | COL5A2 | 0.0490 |
| ocu-cirR-novel-17880 | ENSOCUG00000016959 | CRYBG2 | 0.0381 |
| ocu-cirR-novel-17880 | ENSOCUG00000017438 | SELE | 0.0165 |
| ocu-cirR-novel-17880 | ENSOCUG00000022519 | MXD3 | 0.0481 |
| ocu-cirR-novel-17880 | ENSOCUG00000024789 | LPAR3 | 0.0191 |
| ocu-cirR-novel-17880 | ENSOCUG00000029216 | C17H15orf48 | 0.0262 |
| ocu-cirR-novel-17880 | ENSOCUG00000029380 | ZNF311 | 0.0165 |
| ocu-cirR-novel-17880 | ENSOCUG00000029742 | MAP1LC3C | 0.0262 |
| ocu-cirR-novel-17934 | ENSOCUG00000000560 | HEYL | 0.0160 |
| ocu-cirR-novel-17934 | ENSOCUG00000001144 | SLC6A6 | 0.0165 |
| ocu-cirR-novel-17934 | ENSOCUG00000003209 | CCBE1 | 0.0096 |
| ocu-cirR-novel-17934 | ENSOCUG00000004136 | SEPT-7 | 0.0165 |
| ocu-cirR-novel-17934 | ENSOCUG00000006595 | UBA7 | 0.0003 |
| ocu-cirR-novel-17934 | ENSOCUG00000006637 | SLC23A2 | 0.0495 |
| ocu-cirR-novel-17934 | ENSOCUG00000006714 | MMP2 | 0.0037 |
| ocu-cirR-novel-17934 | ENSOCUG00000007482 | CX3CL1 | 0.0022 |
| ocu-cirR-novel-17934 | ENSOCUG00000007702 | TBC1D10C | 0.0228 |
| ocu-cirR-novel-17934 | ENSOCUG00000007730 | LAD1 | 0.0393 |
| ocu-cirR-novel-17934 | ENSOCUG00000008142 | TCF7L2 | 0.0134 |
| ocu-cirR-novel-17934 | ENSOCUG00000009261 | TMEM40 | 0.0495 |
| ocu-cirR-novel-17934 | ENSOCUG00000009701 | CSF3 | 0.0009 |
| ocu-cirR-novel-17934 | ENSOCUG00000009751 | HCK | 0.0417 |
| ocu-cirR-novel-17934 | ENSOCUG00000009866 | ANKRD29 | 0.0160 |
| ocu-cirR-novel-17934 | ENSOCUG00000010203 | TLN1 | 0.0134 |
| ocu-cirR-novel-17934 | ENSOCUG00000010671 | SH2D2A | 0.0417 |
| ocu-cirR-novel-17934 | ENSOCUG00000010772 | DCHS2 | 0.0113 |
| ocu-cirR-novel-17934 | ENSOCUG00000012457 | GOLGB1 | 0.0037 |
| ocu-cirR-novel-17934 | ENSOCUG00000012754 | RUBCN | 0.0043 |
| ocu-cirR-novel-17934 | ENSOCUG00000013725 | FAM20A | 0.0160 |
| ocu-cirR-novel-17934 | ENSOCUG00000014125 | PGM5 | 0.0393 |
| ocu-cirR-novel-17934 | ENSOCUG00000014252 | MAPKAPK2 | 0.0495 |
| ocu-cirR-novel-17934 | ENSOCUG00000015138 | C1QC | 0.0259 |
| ocu-cirR-novel-17934 | ENSOCUG00000015723 | SLC15A3 | 0.0417 |
| ocu-cirR-novel-17934 | ENSOCUG00000016580 | CTSB | 0.0228 |
| ocu-cirR-novel-17934 | ENSOCUG00000016706 | KIF11 | 0.0134 |
| ocu-cirR-novel-17934 | ENSOCUG00000016883 | FERMT1 | 0.0228 |
| ocu-cirR-novel-17934 | ENSOCUG00000017498 | . | 0.0417 |
| ocu-cirR-novel-17934 | ENSOCUG00000021242 | PKP1 | 0.0073 |
| ocu-cirR-novel-17934 | ENSOCUG00000021580 | FGF23 | 0.0073 |
| ocu-cirR-novel-17934 | ENSOCUG00000022391 | . | 0.0259 |
| ocu-cirR-novel-17934 | ENSOCUG00000023078 | . | 0.0096 |
| ocu-cirR-novel-17934 | ENSOCUG00000023836 | NEFL | 0.0134 |
| ocu-cirR-novel-17934 | ENSOCUG00000024358 | CDIPT | 0.0304 |
| ocu-cirR-novel-17934 | ENSOCUG00000026966 | SASH3 | 0.0073 |
| ocu-cirR-novel-17934 | ENSOCUG00000027275 | TM4SF1 | 0.0259 |
| ocu-cirR-novel-17934 | ENSOCUG00000028052 | AMOTL2 | 0.0228 |
| ocu-cirR-novel-17934 | ENSOCUG00000029742 | MAP1LC3C | 0.0228 |
| ocu-cirR-novel-18036 | ENSOCUG00000001553 | LIPA | 0.0447 |
| ocu-cirR-novel-18036 | ENSOCUG00000002254 | DNMBP | 0.0380 |
| ocu-cirR-novel-18036 | ENSOCUG00000003294 | WDR26 | 0.0075 |
| ocu-cirR-novel-18036 | ENSOCUG00000003715 | LAX1 | 0.0380 |
| ocu-cirR-novel-18036 | ENSOCUG00000004136 | SEPT-7 | 0.0369 |
| ocu-cirR-novel-18036 | ENSOCUG00000004451 | LIMS1 | 0.0380 |
| ocu-cirR-novel-18036 | ENSOCUG00000004632 | CA12 | 0.0046 |
| ocu-cirR-novel-18036 | ENSOCUG00000006632 | MREG | 0.0059 |
| ocu-cirR-novel-18036 | ENSOCUG00000006719 | LPCAT2 | 0.0037 |
| ocu-cirR-novel-18036 | ENSOCUG00000008167 | CALU | 0.0197 |
| ocu-cirR-novel-18036 | ENSOCUG00000008210 | CCR9 | 0.0059 |
| ocu-cirR-novel-18036 | ENSOCUG00000008568 | ANKRD28 | 0.0003 |
| ocu-cirR-novel-18036 | ENSOCUG00000008575 | LOC100125981 | 0.0044 |
| ocu-cirR-novel-18036 | ENSOCUG00000009195 | CLEC4E | 0.0447 |
| ocu-cirR-novel-18036 | ENSOCUG00000009701 | CSF3 | 0.0447 |
| ocu-cirR-novel-18036 | ENSOCUG00000009863 | PRKCQ | 0.0228 |
| ocu-cirR-novel-18036 | ENSOCUG00000010203 | TLN1 | 0.0134 |
| ocu-cirR-novel-18036 | ENSOCUG00000012103 | FAT2 | 0.0137 |
| ocu-cirR-novel-18036 | ENSOCUG00000012338 | DDX3X | 0.0030 |
| ocu-cirR-novel-18036 | ENSOCUG00000012754 | RUBCN | 0.0304 |
| ocu-cirR-novel-18036 | ENSOCUG00000013449 | RBPJ | 0.0137 |
| ocu-cirR-novel-18036 | ENSOCUG00000013498 | ADAMTS4 | 0.0326 |
| ocu-cirR-novel-18036 | ENSOCUG00000013725 | FAM20A | 0.0046 |
| ocu-cirR-novel-18036 | ENSOCUG00000014332 | SHF | 0.0447 |
| ocu-cirR-novel-18036 | ENSOCUG00000015138 | C1QC | 0.0447 |
| ocu-cirR-novel-18036 | ENSOCUG00000016477 | SELP | 0.0369 |
| ocu-cirR-novel-18036 | ENSOCUG00000016706 | KIF11 | 0.0134 |
| ocu-cirR-novel-18036 | ENSOCUG00000016828 | PRR14L | 0.0284 |
| ocu-cirR-novel-18036 | ENSOCUG00000017165 | LRP1B | 0.0121 |
| ocu-cirR-novel-18036 | ENSOCUG00000022391 | . | 0.0029 |
| ocu-cirR-novel-18036 | ENSOCUG00000022575 | VTN | 0.0197 |
| ocu-cirR-novel-18036 | ENSOCUG00000025121 | ALDH6A1 | 0.0380 |
| ocu-cirR-novel-18036 | ENSOCUG00000026630 | ITGA9 | 0.0059 |
| ocu-cirR-novel-18038 | ENSOCUG00000000560 | HEYL | 0.0160 |
| ocu-cirR-novel-18038 | ENSOCUG00000001144 | SLC6A6 | 0.0165 |
| ocu-cirR-novel-18038 | ENSOCUG00000001250 | EDNRA | 0.0017 |
| ocu-cirR-novel-18038 | ENSOCUG00000001387 | TDRKH | 0.0259 |
| ocu-cirR-novel-18038 | ENSOCUG00000001853 | SLC15A2 | 0.0037 |
| ocu-cirR-novel-18038 | ENSOCUG00000002254 | DNMBP | 0.0393 |
| ocu-cirR-novel-18038 | ENSOCUG00000002898 | FBLN7 | 0.0072 |
| ocu-cirR-novel-18038 | ENSOCUG00000003299 | DMRT2 | 0.0417 |
| ocu-cirR-novel-18038 | ENSOCUG00000003313 | LOX | 0.0165 |
| ocu-cirR-novel-18038 | ENSOCUG00000003863 | CD74 | 0.0046 |
| ocu-cirR-novel-18038 | ENSOCUG00000004035 | IRF6 | 0.0228 |
| ocu-cirR-novel-18038 | ENSOCUG00000004136 | SEPT-7 | 0.0010 |
| ocu-cirR-novel-18038 | ENSOCUG00000005655 | SMC2 | 0.0113 |
| ocu-cirR-novel-18038 | ENSOCUG00000006350 | SLC12A4 | 0.0134 |
| ocu-cirR-novel-18038 | ENSOCUG00000006357 | ADAMTS12 | 0.0393 |
| ocu-cirR-novel-18038 | ENSOCUG00000006498 | DSG1 | 0.0165 |
| ocu-cirR-novel-18038 | ENSOCUG00000006540 | PLCB2 | 0.0134 |
| ocu-cirR-novel-18038 | ENSOCUG00000006595 | UBA7 | 0.0053 |
| ocu-cirR-novel-18038 | ENSOCUG00000006637 | SLC23A2 | 0.0495 |
| ocu-cirR-novel-18038 | ENSOCUG00000006694 | TNLG1E | 0.0134 |
| ocu-cirR-novel-18038 | ENSOCUG00000006797 | MED12 | 0.0259 |
| ocu-cirR-novel-18038 | ENSOCUG00000007482 | CX3CL1 | 0.0417 |
| ocu-cirR-novel-18038 | ENSOCUG00000007730 | LAD1 | 0.0037 |
| ocu-cirR-novel-18038 | ENSOCUG00000008142 | TCF7L2 | 0.0134 |
| ocu-cirR-novel-18038 | ENSOCUG00000008464 | PRKG2 | 0.0010 |
| ocu-cirR-novel-18038 | ENSOCUG00000008620 | DAPL1 | 0.0417 |
| ocu-cirR-novel-18038 | ENSOCUG00000009261 | TMEM40 | 0.0495 |
| ocu-cirR-novel-18038 | ENSOCUG00000009751 | HCK | 0.0022 |
| ocu-cirR-novel-18038 | ENSOCUG00000009866 | ANKRD29 | 0.0160 |
| ocu-cirR-novel-18038 | ENSOCUG00000010061 | PLEKHG4 | 0.0113 |
| ocu-cirR-novel-18038 | ENSOCUG00000010095 | ZNF18 | 0.0096 |
| ocu-cirR-novel-18038 | ENSOCUG00000010261 | EHF | 0.0165 |
| ocu-cirR-novel-18038 | ENSOCUG00000010649 | MKX | 0.0259 |
| ocu-cirR-novel-18038 | ENSOCUG00000010887 | RPS6KA1 | 0.0393 |
| ocu-cirR-novel-18038 | ENSOCUG00000011064 | CLSTN1 | 0.0013 |
| ocu-cirR-novel-18038 | ENSOCUG00000011739 | . | 0.0165 |
| ocu-cirR-novel-18038 | ENSOCUG00000012103 | FAT2 | 0.0495 |
| ocu-cirR-novel-18038 | ENSOCUG00000012432 | HS3ST3B1 | 0.0495 |
| ocu-cirR-novel-18038 | ENSOCUG00000013333 | HS3ST3A1 | 0.0053 |
| ocu-cirR-novel-18038 | ENSOCUG00000013567 | GRN | 0.0113 |
| ocu-cirR-novel-18038 | ENSOCUG00000015166 | . | 0.0043 |
| ocu-cirR-novel-18038 | ENSOCUG00000015320 | COL12A1 | 0.0417 |
| ocu-cirR-novel-18038 | ENSOCUG00000015492 | GLP2R | 0.0073 |
| ocu-cirR-novel-18038 | ENSOCUG00000015723 | SLC15A3 | 0.0417 |
| ocu-cirR-novel-18038 | ENSOCUG00000016268 | RCN1 | 0.0160 |
| ocu-cirR-novel-18038 | ENSOCUG00000016465 | TP63 | 0.0259 |
| ocu-cirR-novel-18038 | ENSOCUG00000016727 | NELL2 | 0.0003 |
| ocu-cirR-novel-18038 | ENSOCUG00000016828 | PRR14L | 0.0366 |
| ocu-cirR-novel-18038 | ENSOCUG00000017021 | SLC30A3 | 0.0495 |
| ocu-cirR-novel-18038 | ENSOCUG00000017240 | CDC5L | 0.0417 |
| ocu-cirR-novel-18038 | ENSOCUG00000017498 | . | 0.0417 |
| ocu-cirR-novel-18038 | ENSOCUG00000021580 | FGF23 | 0.0073 |
| ocu-cirR-novel-18038 | ENSOCUG00000023681 | TSPAN18 | 0.0393 |
| ocu-cirR-novel-18038 | ENSOCUG00000024358 | CDIPT | 0.0304 |
| ocu-cirR-novel-18038 | ENSOCUG00000025119 | RELT | 0.0304 |
| ocu-cirR-novel-18038 | ENSOCUG00000025569 | CCR7 | 0.0259 |
| ocu-cirR-novel-18038 | ENSOCUG00000025830 | CAPN5 | 0.0304 |
| ocu-cirR-novel-18038 | ENSOCUG00000027241 | MFAP3 | 0.0113 |
| ocu-cirR-novel-18038 | ENSOCUG00000029154 | . | 0.0160 |
| ocu-cirR-novel-18038 | ENSOCUG00000029742 | MAP1LC3C | 0.0228 |
| ocu-cirR-novel-18121 | ENSOCUG00000003569 | HMGCS1 | 0.0491 |
| ocu-cirR-novel-18121 | ENSOCUG00000004847 | GAP43 | 0.0491 |
| ocu-cirR-novel-18121 | ENSOCUG00000006357 | ADAMTS12 | 0.0204 |
| ocu-cirR-novel-18121 | ENSOCUG00000006561 | XAF1 | 0.0491 |
| ocu-cirR-novel-18121 | ENSOCUG00000007265 | TREM2 | 0.0247 |
| ocu-cirR-novel-18121 | ENSOCUG00000010551 | SLC7A3 | 0.0247 |
| ocu-cirR-novel-18121 | ENSOCUG00000010636 | XPNPEP2 | 0.0491 |
| ocu-cirR-novel-18121 | ENSOCUG00000012328 | RNF128 | 0.0247 |
| ocu-cirR-novel-18121 | ENSOCUG00000012400 | PTH1R | 0.0247 |
| ocu-cirR-novel-18121 | ENSOCUG00000013333 | HS3ST3A1 | 0.0241 |
| ocu-cirR-novel-18121 | ENSOCUG00000015492 | GLP2R | 0.0065 |
| ocu-cirR-novel-18121 | ENSOCUG00000016005 | EXO5 | 0.0247 |
| ocu-cirR-novel-18121 | ENSOCUG00000016831 | SLC27A2 | 0.0247 |
| ocu-cirR-novel-18121 | ENSOCUG00000017111 | KIF4A | 0.0491 |
| ocu-cirR-novel-18121 | ENSOCUG00000024225 | GMPPA | 0.0491 |
| ocu-cirR-novel-18192 | ENSOCUG00000009055 | CD69 | 0.0247 |
| ocu-cirR-novel-18298 | ENSOCUG00000000560 | HEYL | 0.0080 |
| ocu-cirR-novel-18298 | ENSOCUG00000000577 | RSPRY1 | 0.0389 |
| ocu-cirR-novel-18298 | ENSOCUG00000001006 | TACR1 | 0.0314 |
| ocu-cirR-novel-18298 | ENSOCUG00000001144 | SLC6A6 | 0.0010 |
| ocu-cirR-novel-18298 | ENSOCUG00000001250 | EDNRA | 0.0389 |
| ocu-cirR-novel-18298 | ENSOCUG00000003209 | CCBE1 | 0.0053 |
| ocu-cirR-novel-18298 | ENSOCUG00000004035 | IRF6 | 0.0389 |
| ocu-cirR-novel-18298 | ENSOCUG00000004136 | SEPT-7 | 0.0314 |
| ocu-cirR-novel-18298 | ENSOCUG00000005655 | SMC2 | 0.0246 |
| ocu-cirR-novel-18298 | ENSOCUG00000006186 | CPXM1 | 0.0246 |
| ocu-cirR-novel-18298 | ENSOCUG00000006325 | GALM | 0.0134 |
| ocu-cirR-novel-18298 | ENSOCUG00000006595 | UBA7 | 0.0034 |
| ocu-cirR-novel-18298 | ENSOCUG00000006714 | MMP2 | 0.0026 |
| ocu-cirR-novel-18298 | ENSOCUG00000006986 | SCIN | 0.0186 |
| ocu-cirR-novel-18298 | ENSOCUG00000007265 | TREM2 | 0.0370 |
| ocu-cirR-novel-18298 | ENSOCUG00000007482 | CX3CL1 | 0.0001 |
| ocu-cirR-novel-18298 | ENSOCUG00000007702 | TBC1D10C | 0.0389 |
| ocu-cirR-novel-18298 | ENSOCUG00000008142 | TCF7L2 | 0.0028 |
| ocu-cirR-novel-18298 | ENSOCUG00000008474 | CD2 | 0.0134 |
| ocu-cirR-novel-18298 | ENSOCUG00000009261 | TMEM40 | 0.0034 |
| ocu-cirR-novel-18298 | ENSOCUG00000009701 | CSF3 | 0.0055 |
| ocu-cirR-novel-18298 | ENSOCUG00000009751 | HCK | 0.0090 |
| ocu-cirR-novel-18298 | ENSOCUG00000009866 | ANKRD29 | 0.0080 |
| ocu-cirR-novel-18298 | ENSOCUG00000010095 | ZNF18 | 0.0053 |
| ocu-cirR-novel-18298 | ENSOCUG00000010203 | TLN1 | 0.0028 |
| ocu-cirR-novel-18298 | ENSOCUG00000010261 | EHF | 0.0314 |
| ocu-cirR-novel-18298 | ENSOCUG00000010551 | SLC7A3 | 0.0370 |
| ocu-cirR-novel-18298 | ENSOCUG00000012034 | RBM47 | 0.0389 |
| ocu-cirR-novel-18298 | ENSOCUG00000012328 | RNF128 | 0.0370 |
| ocu-cirR-novel-18298 | ENSOCUG00000012400 | PTH1R | 0.0370 |
| ocu-cirR-novel-18298 | ENSOCUG00000012754 | RUBCN | 0.0134 |
| ocu-cirR-novel-18298 | ENSOCUG00000013725 | FAM20A | 0.0080 |
| ocu-cirR-novel-18298 | ENSOCUG00000014250 | TRDC | 0.0246 |
| ocu-cirR-novel-18298 | ENSOCUG00000014988 | COL3A1 | 0.0370 |
| ocu-cirR-novel-18298 | ENSOCUG00000015138 | C1QC | 0.0055 |
| ocu-cirR-novel-18298 | ENSOCUG00000015410 | NDC80 | 0.0389 |
| ocu-cirR-novel-18298 | ENSOCUG00000015492 | GLP2R | 0.0186 |
| ocu-cirR-novel-18298 | ENSOCUG00000015723 | SLC15A3 | 0.0090 |
| ocu-cirR-novel-18298 | ENSOCUG00000016580 | CTSB | 0.0014 |
| ocu-cirR-novel-18298 | ENSOCUG00000016727 | NELL2 | 0.0186 |
| ocu-cirR-novel-18298 | ENSOCUG00000016828 | PRR14L | 0.0156 |
| ocu-cirR-novel-18298 | ENSOCUG00000016831 | SLC27A2 | 0.0370 |
| ocu-cirR-novel-18298 | ENSOCUG00000016883 | FERMT1 | 0.0389 |
| ocu-cirR-novel-18298 | ENSOCUG00000021242 | PKP1 | 0.0186 |
| ocu-cirR-novel-18298 | ENSOCUG00000021388 | P2RX1 | 0.0389 |
| ocu-cirR-novel-18298 | ENSOCUG00000021580 | FGF23 | 0.0186 |
| ocu-cirR-novel-18298 | ENSOCUG00000022519 | MXD3 | 0.0208 |
| ocu-cirR-novel-18298 | ENSOCUG00000024358 | CDIPT | 0.0471 |
| ocu-cirR-novel-18298 | ENSOCUG00000025119 | RELT | 0.0471 |
| ocu-cirR-novel-18298 | ENSOCUG00000025512 | SPINK8 | 0.0370 |
| ocu-cirR-novel-18298 | ENSOCUG00000026966 | SASH3 | 0.0186 |
| ocu-cirR-novel-18298 | ENSOCUG00000027241 | MFAP3 | 0.0246 |
| ocu-cirR-novel-18298 | ENSOCUG00000027275 | TM4SF1 | 0.0055 |
| ocu-cirR-novel-18298 | ENSOCUG00000027603 | PDGFB | 0.0343 |
| ocu-cirR-novel-18298 | ENSOCUG00000027815 | LOC100338913 | 0.0389 |
| ocu-cirR-novel-18298 | ENSOCUG00000029541 | ZNF157 | 0.0370 |
| ocu-cirR-novel-18298 | ENSOCUG00000029569 | KRT17 | 0.0370 |
| ocu-cirR-novel-18298 | ENSOCUG00000029742 | MAP1LC3C | 0.0389 |
| ocu-cirR-novel-18415 | ENSOCUG00000001553 | LIPA | 0.0494 |
| ocu-cirR-novel-18415 | ENSOCUG00000010104 | FN1 | 0.0123 |
| ocu-cirR-novel-18415 | ENSOCUG00000011573 | LOC100343709 | 0.0247 |
| ocu-cirR-novel-18415 | ENSOCUG00000014003 | BUB1 | 0.0247 |
| ocu-cirR-novel-18415 | ENSOCUG00000016706 | KIF11 | 0.0370 |
| ocu-cirR-novel-18415 | ENSOCUG00000016942 | ALDH1A2 | 0.0247 |
| ocu-cirR-novel-18415 | ENSOCUG00000017308 | FLOT1 | 0.0494 |
| ocu-cirR-novel-18415 | ENSOCUG00000023836 | NEFL | 0.0370 |
| ocu-cirR-novel-18453 | ENSOCUG00000003999 | CAMK1G | 0.0046 |
| ocu-cirR-novel-18453 | ENSOCUG00000009863 | PRKCQ | 0.0170 |
| ocu-cirR-novel-18453 | ENSOCUG00000012363 | AGTR2 | 0.0247 |
| ocu-cirR-novel-18453 | ENSOCUG00000013567 | GRN | 0.0086 |
| ocu-cirR-novel-18453 | ENSOCUG00000014003 | BUB1 | 0.0491 |
| ocu-cirR-novel-18456 | ENSOCUG00000008307 | LOC100356376 | 0.0247 |
| ocu-cirR-novel-18456 | ENSOCUG00000010583 | FBP2 | 0.0003 |
| ocu-cirR-novel-18456 | ENSOCUG00000023599 | APOD | 0.0491 |
| ocu-cirR-novel-18487 | ENSOCUG00000001542 | RIPK2 | 0.0247 |
| ocu-cirR-novel-18487 | ENSOCUG00000002528 | LRCH4 | 0.0491 |
| ocu-cirR-novel-18487 | ENSOCUG00000006714 | MMP2 | 0.0204 |
| ocu-cirR-novel-18487 | ENSOCUG00000007702 | TBC1D10C | 0.0139 |
| ocu-cirR-novel-18487 | ENSOCUG00000010972 | LOC100328967 | 0.0031 |
| ocu-cirR-novel-18487 | ENSOCUG00000014125 | PGM5 | 0.0204 |
| ocu-cirR-novel-18487 | ENSOCUG00000021242 | PKP1 | 0.0065 |
| ocu-cirR-novel-18487 | ENSOCUG00000021497 | ARHGAP19 | 0.0281 |
| ocu-cirR-novel-18487 | ENSOCUG00000023285 | TROAP | 0.0491 |
| ocu-cirR-novel-18487 | ENSOCUG00000026630 | ITGA9 | 0.0111 |
| ocu-cirR-novel-18487 | ENSOCUG00000029068 | ZNF383 | 0.0491 |
| ocu-cirR-novel-18489 | ENSOCUG00000002871 | DNTT | 0.0259 |
| ocu-cirR-novel-18489 | ENSOCUG00000003049 | COL17A1 | 0.0494 |
| ocu-cirR-novel-18489 | ENSOCUG00000005294 | RRAGD | 0.0176 |
| ocu-cirR-novel-18489 | ENSOCUG00000006910 | CDCP1 | 0.0327 |
| ocu-cirR-novel-18489 | ENSOCUG00000011739 | . | 0.0037 |
| ocu-cirR-novel-18489 | ENSOCUG00000012403 | GDA | 0.0357 |
| ocu-cirR-novel-18489 | ENSOCUG00000014250 | TRDC | 0.0467 |
| ocu-cirR-novel-18489 | ENSOCUG00000015410 | NDC80 | 0.0052 |
| ocu-cirR-novel-18489 | ENSOCUG00000024777 | . | 0.0005 |
| ocu-cirR-novel-18489 | ENSOCUG00000025803 | LGALS9 | 0.0494 |
| ocu-cirR-novel-18489 | ENSOCUG00000027818 | . | 0.0052 |
| ocu-cirR-novel-18590 | ENSOCUG00000003209 | CCBE1 | 0.0324 |
| ocu-cirR-novel-18590 | ENSOCUG00000005883 | GPR87 | 0.0204 |
| ocu-cirR-novel-18590 | ENSOCUG00000013078 | FAXDC2 | 0.0472 |
| ocu-cirR-novel-18590 | ENSOCUG00000014225 | SLCO2B1 | 0.0247 |
| ocu-cirR-novel-18590 | ENSOCUG00000023926 | ZNF711 | 0.0491 |
| ocu-cirR-novel-18590 | ENSOCUG00000024081 | CD63 | 0.0491 |
| ocu-cirR-novel-18590 | ENSOCUG00000024887 | CAPRIN1 | 0.0247 |
| ocu-cirR-novel-18590 | ENSOCUG00000029068 | ZNF383 | 0.0491 |
| ocu-cirR-novel-18638 | ENSOCUG00000005439 | CCNJL | 0.0281 |
| ocu-cirR-novel-18638 | ENSOCUG00000005903 | SSBP3 | 0.0241 |
| ocu-cirR-novel-18638 | ENSOCUG00000007499 | LMNB1 | 0.0491 |
| ocu-cirR-novel-18638 | ENSOCUG00000008071 | . | 0.0247 |
| ocu-cirR-novel-18638 | ENSOCUG00000008575 | LOC100125981 | 0.0281 |
| ocu-cirR-novel-18638 | ENSOCUG00000010200 | PMPCB | 0.0491 |
| ocu-cirR-novel-18638 | ENSOCUG00000011573 | LOC100343709 | 0.0491 |
| ocu-cirR-novel-18638 | ENSOCUG00000012561 | SLC43A3 | 0.0019 |
| ocu-cirR-novel-18638 | ENSOCUG00000016477 | SELP | 0.0111 |
| ocu-cirR-novel-18638 | ENSOCUG00000016650 | DEPDC1B | 0.0491 |
| ocu-cirR-novel-18638 | ENSOCUG00000017111 | KIF4A | 0.0491 |
| ocu-cirR-novel-18638 | ENSOCUG00000023553 | GFI1 | 0.0247 |
| ocu-cirR-novel-18638 | ENSOCUG00000026206 | CCL4 | 0.0491 |
| ocu-cirR-novel-18638 | ENSOCUG00000026480 | AOX2 | 0.0065 |
| ocu-cirR-novel-18638 | ENSOCUG00000026630 | ITGA9 | 0.0111 |
| ocu-cirR-novel-18638 | ENSOCUG00000027471 | IL6 | 0.0491 |
| ocu-cirR-novel-18642 | ENSOCUG00000003186 | HPRT1 | 0.0189 |
| ocu-cirR-novel-18642 | ENSOCUG00000003209 | CCBE1 | 0.0189 |
| ocu-cirR-novel-18642 | ENSOCUG00000004293 | EPS15 | 0.0019 |
| ocu-cirR-novel-18642 | ENSOCUG00000005822 | MROH8 | 0.0107 |
| ocu-cirR-novel-18642 | ENSOCUG00000008191 | GABRP | 0.0357 |
| ocu-cirR-novel-18642 | ENSOCUG00000008210 | CCR9 | 0.0037 |
| ocu-cirR-novel-18642 | ENSOCUG00000008474 | CD2 | 0.0259 |
| ocu-cirR-novel-18642 | ENSOCUG00000009701 | CSF3 | 0.0107 |
| ocu-cirR-novel-18642 | ENSOCUG00000011085 | CTSS | 0.0055 |
| ocu-cirR-novel-18642 | ENSOCUG00000013725 | FAM20A | 0.0014 |
| ocu-cirR-novel-18642 | ENSOCUG00000014338 | ABCA5 | 0.0107 |
| ocu-cirR-novel-18642 | ENSOCUG00000015020 | COL5A2 | 0.0357 |
| ocu-cirR-novel-18642 | ENSOCUG00000017165 | LRP1B | 0.0176 |
| ocu-cirR-novel-18642 | ENSOCUG00000017700 | PLS1 | 0.0494 |
| ocu-cirR-novel-18642 | ENSOCUG00000021388 | P2RX1 | 0.0001 |
| ocu-cirR-novel-18642 | ENSOCUG00000022021 | IL22RA2 | 0.0107 |
| ocu-cirR-novel-18642 | ENSOCUG00000025121 | ALDH6A1 | 0.0094 |
| ocu-cirR-novel-18642 | ENSOCUG00000026966 | SASH3 | 0.0357 |
| ocu-cirR-novel-18642 | ENSOCUG00000029541 | ZNF157 | 0.0494 |
| ocu-cirR-novel-18684 | ENSOCUG00000000109 | ADAMTS9 | 0.0247 |
| ocu-cirR-novel-18684 | ENSOCUG00000003911 | CD40LG | 0.0247 |
| ocu-cirR-novel-18684 | ENSOCUG00000007499 | LMNB1 | 0.0491 |
| ocu-cirR-novel-18684 | ENSOCUG00000021747 | WDR78 | 0.0247 |
| ocu-cirR-novel-18685 | ENSOCUG00000003313 | LOX | 0.0314 |
| ocu-cirR-novel-18685 | ENSOCUG00000008071 | . | 0.0370 |
| ocu-cirR-novel-18685 | ENSOCUG00000008464 | PRKG2 | 0.0314 |
| ocu-cirR-novel-18685 | ENSOCUG00000009918 | SCARA3 | 0.0134 |
| ocu-cirR-novel-18685 | ENSOCUG00000011780 | CYFIP2 | 0.0389 |
| ocu-cirR-novel-18685 | ENSOCUG00000021980 | DNAJC14 | 0.0471 |
| ocu-cirR-novel-18685 | ENSOCUG00000023553 | GFI1 | 0.0370 |
| ocu-cirR-novel-18685 | ENSOCUG00000025244 | IGFBP7 | 0.0370 |
| ocu-cirR-novel-18686 | ENSOCUG00000003313 | LOX | 0.0314 |
| ocu-cirR-novel-18686 | ENSOCUG00000008071 | . | 0.0370 |
| ocu-cirR-novel-18686 | ENSOCUG00000008464 | PRKG2 | 0.0314 |
| ocu-cirR-novel-18686 | ENSOCUG00000009918 | SCARA3 | 0.0134 |
| ocu-cirR-novel-18686 | ENSOCUG00000011780 | CYFIP2 | 0.0389 |
| ocu-cirR-novel-18686 | ENSOCUG00000021980 | DNAJC14 | 0.0471 |
| ocu-cirR-novel-18686 | ENSOCUG00000023553 | GFI1 | 0.0370 |
| ocu-cirR-novel-18686 | ENSOCUG00000025244 | IGFBP7 | 0.0370 |
| ocu-cirR-novel-19049 | ENSOCUG00000003792 | THBS1 | 0.0086 |
| ocu-cirR-novel-19049 | ENSOCUG00000005439 | CCNJL | 0.0281 |
| ocu-cirR-novel-19049 | ENSOCUG00000007145 | KIAA0586 | 0.0491 |
| ocu-cirR-novel-19049 | ENSOCUG00000010200 | PMPCB | 0.0491 |
| ocu-cirR-novel-19049 | ENSOCUG00000010636 | XPNPEP2 | 0.0491 |
| ocu-cirR-novel-19049 | ENSOCUG00000017386 | RPL30 | 0.0247 |
| ocu-cirR-novel-19049 | ENSOCUG00000024349 | LOC100337909 | 0.0204 |
| ocu-cirR-novel-19076 | ENSOCUG00000001104 | RNF167 | 0.0027 |
| ocu-cirR-novel-19076 | ENSOCUG00000001414 | DDX23 | 0.0176 |
| ocu-cirR-novel-19076 | ENSOCUG00000001590 | KLC4 | 0.0417 |
| ocu-cirR-novel-19076 | ENSOCUG00000002871 | DNTT | 0.0417 |
| ocu-cirR-novel-19076 | ENSOCUG00000003209 | CCBE1 | 0.0036 |
| ocu-cirR-novel-19076 | ENSOCUG00000005822 | MROH8 | 0.0176 |
| ocu-cirR-novel-19076 | ENSOCUG00000006595 | UBA7 | 0.0274 |
| ocu-cirR-novel-19076 | ENSOCUG00000006714 | MMP2 | 0.0215 |
| ocu-cirR-novel-19076 | ENSOCUG00000007730 | LAD1 | 0.0215 |
| ocu-cirR-novel-19076 | ENSOCUG00000009260 | PLCH1 | 0.0215 |
| ocu-cirR-novel-19076 | ENSOCUG00000012034 | RBM47 | 0.0122 |
| ocu-cirR-novel-19076 | ENSOCUG00000012457 | GOLGB1 | 0.0215 |
| ocu-cirR-novel-19076 | ENSOCUG00000012754 | RUBCN | 0.0417 |
| ocu-cirR-novel-19076 | ENSOCUG00000012911 | CD4 | 0.0038 |
| ocu-cirR-novel-19076 | ENSOCUG00000013725 | FAM20A | 0.0062 |
| ocu-cirR-novel-19076 | ENSOCUG00000014252 | MAPKAPK2 | 0.0274 |
| ocu-cirR-novel-19076 | ENSOCUG00000015723 | SLC15A3 | 0.0286 |
| ocu-cirR-novel-19076 | ENSOCUG00000016580 | CTSB | 0.0122 |
| ocu-cirR-novel-19076 | ENSOCUG00000017308 | FLOT1 | 0.0176 |
| ocu-cirR-novel-19076 | ENSOCUG00000026966 | SASH3 | 0.0038 |
| ocu-cirR-novel-19076 | ENSOCUG00000028052 | AMOTL2 | 0.0122 |
| ocu-cirR-novel-19088 | ENSOCUG00000003186 | HPRT1 | 0.0324 |
| ocu-cirR-novel-19088 | ENSOCUG00000003209 | CCBE1 | 0.0324 |
| ocu-cirR-novel-19088 | ENSOCUG00000003863 | CD74 | 0.0491 |
| ocu-cirR-novel-19088 | ENSOCUG00000004136 | SEPT-7 | 0.0111 |
| ocu-cirR-novel-19088 | ENSOCUG00000004293 | EPS15 | 0.0491 |
| ocu-cirR-novel-19088 | ENSOCUG00000004451 | LIMS1 | 0.0204 |
| ocu-cirR-novel-19088 | ENSOCUG00000006714 | MMP2 | 0.0204 |
| ocu-cirR-novel-19088 | ENSOCUG00000008137 | MLLT3 | 0.0491 |
| ocu-cirR-novel-19088 | ENSOCUG00000008191 | GABRP | 0.0065 |
| ocu-cirR-novel-19088 | ENSOCUG00000008210 | CCR9 | 0.0111 |
| ocu-cirR-novel-19088 | ENSOCUG00000009055 | CD69 | 0.0491 |
| ocu-cirR-novel-19088 | ENSOCUG00000009261 | TMEM40 | 0.0241 |
| ocu-cirR-novel-19088 | ENSOCUG00000009504 | CD80 | 0.0491 |
| ocu-cirR-novel-19088 | ENSOCUG00000013564 | TRIM63 | 0.0491 |
| ocu-cirR-novel-19088 | ENSOCUG00000013725 | FAM20A | 0.0420 |
| ocu-cirR-novel-19088 | ENSOCUG00000016580 | CTSB | 0.0139 |
| ocu-cirR-novel-19088 | ENSOCUG00000017700 | PLS1 | 0.0247 |
| ocu-cirR-novel-19088 | ENSOCUG00000021388 | P2RX1 | 0.0139 |
| ocu-cirR-novel-19088 | ENSOCUG00000025121 | ALDH6A1 | 0.0204 |
| ocu-cirR-novel-19088 | ENSOCUG00000026567 | RLA-DMB | 0.0491 |
| ocu-cirR-novel-19088 | ENSOCUG00000029541 | ZNF157 | 0.0247 |
| ocu-cirR-novel-19136 | ENSOCUG00000000575 | HAVCR1 | 0.0491 |
| ocu-cirR-novel-19136 | ENSOCUG00000000787 | PTPRO | 0.0170 |
| ocu-cirR-novel-19136 | ENSOCUG00000001165 | RANBP2 | 0.0491 |
| ocu-cirR-novel-19136 | ENSOCUG00000002254 | DNMBP | 0.0204 |
| ocu-cirR-novel-19136 | ENSOCUG00000002528 | LRCH4 | 0.0491 |
| ocu-cirR-novel-19136 | ENSOCUG00000002924 | LGALS3 | 0.0420 |
| ocu-cirR-novel-19136 | ENSOCUG00000004035 | IRF6 | 0.0139 |
| ocu-cirR-novel-19136 | ENSOCUG00000005603 | TESPA1 | 0.0086 |
| ocu-cirR-novel-19136 | ENSOCUG00000006357 | ADAMTS12 | 0.0204 |
| ocu-cirR-novel-19136 | ENSOCUG00000007730 | LAD1 | 0.0204 |
| ocu-cirR-novel-19136 | ENSOCUG00000008983 | CTCF | 0.0170 |
| ocu-cirR-novel-19136 | ENSOCUG00000009261 | TMEM40 | 0.0241 |
| ocu-cirR-novel-19136 | ENSOCUG00000009761 | TMEM71 | 0.0491 |
| ocu-cirR-novel-19136 | ENSOCUG00000009863 | PRKCQ | 0.0170 |
| ocu-cirR-novel-19136 | ENSOCUG00000010061 | PLEKHG4 | 0.0086 |
| ocu-cirR-novel-19136 | ENSOCUG00000010887 | RPS6KA1 | 0.0204 |
| ocu-cirR-novel-19136 | ENSOCUG00000011780 | CYFIP2 | 0.0139 |
| ocu-cirR-novel-19136 | ENSOCUG00000013333 | HS3ST3A1 | 0.0241 |
| ocu-cirR-novel-19136 | ENSOCUG00000013498 | ADAMTS4 | 0.0472 |
| ocu-cirR-novel-19136 | ENSOCUG00000016254 | STAB2 | 0.0204 |
| ocu-cirR-novel-19136 | ENSOCUG00000016906 | . | 0.0204 |
| ocu-cirR-novel-19136 | ENSOCUG00000023896 | E2F2 | 0.0170 |
| ocu-cirR-novel-19136 | ENSOCUG00000024225 | GMPPA | 0.0491 |
| ocu-cirR-novel-19136 | ENSOCUG00000025119 | RELT | 0.0170 |
| ocu-cirR-novel-19136 | ENSOCUG00000025590 | LAT | 0.0491 |
| ocu-cirR-novel-19136 | ENSOCUG00000026206 | CCL4 | 0.0491 |
| ocu-cirR-novel-19136 | ENSOCUG00000029154 | . | 0.0420 |
| ocu-cirR-novel-19136 | ENSOCUG00000029399 | SH2D1B | 0.0170 |
| ocu-cirR-novel-19160 | ENSOCUG00000005439 | CCNJL | 0.0043 |
| ocu-cirR-novel-19160 | ENSOCUG00000009869 | LAMA3 | 0.0186 |
| ocu-cirR-novel-19160 | ENSOCUG00000016477 | SELP | 0.0314 |
| ocu-cirR-novel-19160 | ENSOCUG00000017386 | RPL30 | 0.0370 |
| ocu-cirR-novel-19160 | ENSOCUG00000022575 | VTN | 0.0246 |
| ocu-cirR-novel-19262 | ENSOCUG00000000109 | ADAMTS9 | 0.0494 |
| ocu-cirR-novel-19262 | ENSOCUG00000003911 | CD40LG | 0.0494 |
| ocu-cirR-novel-19262 | ENSOCUG00000016432 | OLFM4 | 0.0259 |
| ocu-cirR-novel-19262 | ENSOCUG00000021747 | WDR78 | 0.0494 |
| ocu-cirR-novel-19349 | ENSOCUG00000002898 | FBLN7 | 0.0281 |
| ocu-cirR-novel-19349 | ENSOCUG00000003089 | PRG3 | 0.0247 |
| ocu-cirR-novel-19349 | ENSOCUG00000003276 | PAX1 | 0.0491 |
| ocu-cirR-novel-19349 | ENSOCUG00000003863 | CD74 | 0.0491 |
| ocu-cirR-novel-19349 | ENSOCUG00000006320 | SMARCA2 | 0.0491 |
| ocu-cirR-novel-19349 | ENSOCUG00000006970 | SLC44A4 | 0.0247 |
| ocu-cirR-novel-19349 | ENSOCUG00000009047 | FCRL2 | 0.0491 |
| ocu-cirR-novel-19349 | ENSOCUG00000010247 | ATP6V0D2 | 0.0491 |
| ocu-cirR-novel-19349 | ENSOCUG00000010633 | HMOX1 | 0.0491 |
| ocu-cirR-novel-19349 | ENSOCUG00000012254 | SNAI2 | 0.0491 |
| ocu-cirR-novel-19349 | ENSOCUG00000015482 | TMEM241 | 0.0324 |
| ocu-cirR-novel-19349 | ENSOCUG00000015599 | GEMIN6 | 0.0491 |
| ocu-cirR-novel-19349 | ENSOCUG00000016268 | RCN1 | 0.0420 |
| ocu-cirR-novel-19349 | ENSOCUG00000016500 | . | 0.0491 |
| ocu-cirR-novel-19349 | ENSOCUG00000017021 | SLC30A3 | 0.0241 |
| ocu-cirR-novel-19349 | ENSOCUG00000025091 | AARD | 0.0003 |
| ocu-cirR-novel-19354 | ENSOCUG00000009504 | CD80 | 0.0247 |
| ocu-cirR-novel-19354 | ENSOCUG00000022021 | IL22RA2 | 0.0494 |
