# Supplementary material for: Comprehensive analysis of circRNA expression pattern and circRNA-miRNA-mRNA network in the pathogenesis of atherosclerosis in rabbits
Source: Aging (Albany NY). 2018 Sep 6;10(9):2266–83. doi: 10.18632/aging.101541 (PMC6188486; doi:10.18632/aging.101541)
Supplement: Supplementary Figure S1 [file aging-10-101541-s005.pdf]

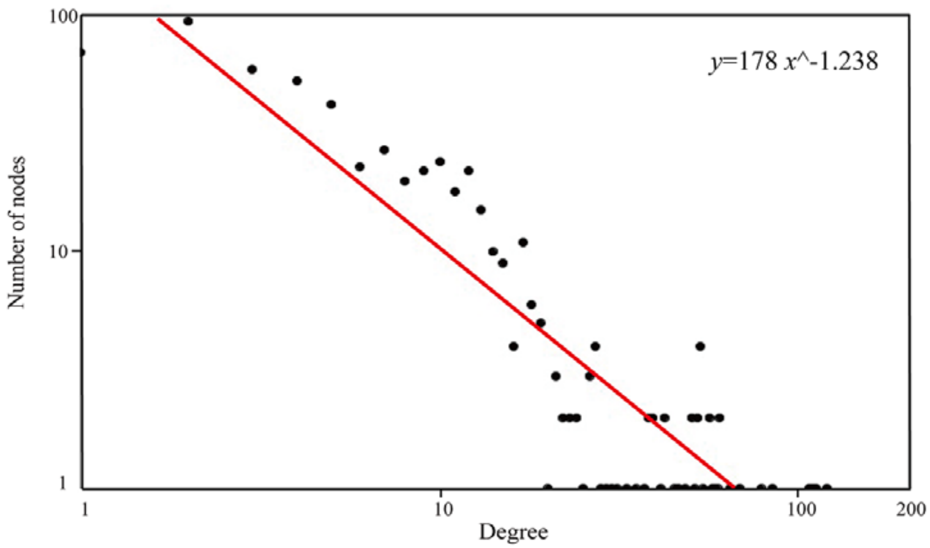

Supplementary Figure S1. The node degree distribution of DEcircRNA-DEmiRNA-DEmRNA triple network.
