# Supplementary material for: Comprehensive analysis of circRNA expression pattern and circRNA-miRNA-mRNA network in the pathogenesis of atherosclerosis in rabbits
Source: Aging (Albany NY). 2018 Sep 6;10(9):2266–83. doi: 10.18632/aging.101541 (PMC6188486; doi:10.18632/aging.101541)
Supplement: Supplementary Figure S2 [file aging-10-101541-s006.pdf]

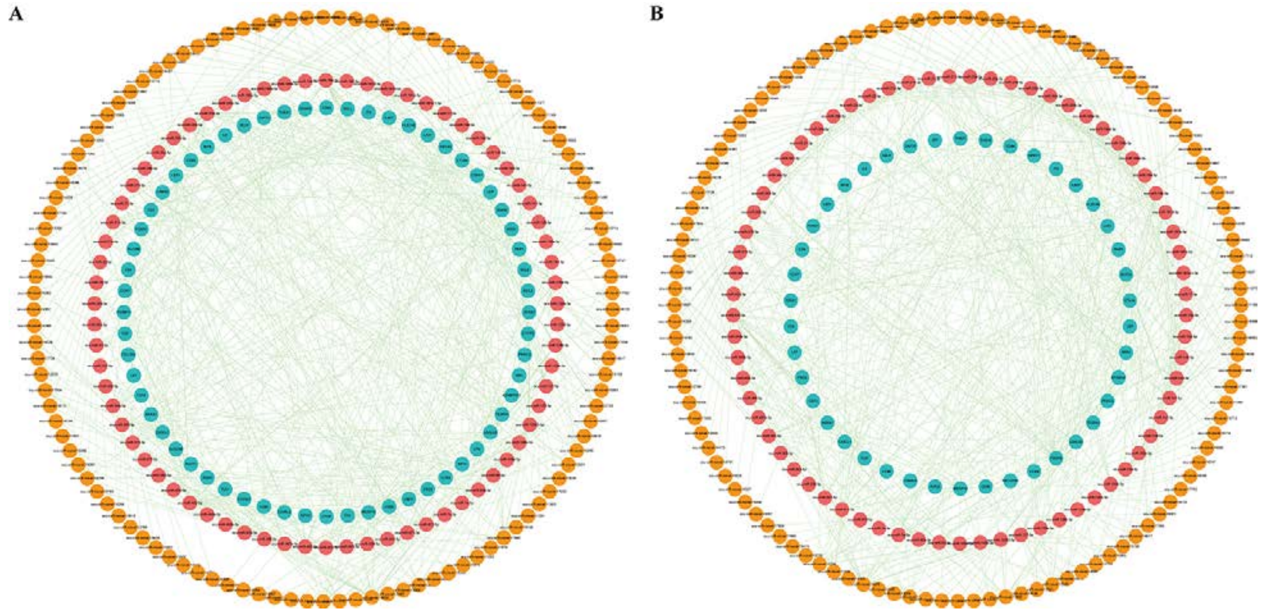

Supplementary Figure S2. The sub-network of DEcircRNA-DEmiRNA-DEmRNA triple network. (**A**) Cell adhesion sub-network. (**B**) Cell activation sub-network. The blue nodes represented mRNA, the red nodes represented miRNAs, and the orange nodes represented circRNAs.
